# Interfacial Dynamics Accelerate Aging Yet Sustain Toughness in Poly(l‑lactide) Block Polymer Plastics

**DOI:** 10.1021/acscentsci.6c00278

**Published:** 2026-05-11

**Authors:** Daniel M. Krajovic, Benjamin D. Chayet, Marc A. Hillmyer

**Affiliations:** † Department of Chemical Engineering and Materials Science, 5635 University of Minnesota, Minneapolis, Minnesota 55455, United States; ‡ Department of Chemistry, 5635 University of Minnesota, Minneapolis, Minnesota 55455, United States

## Abstract

Poly­(lactide) (PLA) homopolymer embrittles under ambient conditions within two days after melt processing through physical aging, which restricts its growth as a sustainable alternative to petroleum-derived, nondegradable plastics. Block polymers containing PLA and immiscible rubbery segments have shown promising mechanical longevity, though only at very high total molar masses. To elucidate the basic architectural and morphological features of such aging-resistant materials, we used a straightforward two-step synthetic route to generate a library of *n*-arm block polymer plastics (*n* = 1–4) with poly­(γ-methyl-ε-caprolactone) (PγMCL) as the rubbery core and poly­(l-lactide) (PLLA) as the outer block, fixing PLLA content at 80 wt %. Triblocks and star-blocks (i.e., *n* ≥ 2) exhibited high tensile toughness that persisted over long aging times even in samples with poorly entangled PLLA matrices. Crystallinity and architectural purity also promoted mechanical longevity. Calorimetry revealed that the most mechanically long-lived specimens, with *M*_PLLA_ < 35 kg mol^–1^, exhibited the fastest physical aging, which we ascribed to a coupling of block dynamics near the segregated PγMCL domain interfaces. Our results broaden the scope of viable block polymer architectures for PLLA mechanical longevity to more synthetically accessible macromolecules that are practically advantageous for scalability and melt processing.

## Graphical abstract

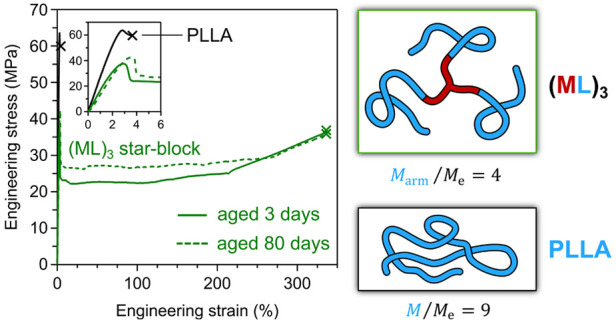

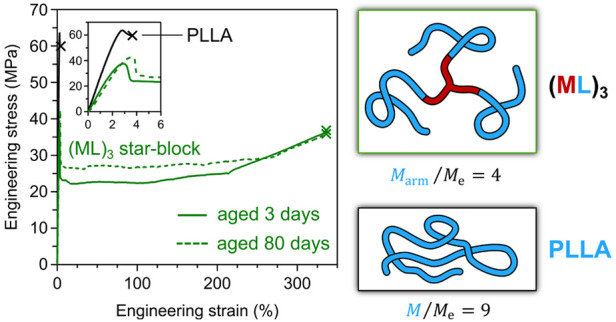

## 1 Introduction

Poly­(lactide), or PLA, is the forerunning sustainable plastic with a rapidly growing production capacity nearing the megaton scale.^1^ Its sourcing from biomass and industrial compostability offer a fully circular life cycle.^2,3^ With a desirably high stiffness (∼3 GPa) and tensile strength (∼65 MPa), PLA has seen most use in packaging, disposable consumer goods, and biomedical applications.^4,5^ However, PLA’s brittle nature, with a typical strain at break of <5% and Izod impact strength of only 1–2 kJ m^–2^,^6^ limits its further penetration in high-volume markets. As a result, many PLA toughening strategies have been developed. These include melt blending with flexible homopolymers^6–12^ and block polymers,^13–20^ melt deformation (i.e., stretching or orienting),^21–23^ plasticization,^24,25^ and statistical^26,27^ and block^28–37^ copolymerization of lactide with other monomers. These PLA materials have potential to compete with commercial plastics (e.g., poly­(ethylene), poly­(propylene), poly­(carbonate) (PC)) and even high-value engineering plastics (e.g., acrylonitrile butadiene styrene, Nylon, high-impact poly­(styrene)) in some cases.^38,39^

PLA-based materials require longevity in their toughness to be competitive with other commodity plastics. Interestingly, PLA is quite ductile immediately after melt processing, but embrittles rapidly (∼two days) under ambient conditions due to structural relaxation upon aging.^40^ PLA’s glass transition temperature (*T*_g_) (55–60 °C) makes it quite susceptible to embrittlement at ambient temperature over relatively short time scales. Below the *T*_g_, a polymer is in a nonequilibrium state with excess free energy and free volume.^41^ Over time, thermally activated cooperative motions of chain segments permit local structural relaxation that reduces free volume in an exothermic process, enhancing intermolecular interactions (e.g., dispersive, dipolar). Under an applied load, an entangled network of glassy chains must overcome these interchain interactions to undergo chain sliding, to produce a ductile, macroscopic shear yielding response.^42^ During physical aging, the stress required to initiate chain sliding eventually exceeds the crazing stress, leading to brittle failure unless other craze facilitation mechanisms are at play.^43^ Generally, the rate of physical aging is maximized within 10–20 °C of *T*_g_.^41^ For instance, even the archetypally tough plastic PC embrittles through physical aging after high temperature annealing (*T*_g_ ≈ 145 °C).^44–47^

Prior work on polymer blends offers valuable insights for the pursuit of mechanical longevity. Plasticization routes—wherein PLA is blended with a miscible polymer or small molecule—can lower *T*_g, PLA_ to near or below room temperature, and thus generate ductile materials that do not age under ambient conditions.^48–58^ However, the necessary plasticizer contents (≥20 wt %) substantially reduce stiffness and strength. Plasticizer migration or crystallization-driven expulsion are also limiting factors. Immiscible blends better balance stiffness and toughness. For example, Wang et al. found that adding 4,4’-methylenediisocyanate (MDI) as a chain extender/coupler to poly­(l-lactide) (PLLA)/starch blends improved interfacial compatibility, increasing toughness and mildly decreasing the rate of physical aging.^59^ By blending PLLA with a poly­(ether)-*b*-poly­(amide) thermoplastic elastomer, Liu et al. nucleated PLLA crystallization which in turn slowed physical aging by conformationally constraining the amorphous fraction of the matrix.^60,61^ While neat PLLA/poly­(butylene adipate-*co*-terephthalate) blends lose considerable ductility during aging, reactive compatibilization has improved mechanical longevity by widening the droplet-matrix interface.^62,63^

We previously investigated the mechanical longevity of two PLA-containing block polymers. Delgado and Hillmyer appended hydrogen bonding 2-ureido-4­[1*H*]-pyrimidinone groups to the ends of PLA-*b*-poly­(butadiene)-*b*-PLA triblock plastics, which modestly delayed embrittlement.^64^ Haugan et al. synthesized triblock and graft-block polymers based on poly­(γ-methyl-ε-caprolactone) (PγMCL) and atactic PLA.^37^ Reducing *f*_PLA_ from 0.9 to 0.8 and switching from a triblock to a graft-block architecture both improved mechanical longevity. These trends were ascribed to the “interconnectedness” of the rubbery, microphase separated PγMCL domains,^65^ with the triblock having discrete domains and the graft-blocks having more diffuse, extended domains, especially in samples with *f*_PLA_ = 0.8. No definitive conclusions were made about the origin of this mechanical longevity improvement, and the most long-lived graft-block specimen had an inconveniently large molar mass of 1,450 kg mol^–1^. Moreover, the use of noncrystallizable atactic PLA precluded any matrix crystallinity that could both slow aging and improve thermal resilience.

Motivated by our recent discovery of high levels of toughness and crystallinity in PLLA-*b*-PγMCL-*b*-PLLA (LML) triblocks,^29^ we sought to establish macromolecular design parameters for retaining these properties over practically long aging periods by systematically exploring *n*-armed (ML)_n_ block polymers (*n* = 1–4), focusing on the effects of arm and total molar masses, *M*_arm_ and *M*_tot_, at a fixed semicrystalline PLLA weight fraction (*w*_PLLA_) of 0.8. We found that (ML)_3_ and (ML)_4_ materials with low and easily manageable values of *M*_tot_, discrete PγMCL domains, and remarkably short, poorly entangled PLLA outer blocks best retain their high levels of toughness over long times. Calorimetric studies showed that the more rapid the physical aging, the better the mechanical longevity, which counters the typically reported correlation described above. We thus established an important design principle for practically circular, persistently tough, and thermally resilient PLLA block polymer plastics that will enable broader practical utility of PLA-based plastics.

## 2 Results and Discussion

### 2.1 Synthesis and Molecular Characteristics

Adapting Watts et al.’s original scheme,^66^ we first homopolymerized γMCL using the appropriate multifunctional initiators and then chain extended the resultant end-functionalized PγMCL with l-lactide, using Sn­(oct)_2_ to catalyze both steps (Schemes 1, S1). Starting with a (ML)_2_ triblock mimicking the highest-performing material from our previous work (M10-L38 in Scheme 1),^29^ we varied *n* along two orthogonal axes, one fixing *M*_tot_ and varying *M*_arm_, and the other fixing *M*_arm_ and varying *M*_tot_. Finally, to complete a series with increasing *M*_arm_ and *M*_tot_ at fixed *n* = 3, we synthesized (M24-L86)_3_, a large 3-arm star (Scheme 1). (Here and elsewhere, “M” = PγMCL, “L” = PLLA”, and # = *M*_n, NMR_ in kg mol^–1^ for the indicated block.)

**Scheme 1:**
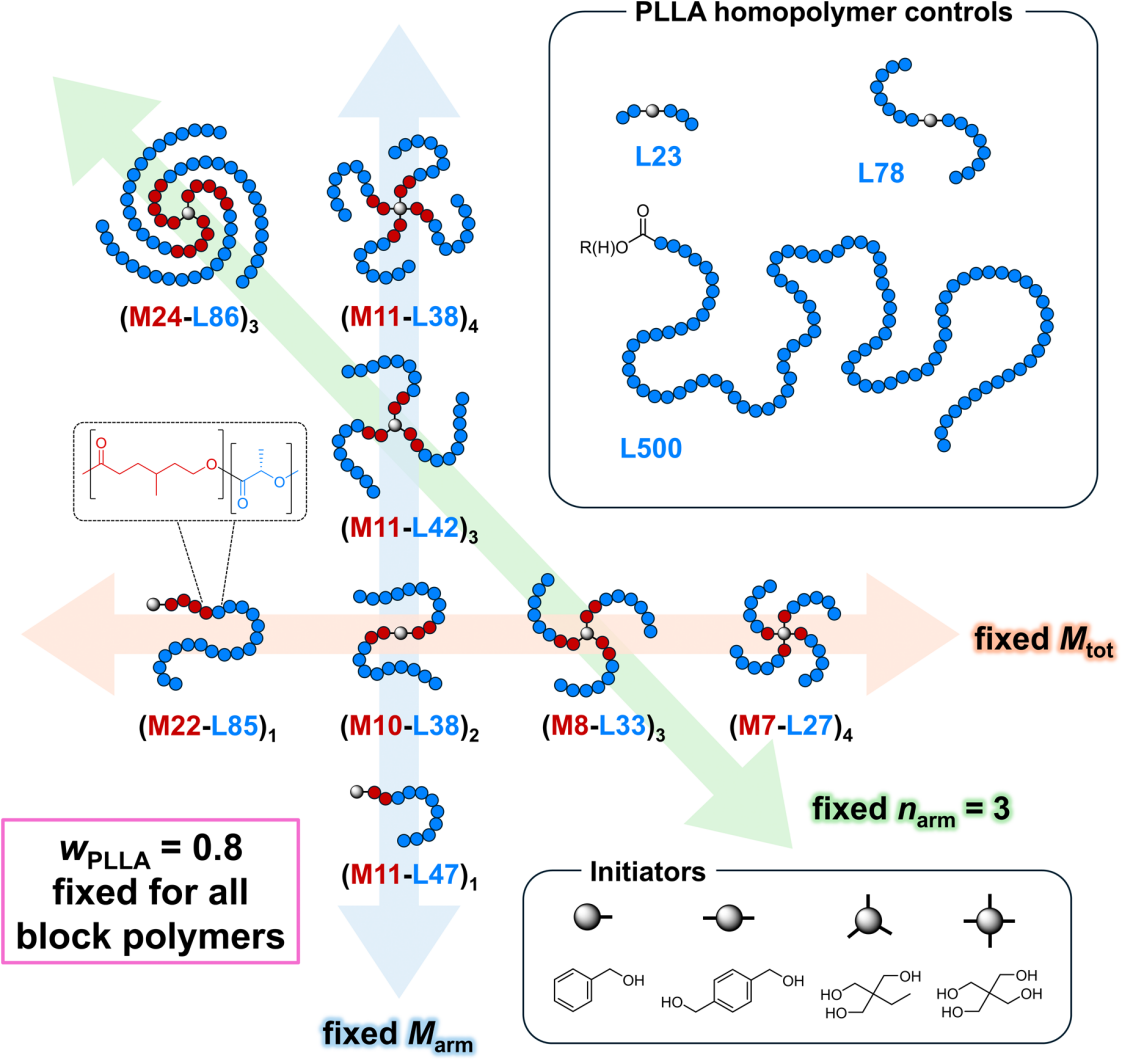
Libraries of (ML)_*n*_ Star-Blocks and PLLA Homopolymer Synthesized in This Study^a^

a Each circular bead in the chain cartoons approximately corresponds to 5 kg mol^–1^. L500 was synthesized without exogeneous initiator to maximize molar mass, and so, it was likely initiated either by water or an adventitious low molar mass alcohol as indicated in the scheme. “M” = PγMCL, “L” = PLLA, and # = *M*_*n*_ in kg mol^–1^ for the indicated block. The PγMCL blocks are shown in red, and the PLLA blocks are shown in blue.

All polymers gave ^1^H NMR data consistent with previous reports (Figures S1–S5), with all PγMCL telechelic hydroxyl groups consumed (Figure S6) and no evidence of repeat unit mixing in the PγMCL and PLLA blocks (Figure S7).^66,67^ Induction periods in the first step created some difficulty in controlling PγMCL arm lengths for *n* = 3 and *n* = 4 (see Figures S8–S10, Table S1–S2), but final *w*_PLLA_ values were tightly controlled from 0.78 to 0.81. In addition to the (ML)_*n*_ star-blocks, we synthesized three PLLA homopolymer controls denoted as L23, L78, and L500. Size exclusion chromatography (SEC) showed monomodal (M)_*n*_ and (ML)_*n*_ distributions, with clear shifts to lower retention times after the chain extension (Figure S11). Molar mass dispersities were below 1.5 for all species. We applied our previously reported purity analyses based on SEC (example in Figure S12) and ^1^H NMR spectroscopy^29^ to the (ML)_*n*_ products, estimating greater than 80 wt % architectural purity for all but (M11-L43)_2_* (74 wt %) and (M24-L81)_3_* (49 wt %) (Table S3). Based on ^1^H NMR features, the impurity species in these samples marked with * most likely included adventitiously initiated (ML)_*n*_ diblocks and PLLA homopolymer. Thus, we resynthesized these specimens with better drying of the γMCL monomer and PγMCL macroinitiators, producing much more pure analogs (M10-L38)_2_ (92 wt %) and (M24-L86)_3_ (93 wt %), allowing us to additionally explore the impact of architectural purity in this class of materials.

### 2.2 Thermal Characteristics

In Figure S13, thermogravimetric analysis (TGA) showed that the (ML)_*n*_ star-blocks did not undergo significant (i.e., 5%) mass loss until high temperatures (minimum *T*_d, 5%_ = 290 °C) indicating stability of these materials during melt-pressing at 190 °C. Figure S14 displays dynamic traces from differential scanning calorimetry (DSC), showing distinct *T*_g_ values for PγMCL (from −67 to −62 °C) and PLLA, the latter of which were only slightly depressed in the (ML)_*n*_ star-blocks (55–56 °C) relative to PLLA homopolymers (57–59 °C) (Figure S15). Typical crystallization (90–110 °C) and melting transitions (160–180 °C) for PLLA (Table S4) were also observed, and we found no consistent architectural trend in isothermal crystallization rates (Figure S16).

### 2.3 Morphological Characteristics

Figure 1 displays representative small-angle X-ray scattering (SAXS) patterns and atomic force microscopy (AFM) phase images of (M8-L33)_3_ and (M24-L81)_3_*. In the melt, (M8-L33)_3_ showed a sharp principal correlation peak (q*) in SAXS, along with broad but resolvable peaks at √3q* and √7q*, suggestive of strongly segregated, cylindrical PγMCL domains in the PLLA matrix with some long-range hexagonal (HEX) order (Figure 1a). Bragg peaks in wide-angle X-ray scattering (WAXS) appeared with PLLA crystallization at 110 °C (Figure S19a), at which point the higher-order SAXS peaks were absent, but the principal peak did not shift, consistent with loss of the mesostructural lattice but retention of the principal domain spacing. AFM phase imaging corroborated the SAXS patterns taken in either the melt or after rapid quenching between ice water-cooled plates (i.e., low crystallinity states), showing discrete, finely distributed PγMCL domains in the PLLA matrix. With a much larger *M*_tot_, (M24-L81)_3_* showed only a broad principal SAXS peak, a low-intensity shoulder at *q* = 0.28 Å^–1^, and the absence of higher-order peaks, indicating a more coarse, disordered arrangement of PγMCL domains, also supported by the corresponding AFM phase image (Figure 1b). Figures S17–S20 display the SAXS and WAXS patterns taken during staged cooling for all (ML)_*n*_ species.

**Figure 1:**
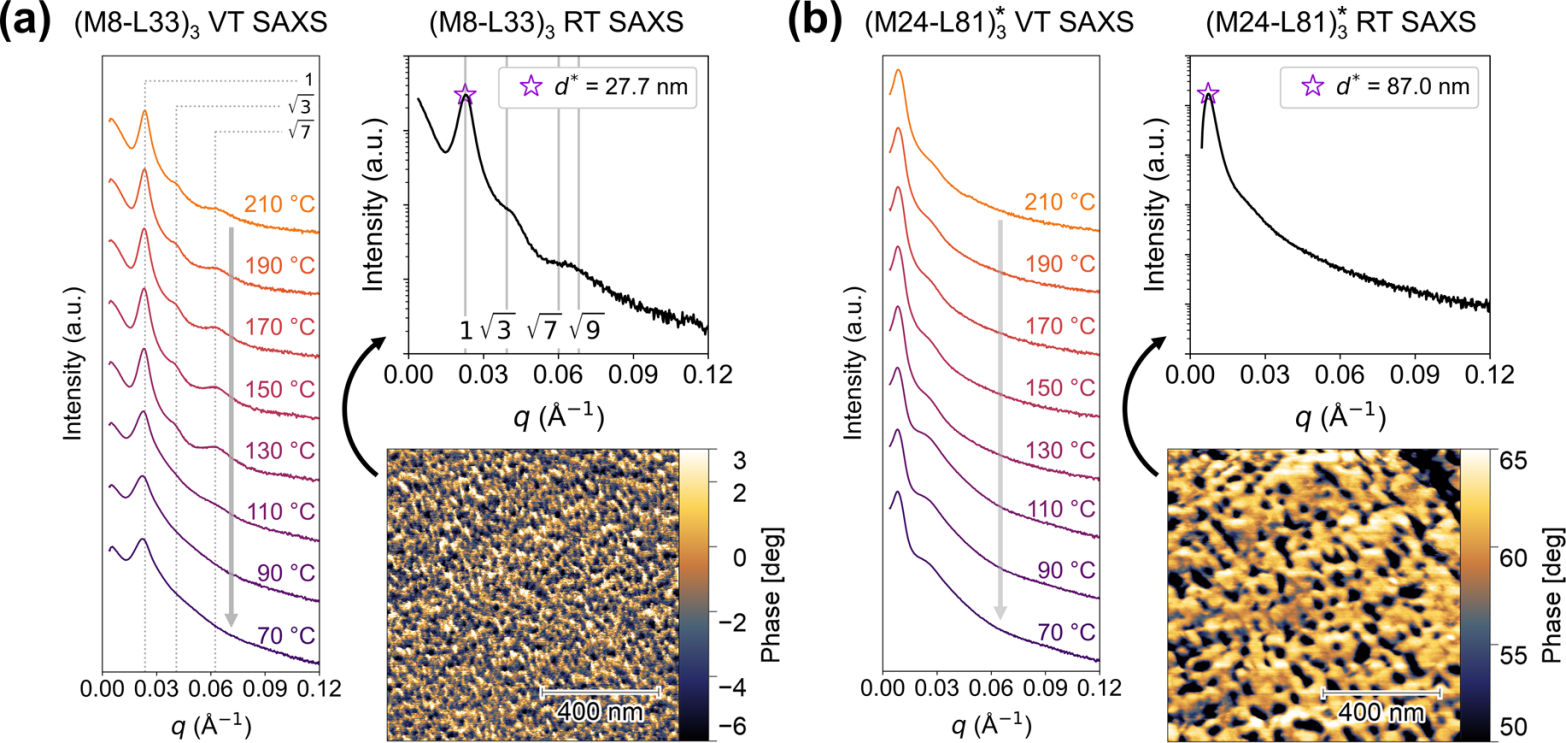
Morphological characterization of representative star-blocks (a) (M8-L33)_3_ and (b) (M24-L81)_3_*. In each panel, variable-temperature SAXS patterns obtained during staged cooling are shown on the left, and a room temperature SAXS pattern and corresponding AFM phase image (AC mode) from rapidly quenched specimens are shown on the right. In the AFM phase images, the dissipative, low-phase PγMCL domains are dark, and the glassy, high-phase PLLA matrix is bright.

We calculated the segregation strength, (*χN*)_arm_, at each temperature (χ­(*T*) = 51.6 *T* ^–1^ – 0.07, *V*_ref_ = 118 Å^3^, ρ_PγMCL_ = 1.037 g cm^–3^, ρ_PLLA_ = 1.25 g cm^–3^).^66,68^ While all materials were moderately to strongly segregated (Figure S21),^69,70^ SAXS features generally intensified with lower *M*_arm_ and lower *M*_tot_, likely due to the ability of these less entangled materials to more readily adopt equilibrium morphologies. Additionally, while (M10-L38)_2_ showed SAXS peaks at √3q* and √7q*, indicating greater long-range order than its less pure congener, (M11-L43)_2_* (Figure S18), this was not true of the analogous pair of (M24-L86)_3_ and (M24-L81)_3_* which both lacked long-range order (Figure S19c-d). These results support our earlier assertion^29^ that both melt diffusion kinetics and impurity content constrain morphological order in the (ML)_*n*_ system.

Recalling the mechanical consequences of diffuse domains in Haugan et al.’s study, we estimated the PγMCL-PLLA interfacial width (*w*_I_) by analyzing the high-*q* regime of the room temperature SAXS patterns (Figure S23; see details of the Porod analysis in the Supporting Information). Six of the ten (ML)_*n*_ star-blocks produced statistically meaningful *w*_I_ values ranging from 0.8 to 1.9 nm, lacking any correlation with *M*_arm_, while the other four showed even sharper interfaces (Figure S24a). These values are consistent with theoretical predictions of ∼1 nm interfacial widths, both in the mean-field limit and when accounting for finite degrees of polymerization.^71,72^ Thus, for all materials studied, the interfacial monomer composition profile was quite sharp, again consistent with the estimated segregation strengths.

### 2.4 Mechanical Characteristics

We surveyed all (ML)_*n*_ star-blocks’ uniaxial tensile behavior after 3 days and 80 days of room temperature (21–23 °C) aging and with two thermal histories: (1) rapid quenching from the melt to minimize—but not eliminate—crystallinity and (2) melt crystallization at 100 °C for 5 min to induce significant (>35%) PLLA crystallization (Tables S5–S7, Figures S25–S36). (We determined the specimens’ crystallinities using DSC—see Figure S37—and noted 35% crystallinity as a threshold for meaningful improvements in the heat distortion temperature.^29,73^ ) The initial three-day time point is longer than the native ambient embrittlement time of PLLA homopolymer.^40^ All (ML)_*n*_ materials showed relatively high Young’s moduli values ranging from 1.6–2.0 GPa.^74^ For completeness, we evaluated the stress–strain curves for representative diblocks (M11-L47)_1_ and (M22-L85)_1_ (Figure S38) and they were largely brittle, consistent with results from related systems.^75–77^

In the fixed *M*_tot_ series (Figure 2a), the triblock (M10-L38)_2_ lost nearly half its three-day-aged toughness (75 MJ m^–3^) after 80 days (40 MJ m^–3^). Samples (M8-L33)_3_ and (M7-L27)_4_ with more, but shorter arms not only showed the highest toughnesses of all quenched materials (86 and 74 MJ m^–3^, respectively), but also largely retained their toughness after 80 days (90 and 66 MJ m^–3^, respectively). These two materials performed remarkably well for having such low PLLA block entanglement numbers (*M*_n, arm, PLLA_/*M*_e, PLLA_) of 3.8 and 3.1, respectively;^78^ PLLA homopolymers at these molar masses are brittle immediately after processing due to the weakly entangled amorphous network. Increasing *n* from 2 to 4 in the fixed *M*_arm_ series had no significant effect on toughness or longevity (Figure 2b), while increasing *M*_tot_ in the fixed *n* = 3 series led to a slight reduction in average mechanical longevity (Figure 2c).

**Figure 2:**
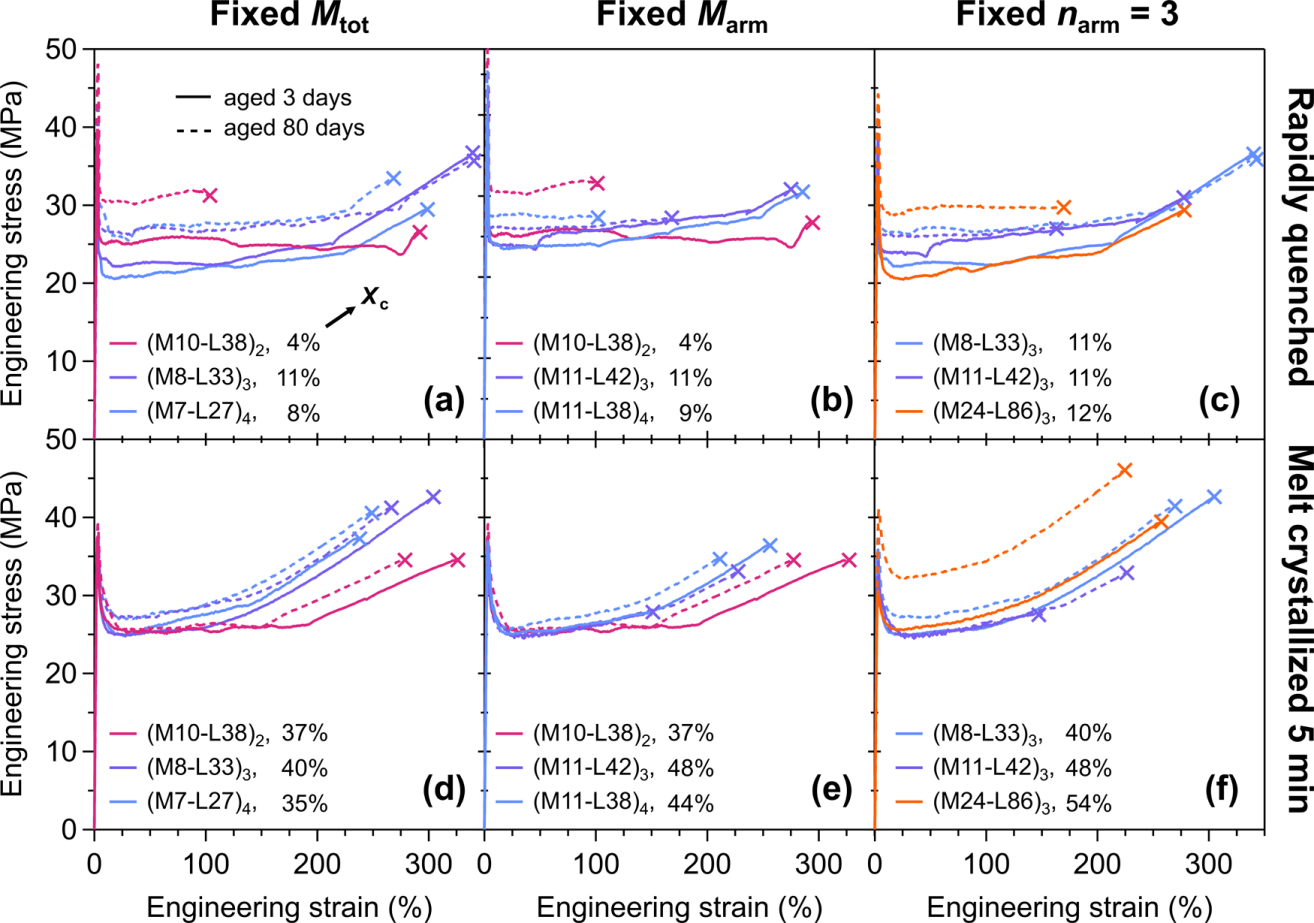
Representative stress–strain curves obtained from uniaxial tensile testing of (ML)_*n*_ star-blocks with *n* ≥ 2. Panels (a)-(c) respectively show the curves for the fixed *M*_tot_, *M*_arm_, and *n*_arm_ = 3 series materials in a low-crystallinity state induced by rapidly quenching the molten films to room temperature. Panels (d)-(f) show the curves for these materials in a state of higher crystallinity induced by intentional annealing at 100 °C for 5 min. The percentage next to each sample label indicates its crystallinity. Solid lines = 3 days aged; dashed lines = 80 days aged.

The performance of (M8-L33)_3_ and (M7-L27)_4_ demonstrates that with 20 wt % of PγMCL rubber and with low *M*_arm_, these (ML)_*n*_ star-blocks with low levels of crystallinity and discrete, moderately to well-ordered domains can achieve excellent and long-lived toughness with more than an order of magnitude lower molar masses than Haugan et al.’s graft-block.^37^ Hence, mechanical longevity does not require a diffuse, interconnected rubber domain morphology in this class of materials. These results open the design space to much smaller block polymers that are more advantageous for practical melt processing.

Increased matrix crystallinity in these samples bolstered mechanical longevity. After melt crystallization, (M10-L38)_2_’s high-crystallinity sample retained 85% of its initial toughness at 80 days (Figure 2d) compared to 53% for the low crystallinity sample (Figure 2a). The melt-crystallized, high-crystallinity star-block samples (M8-L33)_3_ and (M7-L27)_4_ also had higher toughnesses than their quenched counterparts and likewise excellent toughness retention. In the fixed *M*_arm_ series, (M11-L38)_4_ improved from 56% to 76% retention (Figure 2e), and in the fixed *n*_arm_ series, (M24-L86)_3_’s retention modestly improved from 82 to 87% (Figure 2f). We also tested the *n* ≥ 2 subset of the fixed *M*_tot_ series at much longer aging times of 7–10 months (Figure S39). In a low crystallinity state, these materials were much less tough over these long aging times, but they nonetheless retained average strains at break ranging from 73 to 168% (Table S5; last rows of Figures S28, S29, and S34). In contrast, high levels of crystallinity consistently preserved toughness over this extended period for all specimens (Tables S6–S7).

The yielding behavior provided some insight into the influence of crystallinity on mechanical longevity. During aging of amorphous PLLA chains, densification impedes chain sliding, increasing the yield stress. While crystallization depletes amorphous material, the mobile amorphous fraction that remains can still age after crystallization, and so high crystallinity does not guarantee mechanical longevity. High PLLA crystallinity in these samples did not produce as much increase in yield stress with prolonged aging that was observed in the low-crystallinity samples (Figure S40). Furthermore, increasing crystallinity quantitatively decreased yield stress in (M8-L33)_3_ and (M7-L27)_4_ (Figure S41). These results indicate that the slippage and rotation of matrix lamellae^79^ provide a dissipation mechanism with a lower yield stress largely unaffected by aging-induced densification of the mobile amorphous fraction. *In situ* tensile X-ray scattering and *ex situ* DSC also verified that the triblock and star-block architectures had similar low- and high-crystallinity (ML)_*n*_ deformation mechanisms^29^ (Figures S42–S43). Finally, by comparing the tensile behavior of (M11-L43)_2_* and (M24-L81)_3_* with their purer analogs (M10-L38)_2_ and (M24-L86)_3_, we found that macromolecular impurities at this level compromised both toughness and longevity (Figures S44–S45).

To summarize, all (ML)_*n*_ materials (*n* ≥ 2) showed appreciable toughness and longevity enhancements as compared to PLLA homopolymer. The *n* ≥ 3 samples (M8-L33)_3_ and (M7-L27)_4_, with the smallest *M*_arm_ values, offered a slight toughness improvement in the low-crystallinity state over the triblock. This is surprising considering the low matrix entanglement for the PLLA segments in these star-block samples (*M*_arm, PLLA_ ≈ 30 kg mol^–1^). Moreover, the relatively low *M*_tot_ (∼130 kg mol^–1^) in these two star-block samples is most favorable for melt processing compared to higher molar mass polymers. In addition, all specimens remained tough after extensive matrix crystallization. Thus, the (ML)_*n*_ platform offers mechanically tough, long-lived, and thermally resilient PLLA plastics by established and straightforward synthetic and processing protocols. We next sought mechanistic insights into such impressive aging resistance from enthalpy relaxation studies.

### 2.5 Enthalpy Relaxation Characteristics

After erasing thermal history and rapidly quenching below *T*_g, PLLA_, we isothermally annealed the samples at *T*_a_ = 45 °C (≈ *T*_g, PLLA_ – 10 °C). We then determined the enthalpy recovery upon reheating through *T*_g, PLLA_ as a function of annealing time (*t*_a_) and normalized it by the theoretical value at equilibrium to produce what we refer to as “fractional physical age.” Under this formalism, all samples approach a fractional age of one at infinite time (see description of DSC experiments in the Supporting Information for more details).

Figure 3a displays a representative data set (see Figure S47 for all reheating curves), and Figures 3b-e compare the aging behavior of the (ML)_*n*_ star-blocks and three PLLA homopolymer control samples. All polymers exhibited the expected semilogarithmic relationship^41^ between enthalpy relaxation and the aging time. We fitted these data, extracting a slope (*i.e.*, aging rate) and the value interpolated at *t*_a_ = 1 h, which we refer to as “initial age,” such that differences in initial age encompass differences in aging that the samples undergo during and just after the rapid quenching prior to the longer isothermal periods. While the aging rates exhibited little variation within (ML)_*n*_ materials, the initial age of all specimens increased with decreasing *M*_arm_ (Figures 3b-d). LML triblocks from our previous report with both lower and higher *w*_PLLA_ ^29^ exhibited the same initial age dependence on *M*_arm_ (Figure S48). Combined, these results suggest that *M*_arm_ alone controlled the initial age. Notably, all the (ML)_*n*_ star-blocks had higher initial ages and aging rates than the PLLA homopolymer controls (Figure 3e). Thus, the (ML)_*n*_ architecture remarkably enables excellent mechanical longevity even though the PLLA in those samples reaches a fractional physical age that would render analogous homopolymers brittle.

**Figure 3:**
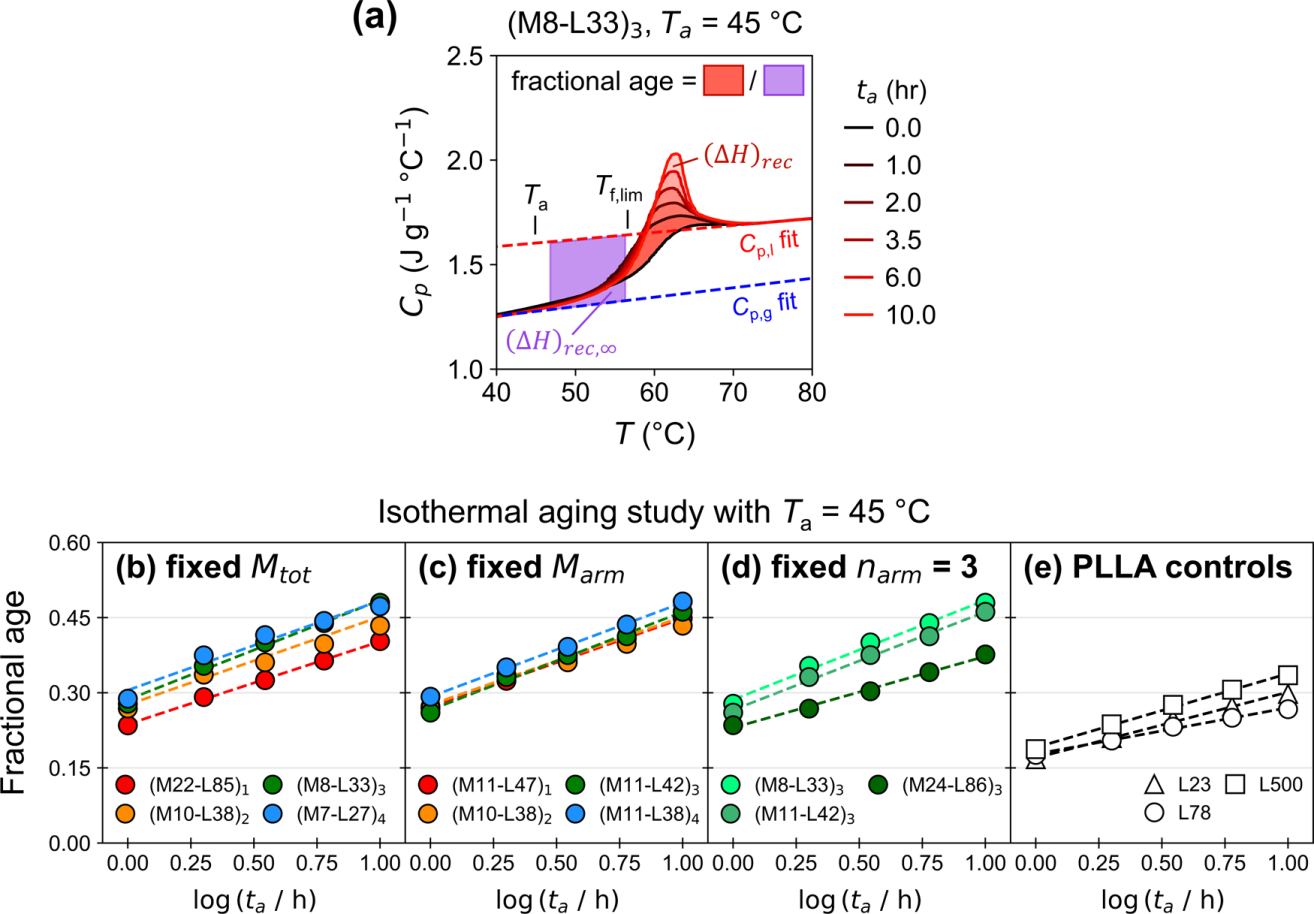
(a) Reheating curves recorded for (M8-L33)_3_ after various durations of annealing at 45 °C. Fractional physical age plotted against the logarithm of aging time for the (b) fixed *M*_tot_, (c) fixed *M*_arm_, (d) fixed *n*_arm_ = 3, and (e) PLLA homopolymer control series. The dashed lines show linear fits. The initial age is defined as the fractional age fitted at *t*_*a*_ = 1 h.

To connect this aging behavior with mechanical properties, we conducted two additional isothermal studies on rapidly quenched (i.e., low crystallinity) specimens. First, we compared the aging behavior of (M8-L33)_3_ and L78 measured at room temperature (*T*_a_ ≈ 22 °C), a lower aging temperature farther from *T*_g, PLLA_. Again, (M8-L33)_3_ showed a higher initial age and aging rate (Figure S52). Second, we also explored mechanical behavior after higher-temperature annealing (*T*_a_ = 45 °C) and observed that the (M8-L33)_3_ samples showed excellent toughness retention at four- and eight-day time points despite fractional physical ages of 0.73 and 0.76, respectively (Figure S53). Based on semilogarithmic fits from the room temperature study, such a high fractional age of the PLLA aged at *T*_a_ = 45 °C would be reached only after over three decades of ambient aging (*T*_a_ ≈ 22 °C). PLLA homopolymer, meanwhile, embrittles at a fractional age of <0.2. Thus, (M8-L33)_3_ demonstrated remarkable mechanical performance after both long-time ambient and shorter-time accelerated aging. However, because rapidly quenched (M8-L33)_3_ ultimately lost 78% of its toughness after 10 months of actual ambient aging (Table S5, Figure S38), these results suggest that the aging temperature influences the mechanical consequences of structural relaxation, and so accelerated testing in these materials should be treated cautiously.

We also performed an aging study in which we varied the cooling rate (*q*_1_) through *T*_g, PLLA_ to quantify the structural relaxation allowed over different experimental time scales (Figure S54). Because this method focuses on relaxations occurring in the vicinity of *T*_g, PLLA_, it was well-suited to compare the observed differences in the initial age described above. For each *q*_1_, we calculated the limiting fictive temperature (*T*_f, lim_), or the temperature at which the segmental motions effectively cease at a particular cooling rate, prohibiting further structural relaxation.^80^ A linear fit of ln­(*q*_1_) v. *T*_f, lim_^–1^ (Figure S55) provides a volume-average aging activation energy, Δh*, as the slope.^81^ Figures 4a and 4b respectively display the reheating curves and the activation energy analysis for (M8-L33)_3_ as an example. The PLLA homopolymers’ activation energies ranged from about 800 to 1050 kJ mol^–1^ (Figure 4c), reasonably close to Pan et al.’s reported value of 1107 kJ mol^–1^.^40^ In the (ML)_*n*_ block polymers, decreasing *M*_arm_ led to smaller values of the average activation energy for aging and thus accelerated segmental relaxation (Figure 4c). This finding corroborates the higher initial ages of the PLLA in the block polymers as compared to the PLLA homopolymers we observed in the isothermal aging study (Figure 3b-e).

**Figure 4:**
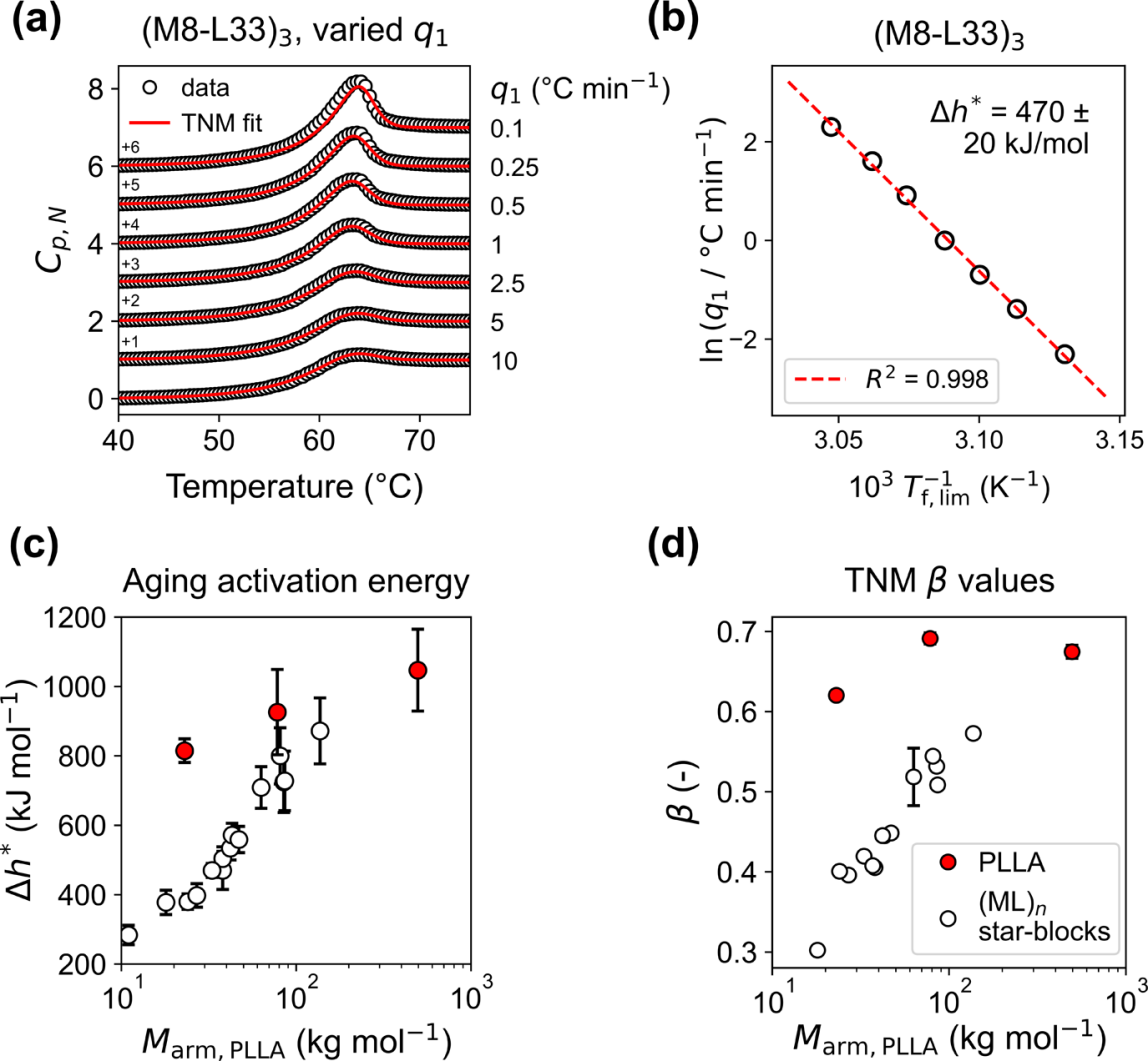
(a) Reheating curves for (M8-L33)_3_ after cooling at the listed rates. The data are presented as normalized heat capacity: *C*_p,N_ = (*C*_p_(*T*) – *C*_p,g_(*T*))/(*C*_p,l_(*T*) – *C*_p,g_(*T*)). Fits to the TNM model are superposed on the data as red lines. (b) Aging activation energy analysis for (M8-L33)_3_. (c) Aging activation energy and (d) fitted β values from the TNM model plotted against *M*_arm, PLLA_. Error bars in (c) indicate 95% confidence intervals and in (d) indicate standard errors.

We also fitted the varied *q*_1_ data shown in Figure 4a to the Tool-Narayanaswamy-Moynihan (TNM) aging model,^82^ which predicts the evolution of *T*_f_ across time and temperature as a superposition of discrete structural relaxations (see the Supporting Information for more details). During cooling, each decrement in *T*_f_ occurs over time according to a stretched exponential function, wherein the stretching exponent, β, characterizes the width of the distribution of relaxation times, with a lower β signifying a broader distribution. Figure 4a shows the TNM fits to the (M8-L33)_3_ data at the various reheating rates *q*_1_ (the fits for all other materials are shown in Figure S56). Figure 4d shows that the β values extracted from these fits correlated strongly with log­(*M*_arm, PLLA_) in the star blocks, mirroring Δh* determined from the *T*_f,lim_ analysis (Figure 4c). The PLLA homopolymers showed higher β values—as evinced from much sharper enthalpy recovery peaks—than all (ML)_*n*_ materials, signifying narrower distributions of segmental relaxation times. While the *T*_f,lim_ analysis reports on the volume-average value of the Δh* distribution, the TNM model reports on the width of that distribution through β. Evidently, tethering increasingly short PLLA blocks to PγMCL (i) reduced the volume-average aging activation energy (lower Δh* from *T*_f,lim_ analysis) and (ii) broadened the distribution of activation energies (lower β).

### 2.6 Mechanistic Interpretations and Implications for Materials Design

Covalently tethering chains to rigid substrates (*T*_g, substrate_ > *T*_g, chain, bulk_) slows their segmental dynamics and locally increases their *T*_g_,^83–85^ while tethering to soft substrates (*T*_g, substrate_ < *T*_g, chain, bulk_) speeds segmental motions and decreases *T*_g_.^86^ This phenomenon operates at domain interfaces in block polymers. As an example, Goswami et al. demonstrated that tethering poly­(ethylene oxide) to a less mobile PS block slowed its intermediate Rouse modes.^87^ Furthermore, Priestley et al. showed that a rubbery poly­(*n*-butyl methacrylate) block reduced the *T*_g_ of its glassy poly­(methyl methacrylate) (PMMA) co-block within 4 nm of the domain interface by ∼10 K more than predicted from segmental mixing and soft confinement, evincing a dominant contribution of glassy–rubbery chain connectivity.^88,89^

We expect that PLLA-PγMCL mobility coupling similarly occurs in our system; PLLA segmental relaxations are accelerated near the PγMCL domains and lead to a *T*_g, PLLA_ profile monotonically increasing toward the bulk PLLA *T*_g_ value with increasing distance from the rather sharp PγMCL domain interface (Figure 5). In this *T*_g_ boundary layer, the facilitated segmental motions thus speed enthalpy relaxation relative to PLLA homopolymer. Priestley et al. reported that *χN* does not control the width of the *T*_g_ profile,^89^ and so we expect that the width of the *T*_g, PLLA_ gradient is strictly an interfacial quantity and does not increase with the domain spacing. (See the Supporting Information—Figures S24b, S24d, and S58–S63—for SAXS and AFM measurements that support this conclusion for our system). Hence, smaller-*M*_arm_ (ML)_*n*_ star-blocks must have a greater portion of their matrix dynamics associated with interfacial mobility coupling—that is, the purple interfacial region of fixed width *z*_I_ occupies a larger fraction of the blue matrix as shown by comparison of the panels of Figure 5—accounting for the strong *M*_arm_ dependence of the distribution of segmental relaxation times, wherein higher *M*_arm_ values produce larger β values and thus a narrower distribution of segmental relaxation times (Figure 4d).

**Figure 5:**
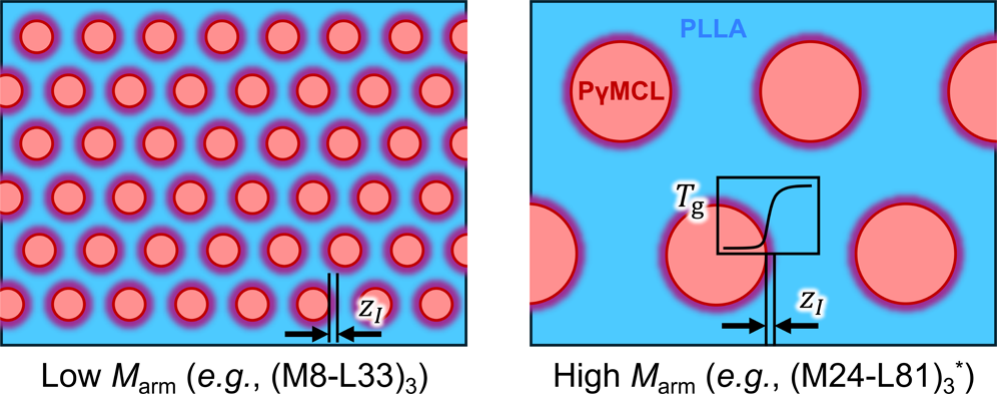
Two-dimensional cartoon of (ML)_*n*_ domain morphology, idealized with HEX order and *w*_PLLA_ = 0.8, identifying the width of the *T*_g_ gradient, *z*_I_, and showing the qualitative behavior of *T*_g_ across the block domain interface. PγMCL domains are shown in red, and the PLLA matrix is shown in blue. Both panels have the same *z*_I_ of 4 nm, and the depicted domain spacings are true to SAXS measurements using this scale.

We are ultimately interested in determining how interfacial dynamics could influence mechanical longevity. The Roth group recently reported that the shear modulus of a PS/poly­(butadiene) blend resembles the *T*_g_ profile across the polymer interface in a logarithmically symmetric fashion.^90,91^ We hypothesize that a similar shear modulus profile exists in the (ML)_*n*_ mesostructure, varying from *G*_PγMCL_ ≈ 0.9 ΜPa^66^ at the center of the rubbery domains to *G*_PLLA_ ≈ 1 GPa (*E*/3) in the rigid matrix far from the domain interfaces. Such a shear modulus gradient would help to delocalize stress that typically concentrates near the domain interfaces,^92^ in turn encouraging strain delocalization to facilitate shear yielding even when the bulk matrix, farther from the interface, has reached a relatively high physical age that would lead to embrittlement in PLLA homopolymer.

The emergent longevity of more easily processable specimens with low *M*_arm, PLLA_ naturally suggests the question, “How small is too small?” In our previous work on triblocks,^29^ the specimen that best balanced stiffness and toughness was (M11-L37)_2_, with *w*_PLLA_ = 0.78. Halving the midblock length to 11 kg mol^–1^ at fixed composition produced (M5.5-L24)_2_, and this sample showed only one-tenth the toughness. Additionally, the yield stress in this lower molar mass specimen was 9 MPa higher, indicating that the strain delocalization mentioned above was more difficult despite the comparatively greater fraction of the PLLA influenced by block interfacial dynamics. Moreover, matrix crystallization did not improve the (M5.5-L24)_2_’s toughness to match that of (M11-L37)_2_. Hence, (M5.5-L24)_2_ showed what is “too small” for triblocks. Here, (M7-L27)_4_ can be thought of as a self-coupling of (M5.5-L24)_2_ at the block midpoint creating the corresponding star polymer of about double the molar mass. Clearly, this combined architectural change and *M*_tot_ increase massively improved toughness and endowed the material with significant mechanical longevity. However, such combined architecture changes and molar mass increases in higher-*M*_tot_ samples did not improve upon the toughness or longevity of the triblock (compare samples (M10-L38)_2_, (M11-L42)_3_, and (M11-L38)_4_ in Figures 2b, 2e). So, at high enough molar mass, the added complication of preparing a star-block architecture is unnecessary. While the star-block architecture may offer slight toughness and longevity benefits in the low-crystallinity state (Figure 2a), melt crystallization endows the entire fixed *M*_tot_ series (*n* ≥ 2) with thermal resilience, high toughness, and excellent longevity to be sure (Figure 2e). In addition, the block polymers’ intrinsic interfacial mobility coupling offers competitive mechanical properties even with compositionally sharp domain interfaces in all samples.

## 3 Conclusions

The (ML)_*n*_ star-block platform has achieved long-lived toughness in PLLA-based plastics. Moreover, using this platform, we showed that rapid physical aging of the PLLA outer blocks does not preclude mechanical longevity, contrary to behavior in PLLA homopolymer. Three-and four-arm star-blocks with surprisingly low *M*_arm, PLLA_, more than an order of magnitude smaller in *M*_tot_ than a competitive previous iteration, exhibited the highest and most long-lived toughness despite poor matrix entanglement. Calorimetry experiments showed that the tethered PγMCL core blocks accelerated the segmental dynamics of the PLLA outer blocks. This acceleration reduced the average PLLA aging activation energy and broadened the activation energy distribution, leading to (ML)_*n*_ star-blocks attaining greater intrinsic physical age than PLLA homopolymers after identical thermal histories. The strength of this effect correlated inversely with *M*_arm_, and so materials with a smaller *M*_arm_ had greater portions of their matrices influenced by interfacial dynamics. We postulate that the coupled dynamics facilitated stress delocalization to support ductility, counteracting the loss of free volume in the PLLA matrix during aging. Crystallinity and high macromolecular purity also enhanced mechanical longevity. PLLA-rich (ML)_*n*_ block polymers at synthetically manageable molar masses optimize thermal and mechanical characteristics while minimizing synthetic complexity. Our materials simultaneously meet benchmarks of toughness, thermal resilience, and mechanical longevity set by commercial plastics (Table S8), advancing PLLA toward its highest potential as a circular alternative for petroleum-derived plastics in high-volume packaging applications.

## Supplementary Material



## Data Availability

All primary data files are available free of charge at https://hdl.handle.net/11299/166578.

## Notes

Materials and methods, synthetic protocols, sample preparation and characterization methods, data analysis methods, NMR spectra, SEC traces, tabulated macromolecular characteristics, TGA traces, DSC traces, 1D SAXS and WAXS patterns from variable-temperature and room temperature scans, AFM images, tabulated tensile characteristics, tensile stress–strain curves and sample images, 2D images from *in situ* tensile SAXS/WAXS experiments, reheating heat capacity traces from isothermal and dynamic enthalpy relaxation studies, heat capacity determination data sets from quasi-isothermal modulated DSC, activation energy and TNM fits for dynamic studies, and plots of fitted parameters against *M*_arm, PLLA_ (PDF)
