# Supplementary material for: Interfacial Dynamics Accelerate Aging Yet Sustain Toughness in Poly(l‑lactide) Block Polymer Plastics
Source: ACS Cent Sci. 2026 May 11;12(5):684–94. doi: 10.1021/acscentsci.6c00278 (PMC13220197; doi:10.1021/acscentsci.6c00278)
Supplement: Supplementary file 1 [file oc6c00278_si_001.pdf]

Supporting Information for

"Interfacial dynamics accelerate aging yet sustain toughness in poly(L-lactide) block polymer plastics"

Daniel M. Krajovic,[†] Benjamin D. Chayet[†], and Marc A. Hillmyer[*,‡]

[†]Department of Chemical Engineering and Materials Science, University of Minnesota, Minneapolis, Minnesota 55455, United States

[‡]Department of Chemistry, University of Minnesota, Minneapolis, Minnesota 55455, United States

*Corresponding author (email address: hillmyer@umn.edu)

Experimental

Materials

L-lactide was generously provided by NatureWorks, LLC, recrystallized from anhydrous ethyl acetate (3x) and then from anhydrous toluene (3x) under an argon atmosphere, and dried for 48 hours at 80 °C under vacuum (25 mTorr). γ-methyl-ε-caprolactone (γMCL) was purchased from Renewable Solutions, LLC and purified through fractional distillation under dynamic vacuum at 200-1,000 mTorr and 70-100 °C. Benzyl alcohol (BnOH; anhydrous, 99.8%) was purchased from Sigma Aldrich and used without further purification. 1,4-benzenedimethanol (BDM) was purchased from Alfa Aesar, recrystallized three times in toluene, sublimed overnight at 100 °C and 50 mTorr ultimate pressure. Tin (II) 2-ethylhexanoate ($Sn(Oct)_2$) was purchased from Sigma-Aldrich and purified through three fractional distillations under dynamic vacuum at 50 mTorr, 145-170 °C. 1,1,1-tris(hydroxymethyl)propane (TMP) was purchased from Sigma-Aldrich, evaporatively recrystallized under nitrogen flow three times from HPLC-grade tetrahydrofuran, washed with cold diethyl ether, and sublimed with a water-cooled cold finger (20 mTorr, 52 °C). Pentaerythritol was purchased from Sigma-Aldrich, recrystallized three times from methanol (7.5 g / 400 mL), and sublimed (165 °C, 25 mTorr). After purification, all the monomers and initiators were stored in a nitrogen glovebox.

Deuterium-labeled chloroform ($CDCl_3$, 99.8% with 0.05% v/v tetramethylsilane (TMS) as reference standard), acetone-d6 ($(CD_3)_2CO$, 99.8%) and water (D_2O, 99.8%) were purchased from Cambridge Isotope Laboratories. Anhydrous ethyl acetate was purchased from Sigma-Aldrich and cannulated from the bottle onto 4-Å molecular sieves in an oven-dried round-bottom flask under an argon atmosphere. Anhydrous toluene was obtained from a JC Meyer solvent drying system and stored over 4-Å molecular sieves in an oven-dried round-bottom flask under an argon atmosphere. 4-Å molecular sieves and were purchased from Sigma-Aldrich. Dichloromethane (DCM), methanol (MeOH), tetrahydrofuran (THF), hexanes, and trifluoroacetic anhydride (TFA) were purchased from Sigma-Aldrich and used without further purification.

Synthesis protocols

Homopolymerization of L-lactide (L23, L78, L500 synthesis). This is the protocol for L23 synthesis. In a nitrogen glovebox, BDM (48.9 mg, 0.354 mmol), L-lactide (9.3479 g, 64.86 mmol), and anhydrous toluene (57 mL) were added to a Schlenk-adapted round-bottom flask equipped with a Teflon-coated stir bar. In the case of L500, BDM initiator was not added. The flask was sealed with a septum. 12.9 μL of 1.014 M $Sn(Oct)_2$ solution (~13 μmol) in toluene was drawn into a 25-μL syringe, and the end of the needle was inserted into a septum to shield the solution from room air. The reaction flask and catalyst were then removed from the glovebox. The flask was connected to an argon Schlenk manifold. The vessel was immersed in a silicone oil bath at 105 °C, and the temperature was equilibrated for 5 minutes. Under positive argon pressure supplied from the Schlenk manifold, the catalyst solution was injected through the flask's septum. Reaction conversion was monitored through ^{1}H-NMR spectroscopy of periodically extracted aliquots. The

conversion v. time data were fitted to a first-order model, and the reaction was stopped by quenching in an ice-water bath at the time predicted to give 70% conversion. The crude product was dissolved in DCM and precipitated into ice-cooled MeOH (3x) and ice-cooled hexanes (1x). Following precipitation, the product was dried under vacuum (50 mTorr) at 80 °C for two days in a drying chamber connected to the Schlenk manifold. Typical yield ~ 93%. ^{1}H-NMR (500 MHz, $CDCl_3$) δ = 7.32 (s, 4 H), 5.28-5.06 (q, 1082 H), 4.35 (quintet, 2 H), 2.64 (d, 2 H), 1.68-1.47 (d, 3273 H).

Synthesis of PγMCL macroinitiators. BnOH, BDM, TMP, and pentaerythritol were used as initiators for the syntheses of 1-, 2-, 3-, and 4-arm star PγMCLs, respectively. The reagent loadings listed here are for $(M22)_1$ but are representative of the other macroinitiator syntheses. In a nitrogen glovebox, γMCL (7.404 g, 57.8 mmol) and BnOH (29.4 µL, 0.283 mmol) were added to a 25-mL round-bottom flask equipped with a Teflon-coated stir bar and a Teflon-sleeved glass stopper. The reaction mixture was heated to 105 °C and equilibrated for 5 min. 12.2 µL of a 0.9335 M solution of $Sn(Oct)_2$ in anhydrous toluene (11.4 µmol) was added to the vessel to start the reaction. Conversion was monitored and modeled similarly as with homopolymer PLLA synthesis; 85% final conversion was targeted. At the estimated stop time, the reaction vessel was removed from the glovebox and immersed in an ice-water bath to quench the reaction. The crude product was dissolved in DCM and precipitated into MeOH (2x) and hexanes (1x) cooled by liquid nitrogen. After the hexanes precipitation, the clear, viscous product was pre-dried by blowing dry nitrogen over the sample for 30 minutes and then dried in a vacuum oven (100 mTorr) for a minimum of 48 hours at 45 °C. The polymer was stored in a jar within an evacuated desiccator. 75-85% yields across reactions. ^{1}H-NMR (500 MHz, $CDCl_3$) δ = 7.43-7.30 (m, 5 H), 5.11 (s, 2 H), 4.24-3.99 (m, 341 H), 3.77-3.62 (m, 2 H), 2.42-2.19 (m, 343 H), 1.75-1.63 (m, 338 H), 1.63-1.53 (m, 215 H), 1.53-1.39 (m, 343 H), 0.97-0.87 (d, 512 H).

Chain extension of PγMCL macroinitiators with L-lactide ($(ML)_n$ synthesis). The reagent loadings listed here are for the chain extension of $(M22)_1$ but are representative of the other macroinitiator syntheses. 4.510 g of $(M22)_1$ PγMCL macroinitiator (0.204 mmol) was charged to an oven-dried 100-mL jar, which was pumped into a nitrogen glovebox. 80 mL of anhydrous toluene was then used to dissolve the PγMCL overnight, aided by a Teflon-coated magnetic stir bar. ~15% w/v of 4-Å molecular sieves was then added to the solution, and the stirring was stopped to avoid damaging the sieves and releasing fragments into the solution. The solution was stored over the sieves for at least one day. 79.6 mL (4.256 g PγMCL, 0.194 mmol) of the macroinitiator stock solution and 18.071 g of L-lactide (125 mmol) were charged to an oven-dried 250-mL round-bottom flask. 31 mL of extra anhydrous toluene was added to bring the initial L-lactide concentration to 1 M. The vessel was then stoppered, heated to 105 °C on a hot plate using a pie-block solid heating mantle, and equilibrated for 5 minutes. 67 µL of a 0.9335 M solution of $Sn(Oct)_2$ in anhydrous toluene (62.6 µmol) was added to the vessel to start the reaction. Conversion was monitored and modeled similarly as with homopolymer PLLA synthesis; 85% final conversion was targeted. At the estimated stop time, the reaction vessel was removed from the

glovebox and immersed in an ice-water bath to quench the reaction. The crude product was dissolved in DCM and precipitated into ice-cooled MeOH (3x) and ice-cooled hexanes (1x). Following precipitation, the product was dried in a vacuum oven (100 mTorr, 90 °C) for two days. Typical yield ~ 93%. ^{1}H-NMR (500 MHz, $CDCl_3$) δ = 7.40-7.30 (m, 5 H), 5.28-5.06 (q, 1176 H), 4.35 (quintet, 1 H), 4.24-3.99 (m, 340 H), 2.67 (d, 1 H), 2.42-2.19 (m, 340 H), 1.75-1.39 (m, 4639 H), 0.97-0.87 (d, 512 H).

Characterization

<u>NMR spectroscopy</u>. ^{1}H-NMR and ^{13}C-NMR spectra were recorded on a Bruker Avance III HD 500 MHz spectrometer. Chemical shifts are reported in δ units, expressed in ppm using the TMS signal (0.00 ppm) as an internal standard for $CDCl_3$ and the $CD_3C(O)CD_2H$ (2.05 ppm) and CD_2HOD (3.31 ppm) signals as internal standards for acetone-d6 and methanol-d4, respectively.

<u>*Differential scanning calorimetry (DSC)*</u>. Experiments were performed using a TA Instruments Discovery DSC. Film specimens ranging in mass from 2-7 mg were hermetically sealed in aluminum Tzero pans in open air unless otherwise noted. Scans were conducted under a nitrogen atmosphere. When samples were "melted," they were heated 15-17 °C above the observed T_m (temperature of maximum endothermic heat flow during melting) and held isothermally for 3-5 minutes.

Dynamic experiments. 10 °C min^{-1} ramp rate. (1) Melt sample; (2) cool to -90 °C and equilibrate for 10 minutes; (3) melt sample. In TRIOS analysis software, T_g values were determined from a midpoint half-height analysis, and phase transition enthalpies (ΔH) were calculated from numerical integration of heat flow v. T traces using local baselines. Crystallinity (X_c) was calculated using the following equation:

$$X_c = \frac{(\Delta H)_{net}}{w_{PLLA}(\Delta H)^0_{m,PLLA}} = \frac{(\Delta H)_m - (\Delta H)_{pmc} - (\Delta H)_{cc}}{w_{PLLA}(\Delta H)^0_{m,PLLA}} \quad (S1)$$

where $(\Delta H)_{net}$ is the net endothermic enthalpy determined by integrating across the cold crystallization (cc), pre-melting crystallization (pmc), and melting peaks (m) in the heating trace; w_{PLLA} is the total weight fraction of PLLA in the material; and $(\Delta H)^0_{m,PLLA}$ = 93.7 J g^{-1} is the melting enthalpy of a pure, infinite PLLA crystal.[1]

Isothermal experiments. (1) Melt sample; (2) cool at 80 °C min^{-1} to the crystallization temperature (T_c; either 100 or 130 °C); (3) hold isothermally until the entire crystallization exotherm has been captured; (4) melt sample. The overall rate of crystallization was quantified by the inverse of the exotherm peak time, t_p^{-1}, where t_p is the time elapsed after cooling to T_c at which the largest exothermic heat flow rate occurs. As recommended by Lorentzo *et al.*,[2] we chose this approach instead of Avrami analysis because we were unable to collect steady heat flow baselines prior to the onset of crystallization.

Aging study – varied aging times. Samples were dried in a vacuum oven overnight prior to being hermetically sealed in the Tzero pans. (1) Melt sample; (2) cool at 80 °C min^{-1} to 0 °C and equilibrate for 10 minutes; (3) heat at 20 °C min^{-1} to aging temperature and hold isothermally for t_a hours; (4) cool at 20 °C min^{-1} to 0 °C and equilibrate for 10 minutes; (5) melt sample, heating at 10 °C min^{-1}. Perform this protocol for aging times t_a of 0, 1, 2, 3.5, 6, and 10 hours. The aging temperature was set at 45 °C to maximize the enthalpy relaxation as determined from trial experiments on $(M24\text{-}L81)_3^*$ (**Figure S46**). The enthalpy recovery $(\Delta H)_{rec}$ was calculated by integrating the heat capacity curves from 50

to 75 °C and subtracting the corresponding integral for t_a = 0 hr following Petrie's method[3] and after aligning the traces at T = 75 °C to minimize instrumental drift errors as described by Koh and Simon.[4]

In aging studies of block polymers, normalizing the recovered enthalpy to the content of the aging block (PLLA in this case) is essential. One can simply divide the heat flow traces by the weight fraction of the aging block, which is useful for studies of a single material.[5] However, the limitations on the accuracy of block composition determination make this ill-suited for comparisons of multiple block polymers with different compositions, like the $(ML)_n$ star-blocks studied here with PLLA weight fractions scattered closely around 0.8. Instead, we normalized the observed $(\Delta H)_{rec}$ by the theoretical enthalpy recovery at equilibrium, or infinite aging time, $(\Delta H)_{rec,\infty}$:

$$(\Delta H)_{rec,\infty} = \int_{T_a}^{T_{f,lim,0}} \Delta C_p(T)\, dT \quad (S2)$$

where T_a is the annealing temperature; $T_{f,lim,0}$ is the limiting fictive temperature determined from the t_a = 0 hr heating trace; and $\Delta C_p(T) = C_{p,l}(T) - C_{p,g}(T)$ is the temperature-dependent difference between the liquid and glass heat capacities above and below $T_{g,\mathrm{PLLA}}$, respectively.[4] The fictive temperature (T_f) describes the temperature at which a glass has the same enthalpy (or any evolving state function) as its liquid if the liquid's heat capacity were extrapolated into the glassy temperature regime. It encodes structural stability; a higher T_f describes a less stable, more disordered, high-free volume/high-enthalpy state, while a lower T_f describes a more stable, more liquid-like, lower-enthalpy state. Conceptually, the limiting fictive temperature ($T_{f,lim}$) is the temperature during the *cooling* trace at which segmental motions that enable enthalpy relaxation are quenched on the experimental timescale – *i.e.*, the cooling rate. It is a "starting point" for structural relaxation: during aging, T_f decreases from its initial value $T_{f,lim}$ toward T_a. Once T_f = T_a, the structure has released all the enthalpy allowed by $\Delta C_p(T_a)$, and the material is at equilibrium. Operationally, it is obtained from *heating* traces as shown graphically in **Figure S54**, whereby the net excess enthalpy curve (running integral of $C_p(T) - C_{p,g}(T)$) is extrapolated back along a fit in its liquid regime above $T_{g,\mathrm{PLLA}}$ to self-intersect at $T_{f,lim}$. We estimated $(\Delta H)_{rec,\infty}$ in this way and used it to calculate a "fractional physical age," the observed $(\Delta H)_{rec}$ at a given t_a normalized by $(\Delta H)_{rec,\infty}$:

$$\text{fractional physical age at } t_a = \frac{(\Delta H)_{rec}(t = t_a)}{(\Delta H)_{rec,\infty}} \quad (S3)$$

We calculated $\Delta C_p(T)$ by directly fitting linear functions to the glassy and liquid regimes of $C_p(T)$ for the t_a = 0 hr heating trace in each experiment. We preferred this method because any error introduced by sample mass uncertainty scales all heat flow (and therefore C_p) traces identically in the same experiment, and the area normalization approach described in Equation S3 eliminates heat flow scaling errors. However, $\Delta C_p(T)$ is most

properly evaluated by direct measurement of $C_p(T)$ using quasi-isothermal DSC techniques as exemplified by Koh and Simon.[4] To ensure our conclusions about physical aging were not influenced by our method for determining $\Delta C_p(T)$, we also conducted quasi-isothermal modulated DSC experiments to directly measure $C_p(T)$ and fitted glassy and liquid regions to those datasets to determine $\Delta C_p(T)$. (Parameters: 0.50 °C amplitude, 120 s period, modulate for 10 min at each temperature, incrementing temperature by 2.5 °C starting at 15 °C.) **Figure S49** displays the $C_p(T)$ traces and the fits from this method. However, cold crystallization of the PLLA occurred above $T_{g,\ PLLA}$, limiting the range of points available for the liquid regime fit. Furthermore, the difference in mass between samples used for $C_p(T)$ determination and the isothermal aging study introduces an additional error source. Nonetheless, **Figure S50** shows qualitatively the same aging behavior when $\Delta C_p(T)$ was evaluated using the second method.

In isothermal aging studies, the subcooling $T_{g,PLLA} - T_a$ influences the aging rate. Noting the slight spread in $T_{g,\ PLLA}$ values across the $(ML)_n$ star-blocks, we conducted the isothermal aging at fixed subcooling below $T_{g,PLLA}$ for L78, $(M8\text{-}L33)_3$, and $(M24\text{-}L86)_3$, still finding that the star-blocks attained higher physical age than L78 (**Figure S51**).

Aging study – varied cooling rate. Samples were dried in a vacuum oven overnight prior to being hermetically sealed in the Tzero pans. (1) Melt sample; (2) cool at 80 °C min^{-1} to 70 °C and equilibrate for 10 minutes; (3) cool at q_1 °C min^{-1} to 0 °C and equilibrate for 10 minutes; (4) melt sample, heating at 10 °C min^{-1}. Perform this protocol for cooling rates q_1 of 10, 5, 2.5, 1, 0.5, 0.25, and 0.1 °C min^{-1}. The enthalpy overshoots were measured during Step 4 as described above. For activation energy determinations, $T_{f,\ lim}$ was calculated for the different cooling rates as graphically displayed in **Figure S54**.

The TNM model is defined by the following two equations:[6]

$$T_{f,n} = T_{f,0} + \sum_{i=1}^{n} (\Delta T)_i \left[1 - \exp\left(-\left(\sum_{j=i}^{n} \frac{(\Delta t)_j}{\tau_j} \right)^{\beta} \right) \right] \quad (S4)$$

$$\tau_j = \tau_\infty \exp\left[\left(\frac{\Delta h^*}{R} \right) \left(\frac{x}{T_j} + \frac{1-x}{T_{f,j}} \right) \right] \quad (S5)$$

where $T_{f,n}$ is the fictive temperature at timestep n; ΔT is the change in temperature associated with each timestep, calculated as $q_1 \cdot \Delta t$; Δt is the timestep duration; τ_j are the time constants associated with the first-order accumulation of T_f changes; β is the stretching exponent encoding the nonexponentiality of the aging kinetics; Δh^* is the aging activation energy; x is the nonlinearity parameter encoding the influence of structural stability on the aging rate; and τ_∞ is the fictitious relaxation time at infinite temperature. To fit our fixed-q_1 datasets, we normalized the raw heat capacities from the reheating curves:

$$C_{p,N}(T) = \frac{C_p(T) - C_{p,g}(T)}{C_{p,l}(T) - C_{p,g}(T)} = \frac{dT_f}{dT} \quad (S6)$$

where $C_{p,g}(T)$ and $C_{p,l}(T)$ were determined by fits to the trace in linear regimes below and above $T_{g,\text{ PLLA}}$, respectively. We then evaluated the TNM model using the thermal protocol described above, using a temperature increment of 0.1 °C for the dynamic stages and 500 logarithmically spaced timesteps from 0.001 min to 10 min for the isothermal hold at 0 °C. The resulting T_f trace on reheating was then numerically differentiated to produce $C_{p,N}(T)$. All computations and fit optimizations were performed in the Jupyterlab Python environment using the `minimize` function from the `scipy.optimize` library. Due to the large computational burden, each cooling rate trace was optimized separately, producing a set of four parameters $p = [\tau_\infty, \Delta h^*, x, \beta]$ for each trace, along with their standard errors (σ) estimated using the diagonal elements of the inverse Hessian matrix ($\boldsymbol{H}^{-1}$) output from `minimize`:

$$\sigma_i = \sqrt{\left(\frac{SSR}{df}\right)\boldsymbol{H}_{ii}^{-1}} \quad (S7)$$

Then, the average parameter values ($\langle p_i \rangle$) and their standard errors ($\langle \sigma_{p,i} \rangle$) were estimated by inverse-variance weighting:

$$\langle p_i \rangle = \frac{\sum_{q_1} \frac{p_{i,q_1}}{\sigma_{p,i,q_1}^2}}{\sum_{q_1} \frac{1}{\sigma_{p,i,q_1}^2}}; \ \langle \sigma_{p,i} \rangle = \sqrt{\frac{1}{\sum_{q_1} \frac{1}{\sigma_{p,i,q_1}^2}}} \quad (S8)$$

These are the values and standard errors reported in **Figure 4d** and **Figure S57**.

Thermogravimetric analysis (TGA). Experiments were performed on melt-pressed $(ML)_n$ films using a TA Instruments Q500 under nitrogen atmosphere with a heating rate of 10 °C min^{-1}.

Size exclusion chromatography (SEC). THF-SEC was performed using 0.5-2 mg mL^{-1} solutions of polymer in THF stabilized by 0.5 wt % BHT (Sigma Aldrich) on an EcoSEC SEC system (Elite 8240GPC series, Tosoh Bioscience, LLC) fitted with a refractive index detector and two Tosoh TSKgel GMHHR-M columns. Chloroform-SEC was performed on L496 using a 1 mg mL^{-1-} solution of polymer in HPLC-grade chloroform (Fisher Scientific) on the same device/column listed above that was, at the time, equipped with a LenS3 multi-angle laser light scattering (MALLS) detector (Tosoh Bioscience, LLC).

Film processing and tensile testing. Before melt-processing, all materials were dried under vacuum overnight at 50 °C. Dried polymer precipitates were melt-pressed into films at 190 °C using a Genesis Series hydraulic lab press (Wabash, IN) between Teflon sheets (American Durafilm Co., Inc.) sandwiched between stainless steel plates. The "rapidly quenched" thermal history involved transferring the molten film directly to a secondary press circulated with cooling water, and

quenching was completed in <1 minute. The "melt crystallized" thermal history involved cooling the molten film to 100 °C using cooling water (~3 min), holding for 5 or 10 minutes after the first platen temperature read 100 °C, and then transferring the film to the cold press for rapid quenching. The film mold thickness was 250 μm. All samples were aged for 3 or 80 days at room temperature prior to tensile testing unless otherwise noted. The films were cut into dumbbell-shaped tensile bars using a specimen cutter (Dumbbell Co., Ltd. SDL200, equipped with an SDMK-1000 dumbbell cutter) in accordance with ASTM D1708. Tensile bars were elongated at room temperature using a tensile tester (Shimadzu Autograph AGS-X) operated at 1 mm min^{-1} crosshead speed in accordance with ASTM D1708. Selected tensile specimens were video recorded (iPhone 13 Pro Max, time lapse capture) to monitor necking and whitening processes. Young's modulus (E), yield stress (σ_y), elongation at break (ε_B), stress at break (σ_B), tensile toughness, and strain-hardening modulus (G_R) were calculated from the stress strain data. Error bars denoted by "±" show 95% confidence intervals calculated using a student's t statistic.

Atomic force microscopy (AFM). Samples were cryo-microtomed with a glass knife using a Leica EM UC6 ultramicrotome operating at −120 °C (model FC-S Cryo attachments) to create a freshly cut surface. Then, imaging specimens were cut with a thickness of 300 nm using a Diatome diamond knife and transferred *via* eyelash to a silicon wafer. AFM was conducted in AC mode (Bruker Nanoscope V Multimode 8 open-loop system, Santa Barbara, CA) whereby the Z piezoscanner ("height") is reactively displaced by the control electronics under feedback to maintain a constant engaged cantilever amplitude A (reduced from its free value). N-type Si cantilevers of nominal stiffness constant 7.8 N/m (AppNano, ACTA, ~145 kHz resonant frequency, <10 nm tip diameter) were operated at ~0.2 kHz below fundamental flexural resonance and at relative amplitude setpoints (operational amplitude relative to free-oscillation resonant amplitude, A/A_0) of 0.5-0.7 to favor stabilization in the net repulsive regime.[3]

X-ray scattering. Synchrotron X-ray scattering data were acquired from the 11-BM CMS beamline at the National Synchrotron Light Source II (NSLS-II; Brookhaven, NY) using a photon wavelength of λ = 0.7293 Å. All images were collected from two-second exposures. The 2D detector images were either directly analyzed or azimuthally integrated to produce 1D patterns of intensity $I(q)$ versus scattering wavevector magnitude $q = |\mathbf{q}| = 4\pi\lambda^{-1} \sin(\theta/2)$. Static room temperature images were collected from film samples mounted upright on Kapton tape. For variable-temperature experiments, ~4 x 4 mm-sized film pieces were stacked in aluminum Tzero DSC pans, which were then hermetically sealed in a nitrogen glovebox to prevent hydrolytic degradation in the melt. In staged cooling experiments, samples were annealed in the melt at 210 °C for 10 minutes before cooling in 20-°C increments, with 5 minutes of equilibration held before each set of isothermal scans. *In-situ* tensile X-ray specimens were mounted onto a Linkam tensile stage with 60-grit sandpaper sandwiching the gripped region. The specimens were elongated at 1 mm min^{-1}, and two-second exposures were taken every four seconds during elongation. All images and patterns were corrected by subtracting the intensities from appropriate blanks (dark field images for static and *in-situ* tensile samples and blank DSC pans for variable-temperature scans).

By extracting the principal SAXS peak positions (q^*), we determined the PγMCL domain spacings (d) according to

$$d = \frac{2\pi}{q^*} \quad (1)$$

To estimate the PγMCL-PLLA interfacial width in as-processed specimens, we analyzed Porod scattering in the room temperature films. Specifically, we employed Yu *et al.*'s procedure of fitting high-q regions of the room temperature SAXS patterns with a power law model, subtracting the fitted background term, and linearizing the resultant pattern ($I_{\mathrm{corr}}(q)$) assuming Porod scattering convolved with a Gaussian smoothing term to account for finite interfacial width:[7]

$$I_{corr}(q) \sim q^{-4} \exp(-\sigma^2 q^2) \quad (1)$$

such that a plot of $\ln(q^4 I_{\mathrm{corr}})$ v. q^2 produces a linear plot with slope of $-\sigma^2$, with the interfacial width related to σ by $w_{\mathrm{I}} = (2\pi)^{1/2}\sigma$. The linearized patterns are shown in **Figure S23**.

The SAXS invariant (Q) was calculated from a 15° wide azimuthal slice centered on the meridional axis as

$$Q \equiv \int_0^\infty q^2\, I(q)\, dq \quad (2)$$

The relative invariant was obtained by dividing by the value calculated at 0% strain.

Supporting figures

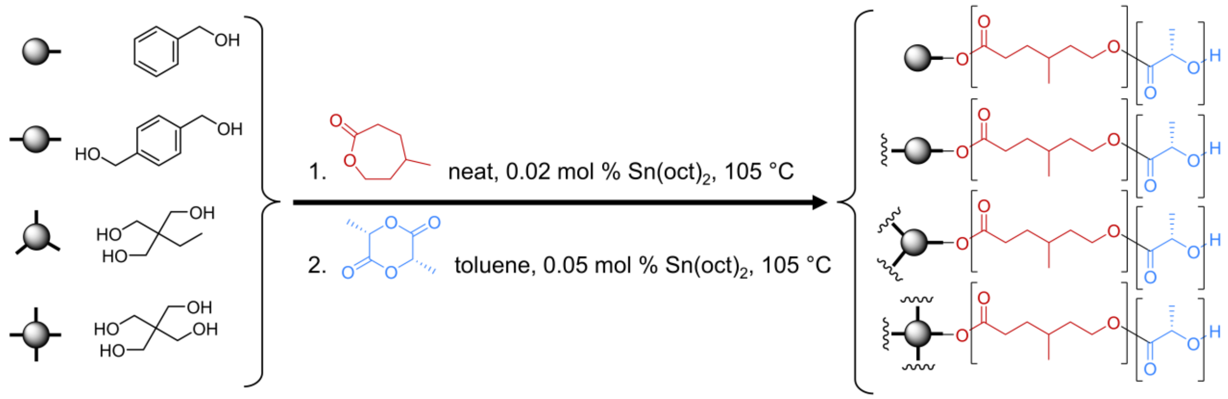

Scheme S1. $(ML)_n$ star-blocks were synthesized through two-step ring-opening transesterification polymerization, with *n* controlled by the functionality of the small molecule alcohol initiator.

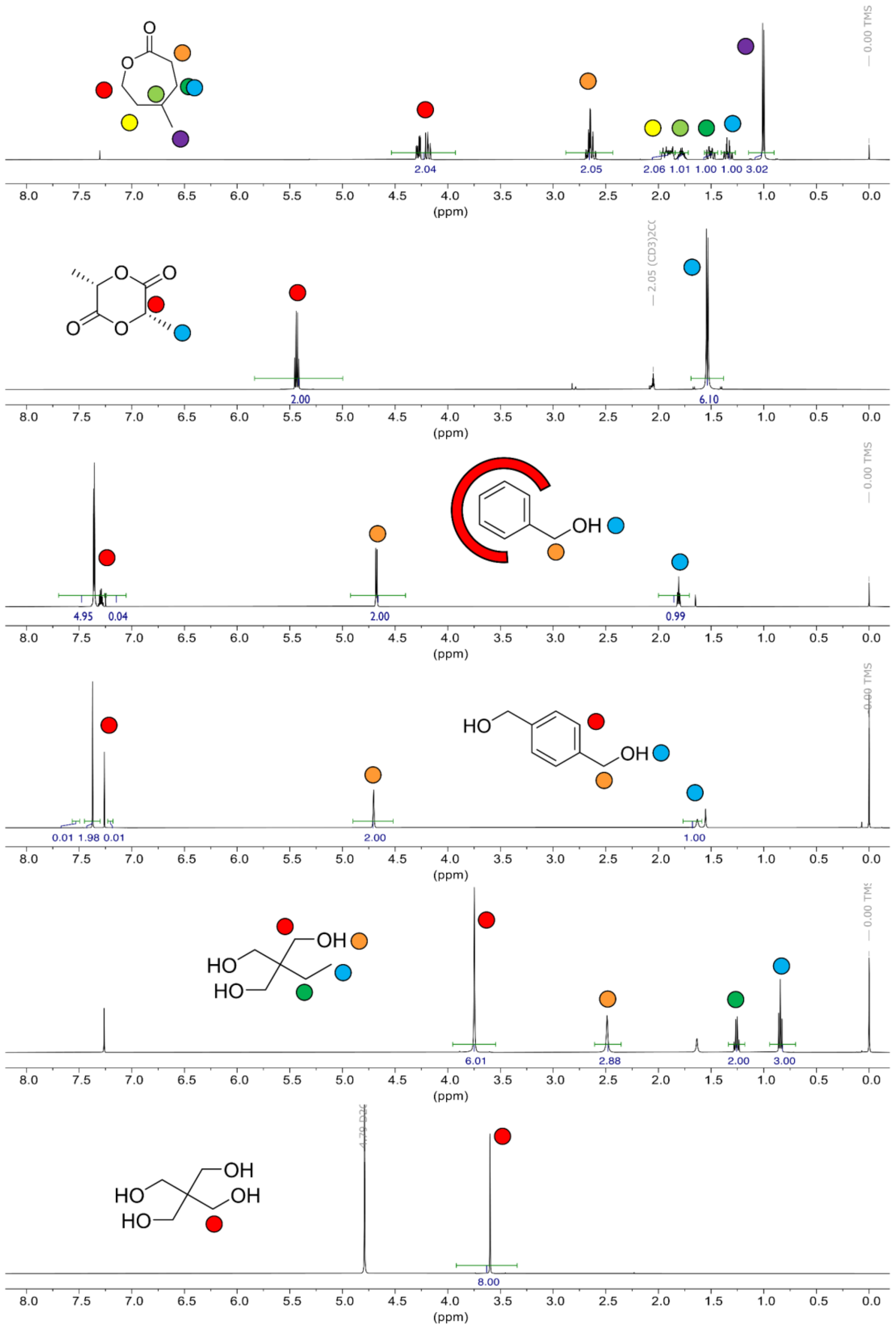

Figure S1. ^{1}H-NMR spectra of small-molecule monomers and initiators after purification.

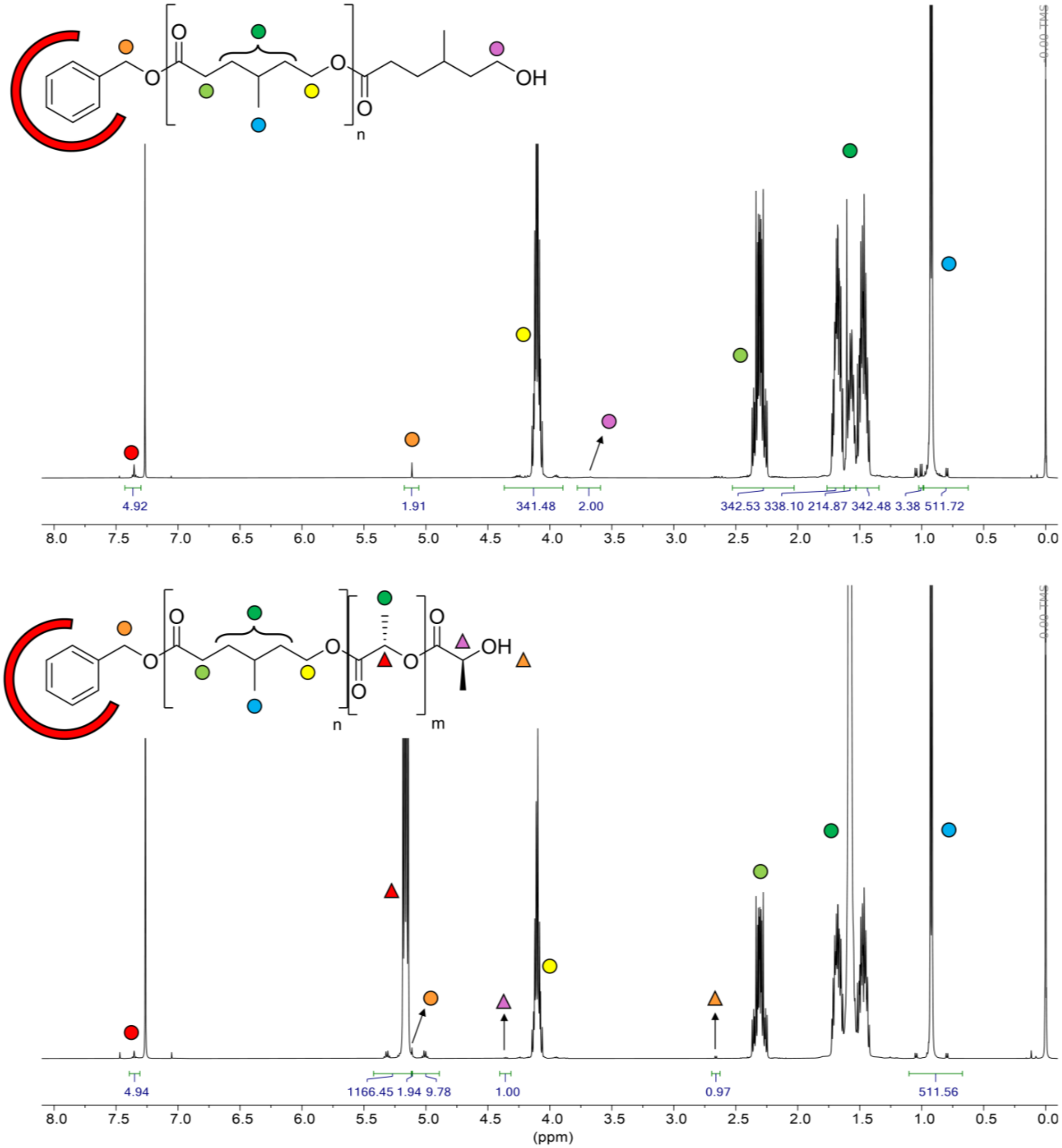

Figure S2. ^{1}H-NMR spectra of $(M22)_1$ (top) and $(M22\text{-}L85)_1$ (bottom).

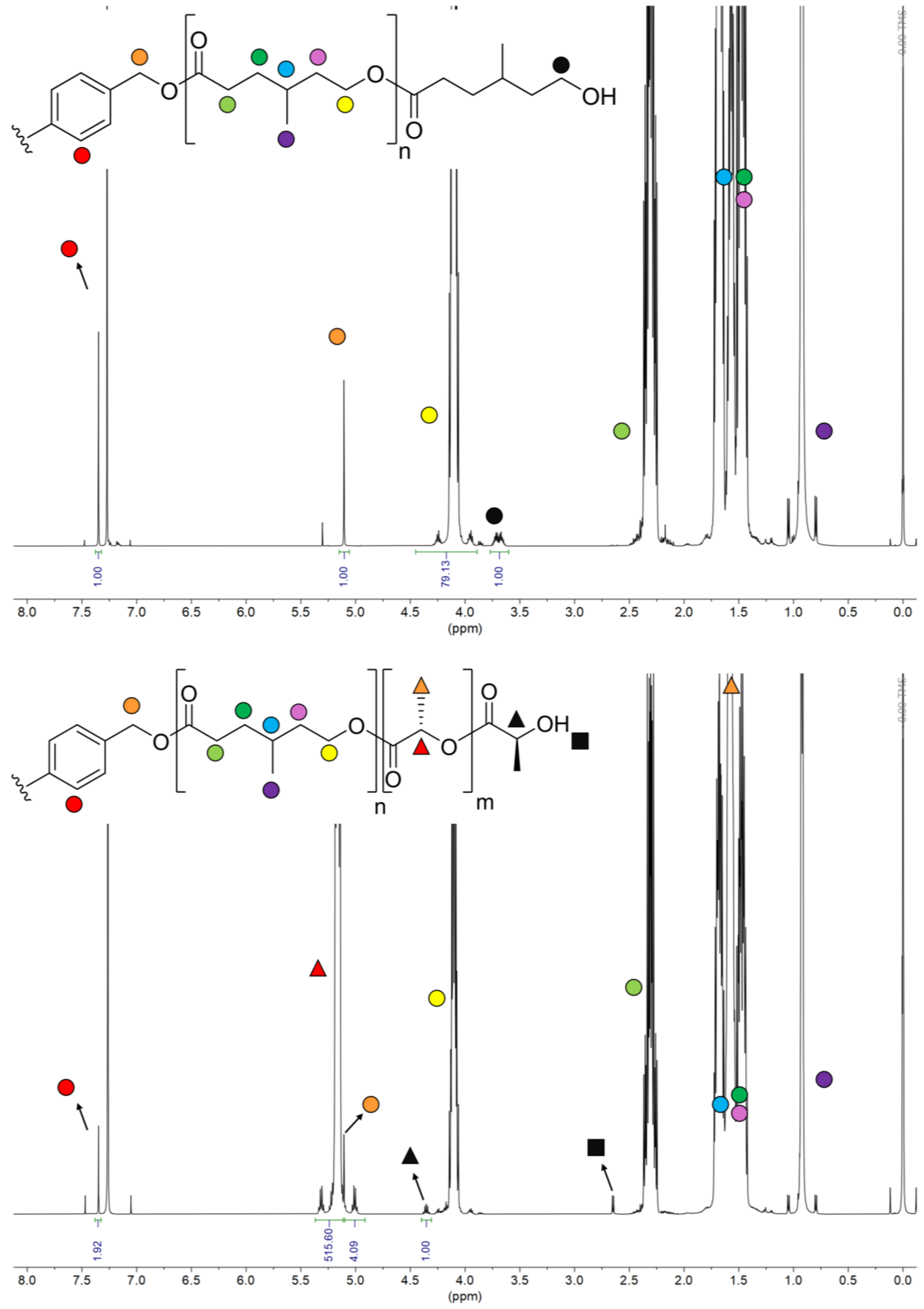

Figure S3. ^{1}H-NMR spectra of $(M10)_2$ (top) and $(M10\text{-}L38)_2$ (bottom).

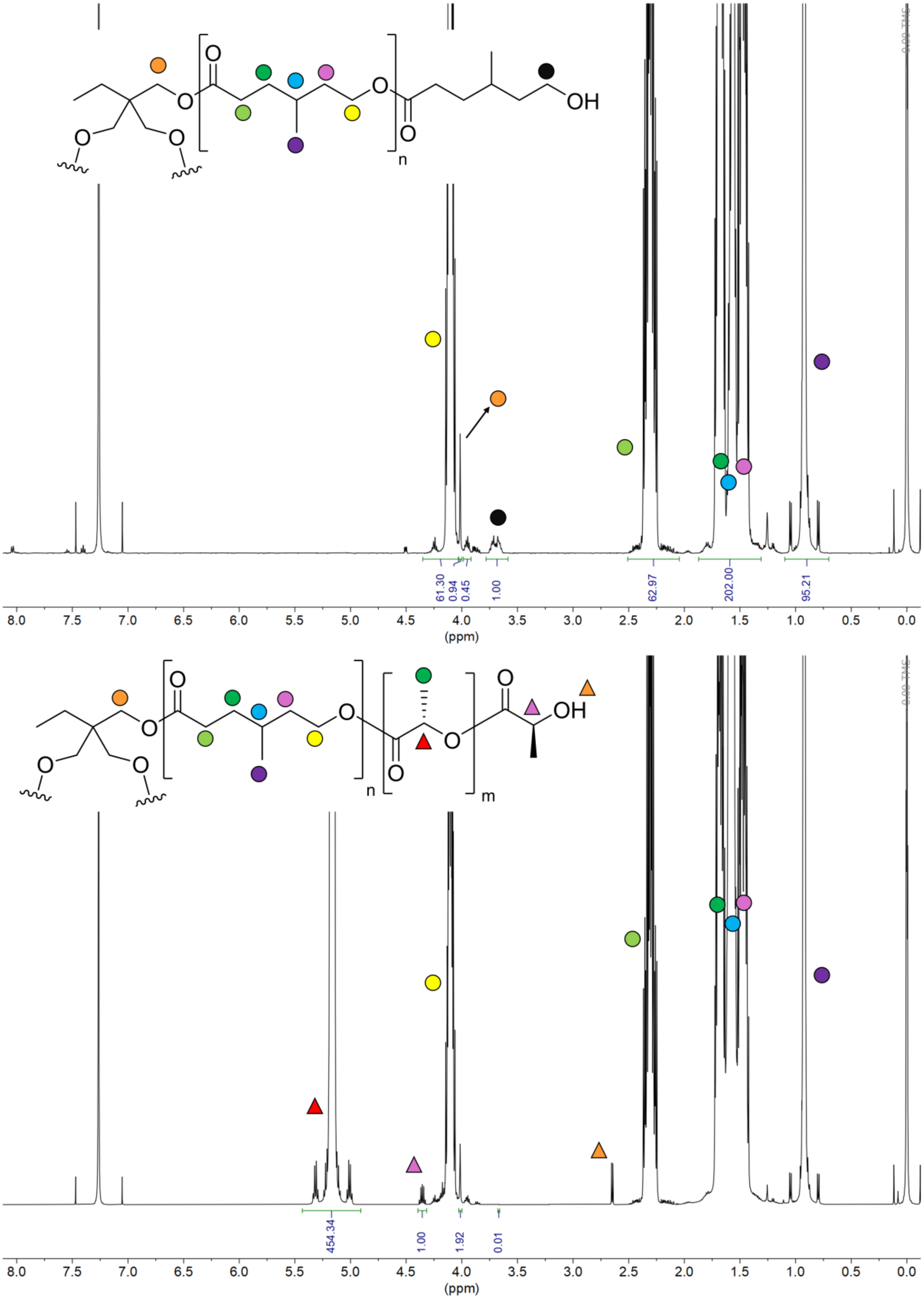

Figure S4. ^{1}H-NMR spectra of $(M8)_3$ (top) and $(M8\text{-}L33)_3$ (bottom).

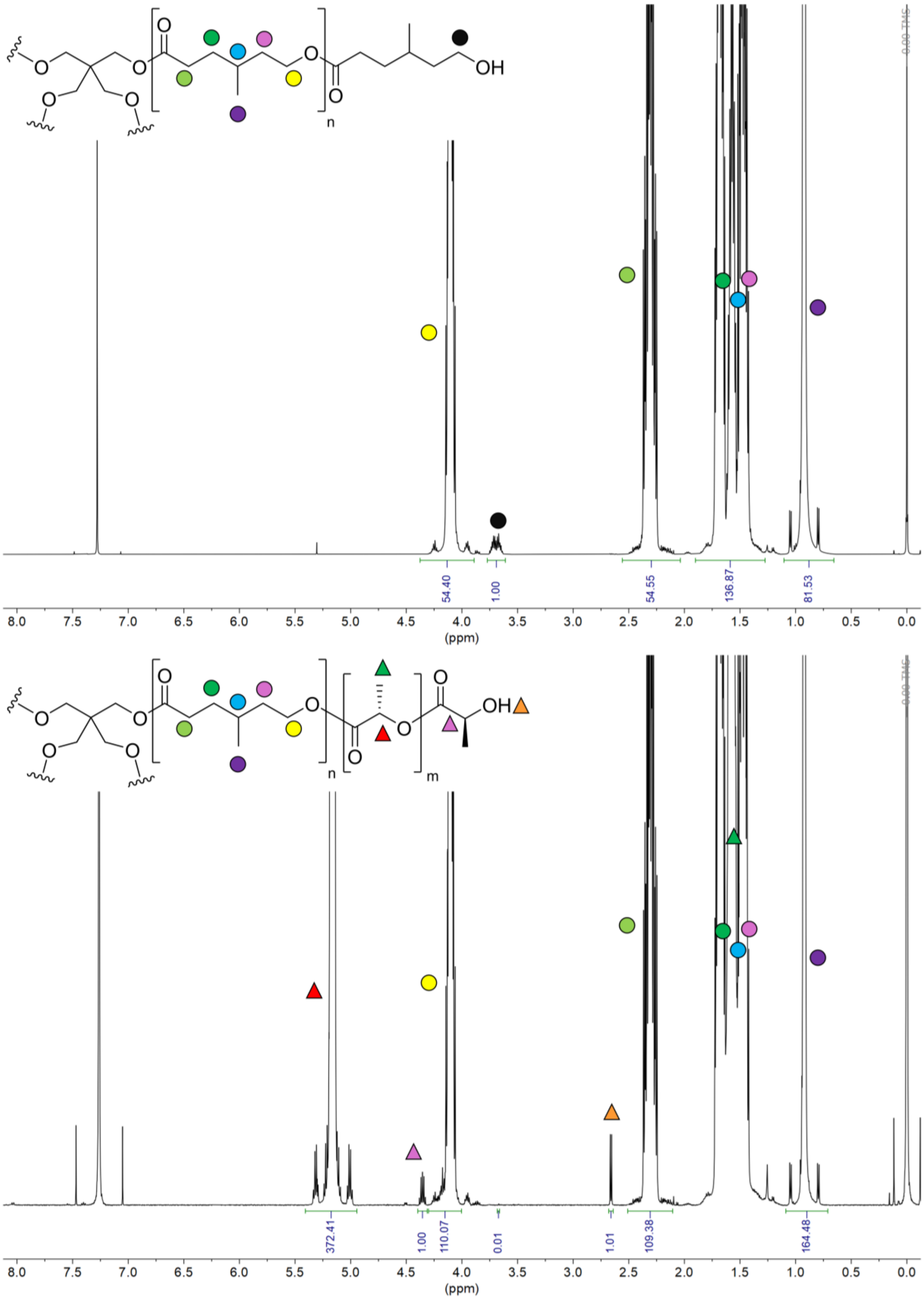

Figure S5. ^{1}H-NMR spectra of $(M7)_4$ (top) and $(M7\text{-}L27)_4$ (bottom)

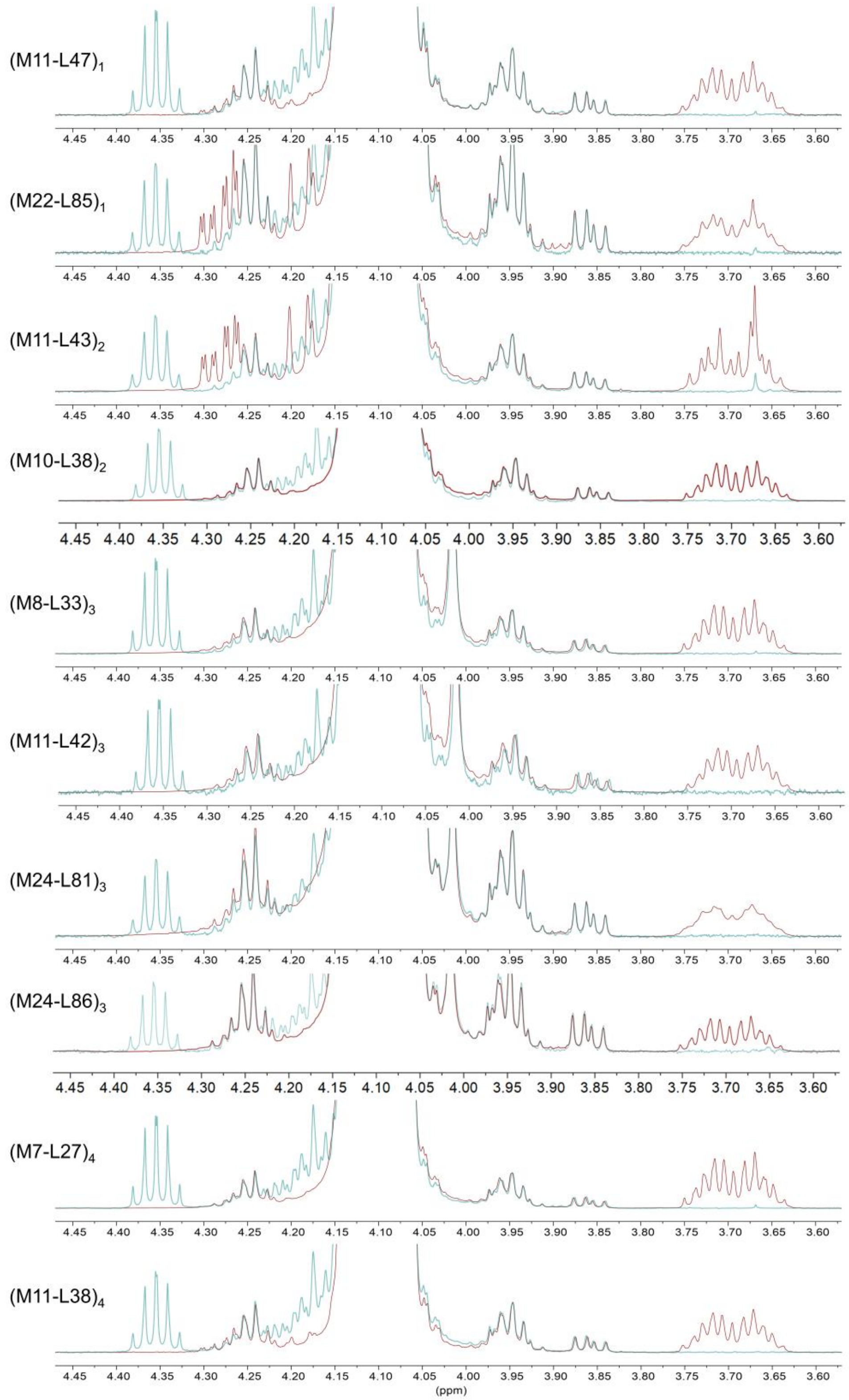

Figure S6. Overlaid ^{1}H-NMR spectra of PγMCL macroinitiators (maroon) and $(ML)_n$ star-blocks (teal) showing complete disappearance of the PγMCL macroinitiator -C**H**$_2$OH end-group methylene protons (3.77-3.62 ppm) and replacement with PLLA arm -C**H**(CH$_3$)OH end-group methine protons (4.39-4.32 ppm)

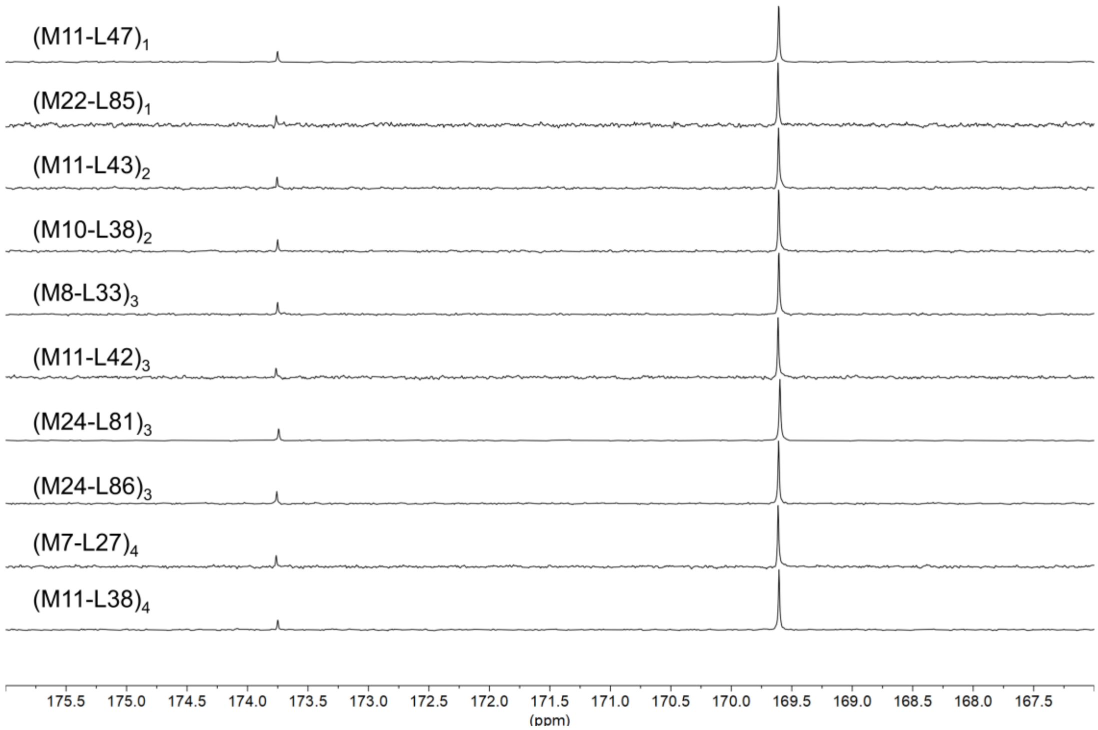

Figure S7. Carbonyl region of ^{13}C-NMR spectra of all $(ML)_n$ block polymers. Only two carbonyl resonances appear: one at 173.75 ppm for the PγMCL block, and one at 169.6 ppm for the PLLA block. The consistency in the relative intensities of these peaks also qualitatively demonstrates compositional consistency.

Pseudo-first order kinetics were observed for the BnOH (n = 1) and BDM-initiated syntheses (n = 2) (**Figure S8**). We observed induction periods in conversion when initiating PγMCL core blocks with 1,1,1-trimethylolpropane (TMP; n = 3) and pentaerythritol (PE; n = 4), which created some difficulty in controlling PγMCL arm length. In these cases, we increased the temperature or catalyst loading partway through the reaction. **Table S1** below lists the relevant information for the PγMCL macroinitiator syntheses. After this change in conditions, the kinetics accelerated considerably, and the conversion could not be measured quickly enough (remove aliquots from glovebox, prepare ^{1}H-NMR samples, acquire spectra for 5 min each) to stop the reactions at the correct times for all reactions. $(M8)_3$ and $(M7)_4$ macroinitiators consequently exceeded their molar mass targets. In these cases, maintaining w_{PLLA} created some M_{tot} spread in the "fixed M_{tot}" series: M_{tot} ranges from 96 kg mol^{-1} for $(M10\text{-}L38)_2$ to 136 kg mol^{-1} for $(M7\text{-}L27)_4$. Concerned about incomplete initiation in macroinitiator synthesis for n = 3 and n = 4, we transesterified TMP and PE with trifluoroacetic anhydride, as well as their downstream $(M)_n$ and $(ML)_n$ species, to look for uninitiated core hydroxyls. This was not possible for n = 3, as the methylene proton resonances from TMP and PγMCL end-groups overlapped after trifluoroacetylation (**Figure S9**). For n = 4, these resonances did not overlap, revealing some uninitiated PE hydroxyl groups after PγMCL core synthesis for $(M7)_4$ but not for $(M11)_4$ (**Figure S10**). Assuming that only one hydroxyl group was uninitiated per PE molecule, we used resonance integral ratios to calculate a content of 8 mol % (11 wt %) of $L_1(ML_1)_3$-type miktoarm star in $(M7\text{-}L27)_4$, as the trifluoroacetylated PE hydroxyl groups were absent in the star-block spectrum, indicating PLLA initiation by the PE hydroxyl groups intact after the first step.

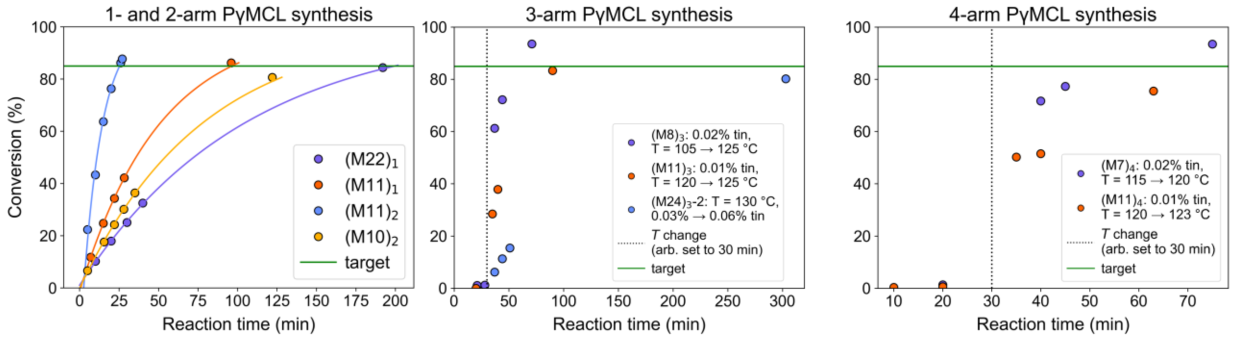

Figure S8. Reaction kinetics (conversion v. time) observed for PγMCL macroinitiator syntheses. In the first panel, the first-order fits are shown as solid lines. In the second two panels, the time of the temperature change was arbitrarily set to 30 minutes for visual convenience. For all these reactions, the conversion remained ~0% until the temperature or catalyst loading was increased.

Table S1. $[M]_0/[I]_0$ ratios, catalyst loadings, starting and adjusted temperatures, and conversion fidelity in PγMCL macroinitiator syntheses. Final conversion (p_f) and arm molar mass were determined with ^{1}H-NMR spectroscopy.

Product	$[M]_0/[I]_0$	$[cat]/[M]_0$ (mol %)	T_i, T_f (°C)	p_f	$M_{n, arm, targ}$ (kg mol^{-1})	$M_{n, arm, obs}$ (kg mol^{-1})
$(M11)_1$	100	0.02	105	0.862	11	11.48
$(M22)_1$	204	0.02	105	0.844	22	22.0
$(M11)_2$	221	0.10	105	0.877	10	11.0
$(M10)_2$	195	0.03	105	0.807	10	10.2
$(M8)_3$	201	0.02	105, 125	0.935	7.3	8.0
$(M11)_3$	301	0.01	120, 125	0.833	11	10.8
$(M24)_3$ – 1 for $(M24\text{-}L81)_3$	737	0.07	130	0.855	22	24.0
$(M24)_3$ – 2 for $(M24\text{-}L86)_3$	659	0.03, 0.06[a]	125, 130	0.802	24	24.4
$(M7)_4$	201	0.02	115, 120	0.935	5.5	7.0
$(M11)_4$	403	0.01	120, 123	0.755[b]	11	10.9

[a] Here, the catalyst loading was doubled after the temperature was increased.

[b] Balance fluctuations were suspected to have caused an over-loading of initiator, so the reaction was stopped at 76% conversion rather than 85% to better target the 11-kg mol^{-1} arm.

Kinetic non-idealities have been observed using multifunctional initiators for the ROTEP of lactide using $Sn(Oct)_2$ catalyst. Kim *et al*. observed incomplete initiation of all pentaerythritol hydroxyl groups at low $[M]_0/[I]_0$ (< 32) at 130 °C, while Puchkov *et al*. showed that increasing $[M]_0/[I]_0$ from 10 to 100 led to slower reaction rates for TMP and pentaerythritol initiators at 140 °C.[8,9] They also noted that despite overall pseudo-first order kinetics, there was an induction period over which the initiator hydroxyl groups gradually transesterified with monomer. At 160 °C, glycerol-initiated ROTEP directly shows a conversion induction period for $[M]_0/[I]_0 \geq 2000$.[10] The authors suspected that the steric hindrance of initial monomers restricted access to the remaining uninitiated hydroxyl groups. L-lactide is a six-membered ring, while γMCL is a seven-membered ring, and so the partially initiated core molecules with one or two γMCL monomers added may be sterically bulkier, further increasing the activation energy associated with initiating from remaining hydroxyl groups. This effect, coupled with the lower reaction temperatures used here, likely accentuated the induction period.

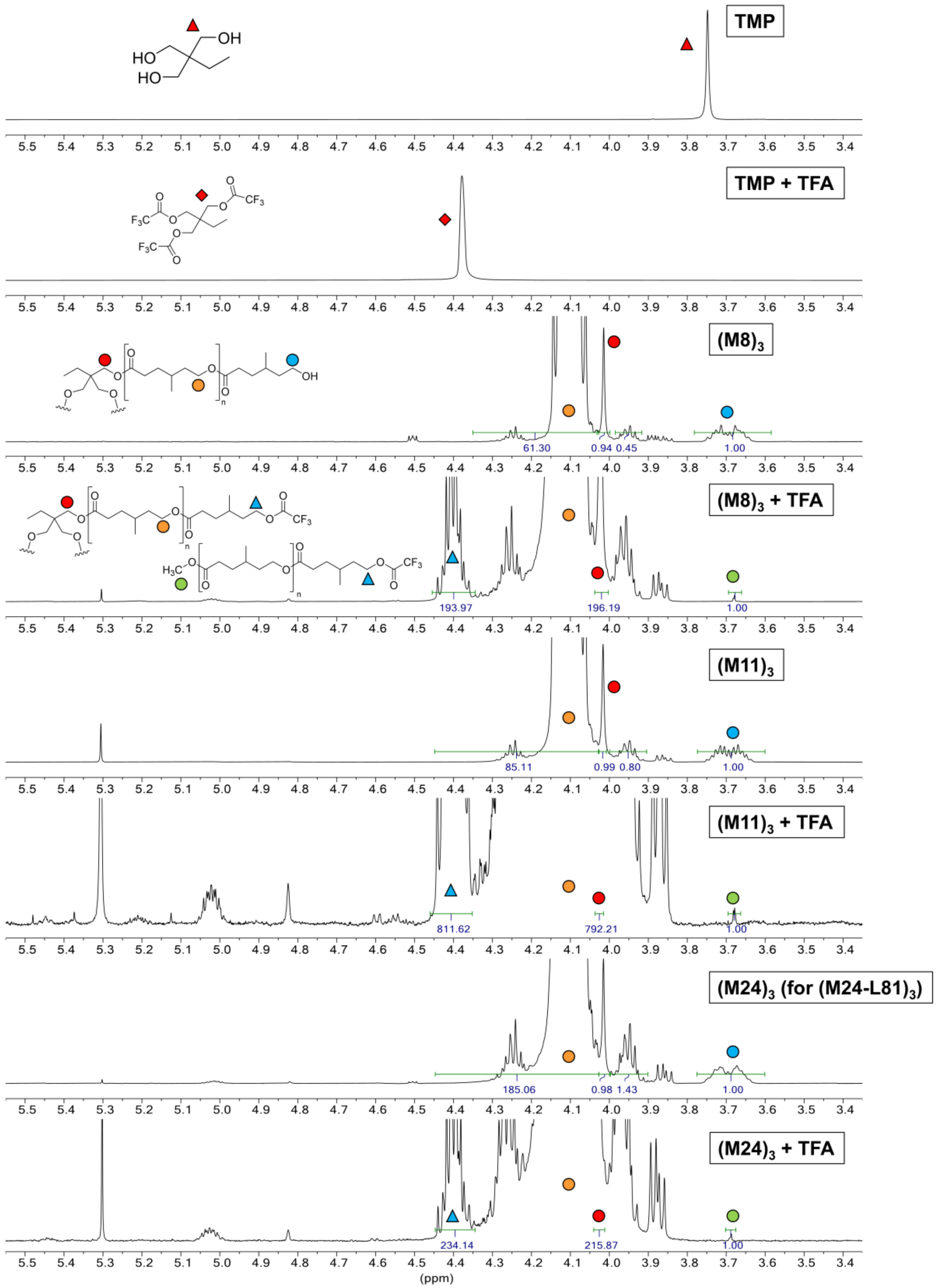

Figure S9. ^{1}H-NMR studies of trifunctional initiator and macroinitiator molecules transesterified with trifluoroacetic anhydride (TFA).

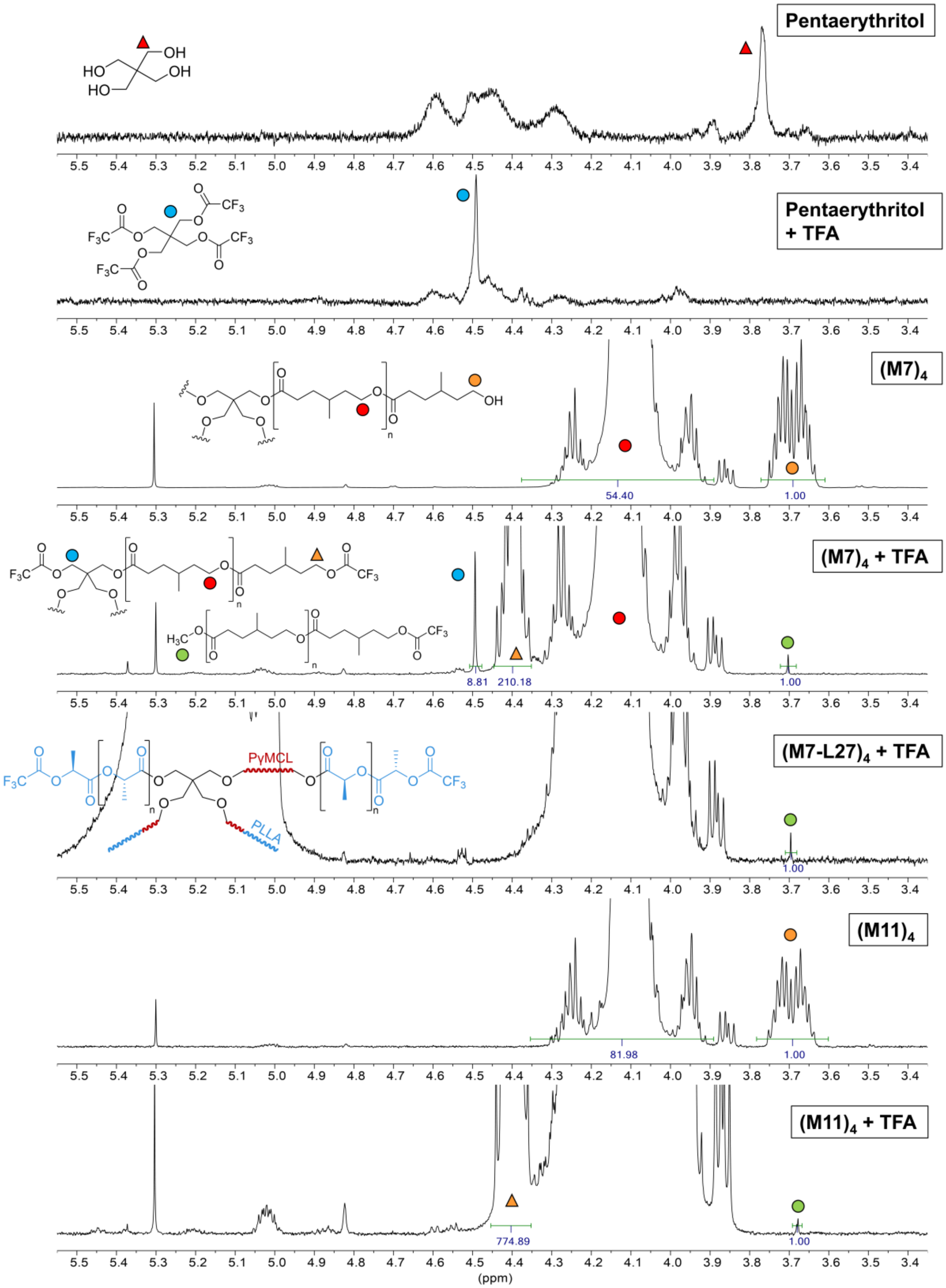

Figure S10. ^{1}H-NMR studies of tetrafunctional initiator and macroinitiator molecules transesterified with trifluoroacetic anhydride (TFA).

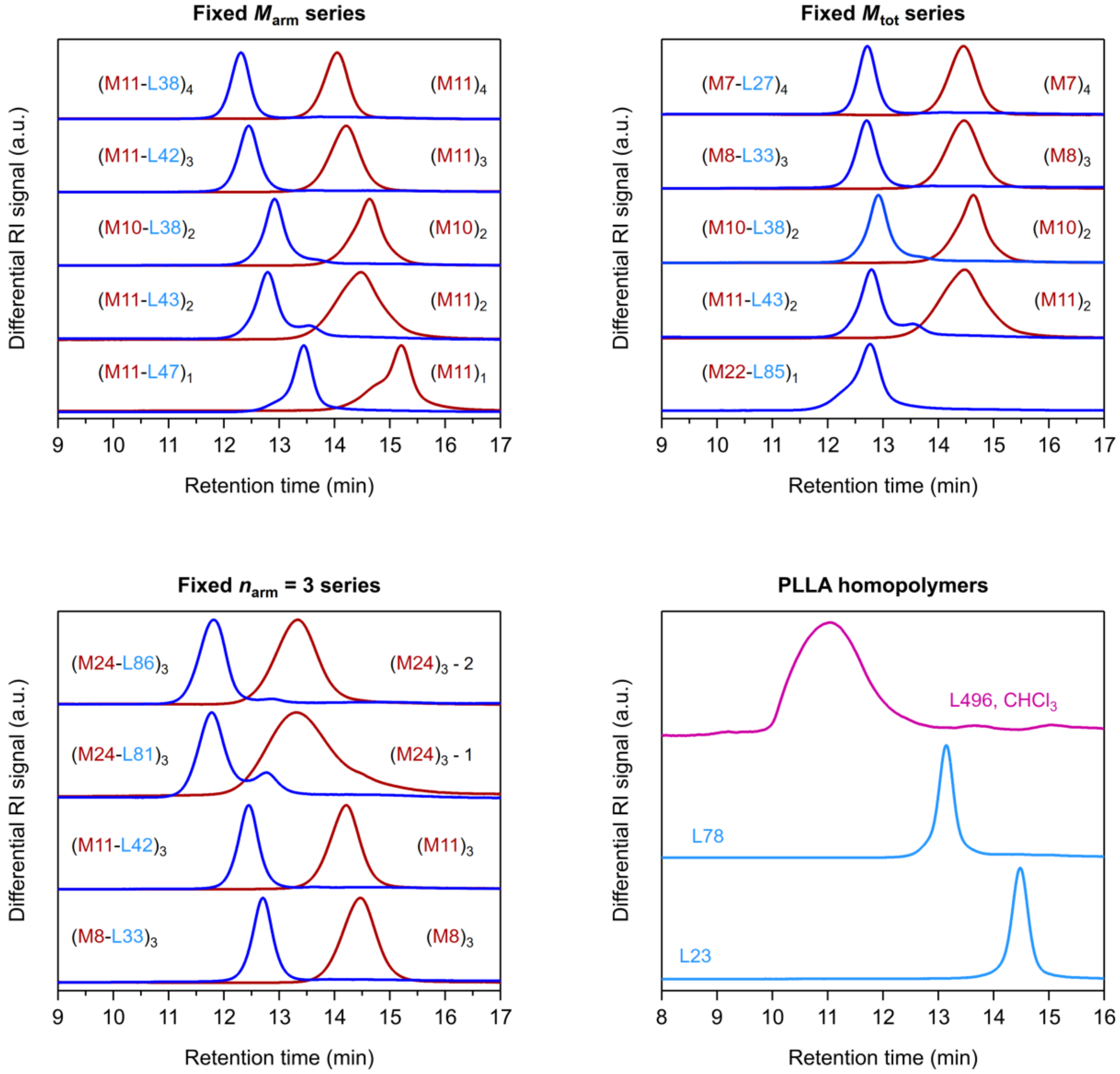

Figure S11. Size exclusion chromatograms of polymers synthesized in this study. Monofunctional macroinitiator $(M22)_1$'s trace is missing because it was exhausted in other SEC analyses on a separate instrument that ultimately could not produce reliable data.

Table S2. Molecular characteristics of synthesized $(M)_n$ PγMCL macroinitiators.

Material	$M_{n,\ P\gamma MCL\ arm,\ NMR}$ (kg mol^{-1})	$M_{n,\ SEC,\ RI}$ (kg mol^{-1})	$Đ_{SEC,\ RI}$
$(M11)_1$	11.5	15.9	1.27
$(M22)_1$	22.0	– [a]	– [a]
$(M11)_2$	11.0	31.7	1.34
$(M10)_2$	10.2	28.8	1.14
$(M8)_3$	8.0	35.6	1.16
$(M11)_3$	10.8	48.5	1.12
$(M24)_3$ – 1 for $(M24\text{-}L81)_3$	24.0	68.9	1.75
$(M24)_3$ – 2 for $(M24\text{-}L86)_3$	24.4	95.8	1.27
$(M7)_4$	7.0	37.9	1.09
$(M11)_4$	10.6	58.1	1.09

[a] $(M22)_1$ was exhausted in other SEC analyses on a separate instrument that ultimately could not produce reliable data.

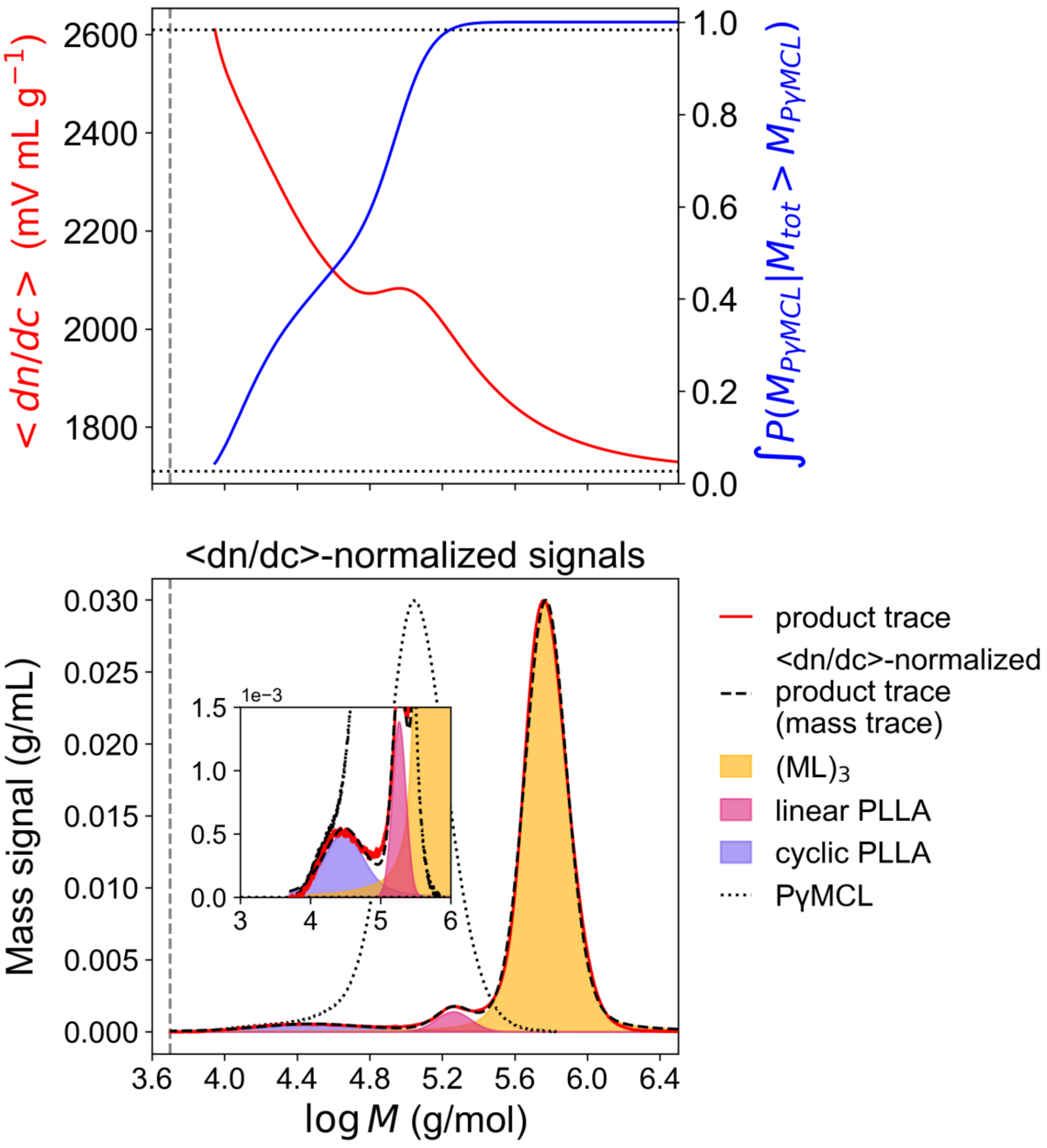

Figure S12. Example of SEC purity analysis output for $(M24\text{-}L86)_3$.[11] Top: slice-average (dn/dc) and probability normalization factor (ensuring that all PγMCL molar masses used to calculate a slice-average (dn/dc) for the $(ML)_n$ product are smaller than those of the product). Bottom: deconvolution of SEC traces after normalization by slice-average (dn/dc); peak areas are then species weight fractions.

Table S3. Molecular and purity characteristics of synthesized $(ML)_n$ star-block polymers and PLLA homopolymers. The final two columns, w_{target}, identify the weight fraction of target species (*i.e.*, the fully chain-extended PγMCL with the correct number of arms).

Material	$w_{PLLA, NMR}$	$M_{n, PLLA arm, NMR}$ (kg mol^{-1})	$M_{n, SEC, RI}$ (kg mol^{-1})	$Đ_{SEC, RI}$	$w_{target, NMR}$ [a]	$w_{target, SEC}$ [b]
$(M11\text{-}L47)_1$	0.81	46.6	88.3	1.29	0.98	0.81
$(M22\text{-}L85)_1$	0.80	84.8	178	1.22	0.96	0.75 [c]
$(M11\text{-}L43)_2$*	0.81	42.7	134	1.35	0.92	0.74
$(M10\text{-}L38)_2$	0.80	37.5	149	1.14	0.97	0.92
$(M8\text{-}L33)_3$	0.81	32.8	213	1.03	0.97	0.88
$(M11\text{-}L42)_3$	0.81	41.6	261	1.03	0.99	0.84
$(M24\text{-}L81)_3$*	0.80	81.0	341	1.12	0.88	0.49
$(M24\text{-}L86)_3$	0.78	86.5	498	1.19	0.97	0.93
$(M7\text{-}L27)_4$	0.79	26.9	210	1.03	– [d]	0.86
$(M11\text{-}L38)_4$	0.78	38.4	293	1.03	–	0.81
L23	1	22.6	35.4	1.11	–	–
L78	1	78.1	144	1.04	–	–
L500	1	496	1100	1.44	–	–

[a] $w_{target, NMR}$ is calculated by using the relative integrals of initiator mid-group, PLLA end-group, and PγMCL methyl ester CH_3 protons to algebraically derive the molar abundances of target and impurity species, which are then converted to weight fractions assuming number-average block sizes for all species. Details are in our previous publication.

[b] $w_{target, SEC}$ is calculated by normalizing the product SEC trace by the "slice average (dn/dc)" and then deconvoluting the product and impurity peaks as detailed in our previous publication.

[c] The $(M22)_1$ PγMCL macroinitiator SEC trace could not be collected because the material was exhausted in other analyses, so this w_{target} was obtained from simple deconvolution without correcting for (dn/dc).

[d] The CH_2 protons on pentaerythritol are not visible in the product ^{1}H-NMR spectrum, which prevents the calculation of a theoretical w_{target} for the 4-arm star-blocks synthesized here.

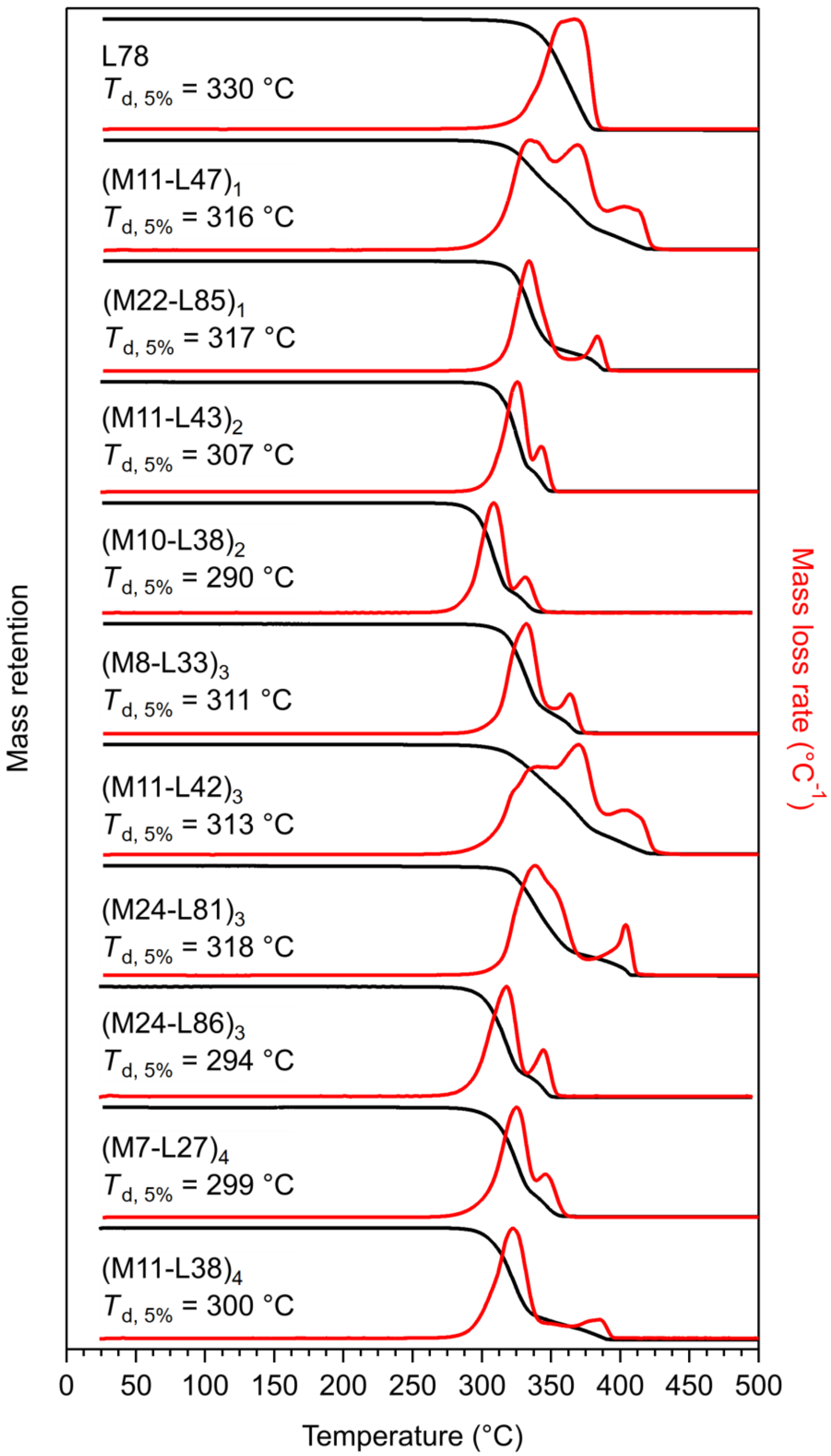

Figure S13. Mass retention (left axis) and mass loss rate (right axis) traces obtained from TGA. Temperatures at 5% mass loss ($T_{d, 5\%}$) are indicated next to each trace.

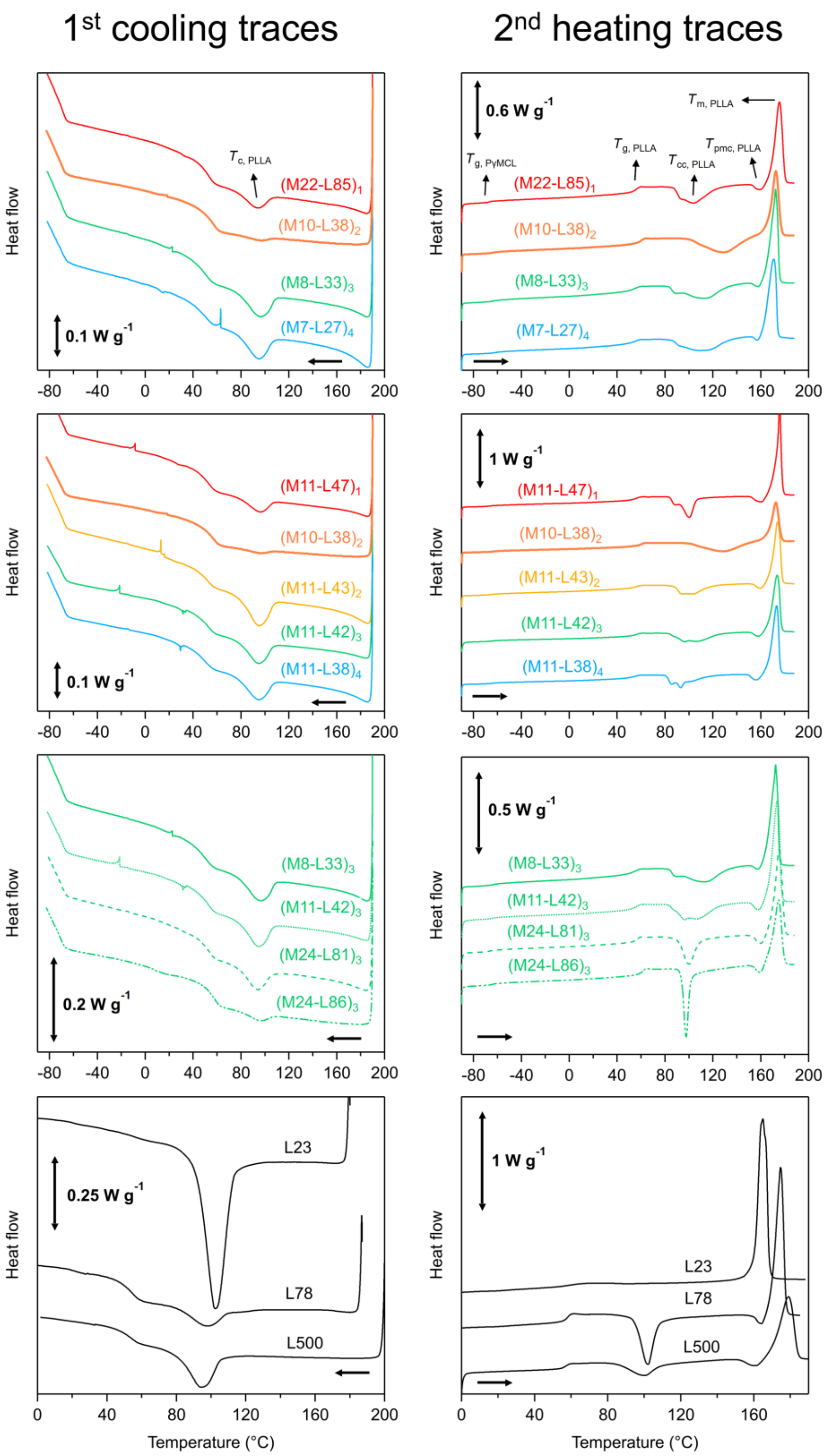

Figure S14. Dynamic DSC traces. The double-headed arrow in each panel defines the vertical heat flow scale.

Table S4. Thermal characteristics recorded in DSC experiments. Materials with two listed T_{cc} values exhibited multimodal cold crystallization; each peak is identified.

Material	1st cool	2nd heating characteristics					MDSC
	T_c (°C)	$T_{g,\,P\gamma MCL}$ (°C)	$T_{g,\,PLLA}$ (°C)	T_{cc} (°C)	T_m (°C)	X_c (%)	$T_{g,\,PLLA}$ (°C)
$(M11\text{-}L47)_1$	95.51	–65.77	55.51	89, 100	175.67	24	54.97
$(M22\text{-}L85)_1$	93.66	–66.69	55.77	92, 104	175.65	17	56.60
$(M11\text{-}L43)_2$	94.88	–64.75	56.22	95, 103	174.08	26	55.11
$(M10\text{-}L38)_2$	95.72	–64.68	54.63	105	173.61	12	57.22
$(M8\text{-}L33)_3$	95.98	–63.22	55.15	90, 112	172.49	19	54.04
$(M11\text{-}L42)_3$	94.36	–64.23	55.61	97, 106	173.66	23	54.66
$(M24\text{-}L81)_3$	93.42	–66.25	56.42	100	175.41	21	53.51
$(M24\text{-}L86)_3$	94.63	–65.84	55.61	97	175.60	18	58.50
$(M7\text{-}L27)_4$	95.01	–62.26	55.49	92, 112	170.84	16	53.24
$(M11\text{-}L38)_4$	95.79	–63.61	54.53	94, 104	172.68	18	53.95
L23	101.9	–	58.85, broad	91	163.68	60	56.83
L78	97.67	–	57.70	102	174.68	20	58.12
L500	94.11	–	56.93	100	179.12	21	58.34

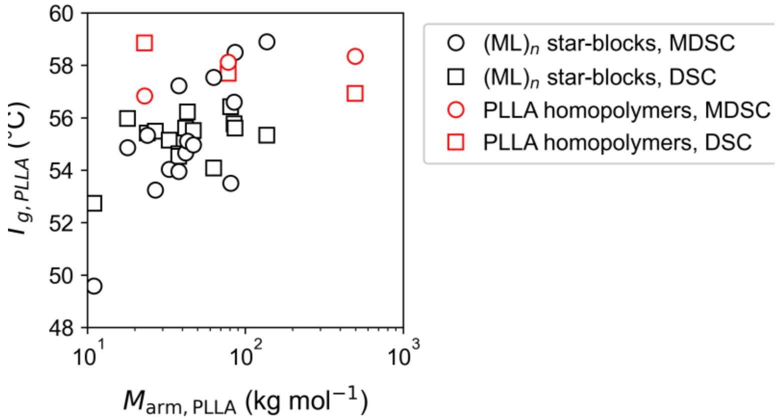

Figure S15. $T_{g,\,PLLA}$ measured both in quasi-isothermal MDSC and dynamic DSC plotted as a function of $M_{arm,\,PLLA}$. This dataset includes the LML triblocks from our previous study.[11]

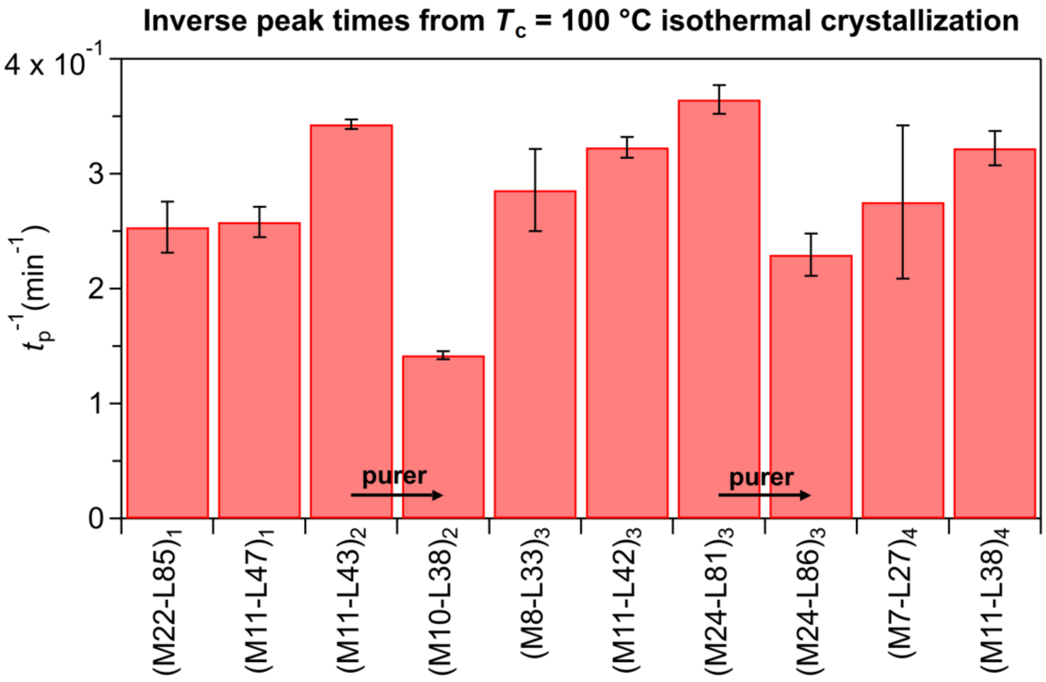

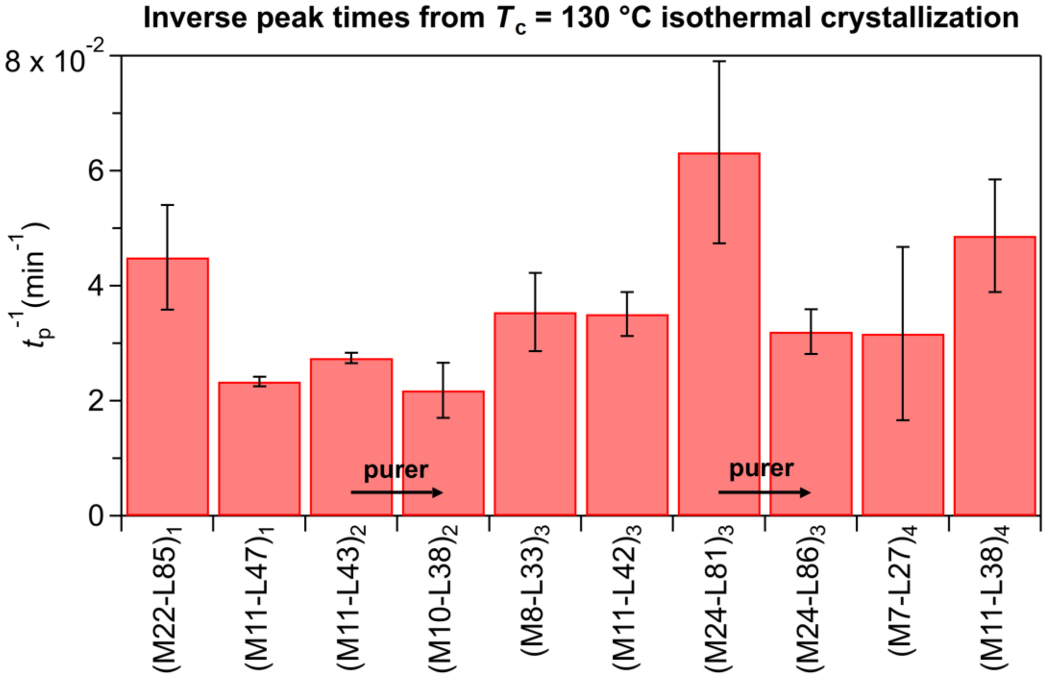

Figure S16. Inverse times at peak exothermic heat flow (t_p^{-1}) captured during isothermal crystallization at 100 °C (top) and 130 °C (bottom) after quenching from the melt. Three replicates were performed for each material. Error bars indicate 95% confidence intervals calculated with a student's test statistic.

As shown above in **Figure S16**, there was no significant trend in isothermal crystallization kinetics except that the more impure star-blocks $(M11\text{-}L43)_2^*$ and $(M24\text{-}L81)_3^*$, rich in PLLA homopolymer impurity, crystallized more rapidly than their higher purity counterparts $(M10\text{-}L38)_2$ and $(M24\text{-}L86)_3$. This was likely due to the untethered PLLA impurity having greater mobility than the tethered outer blocks.[12]

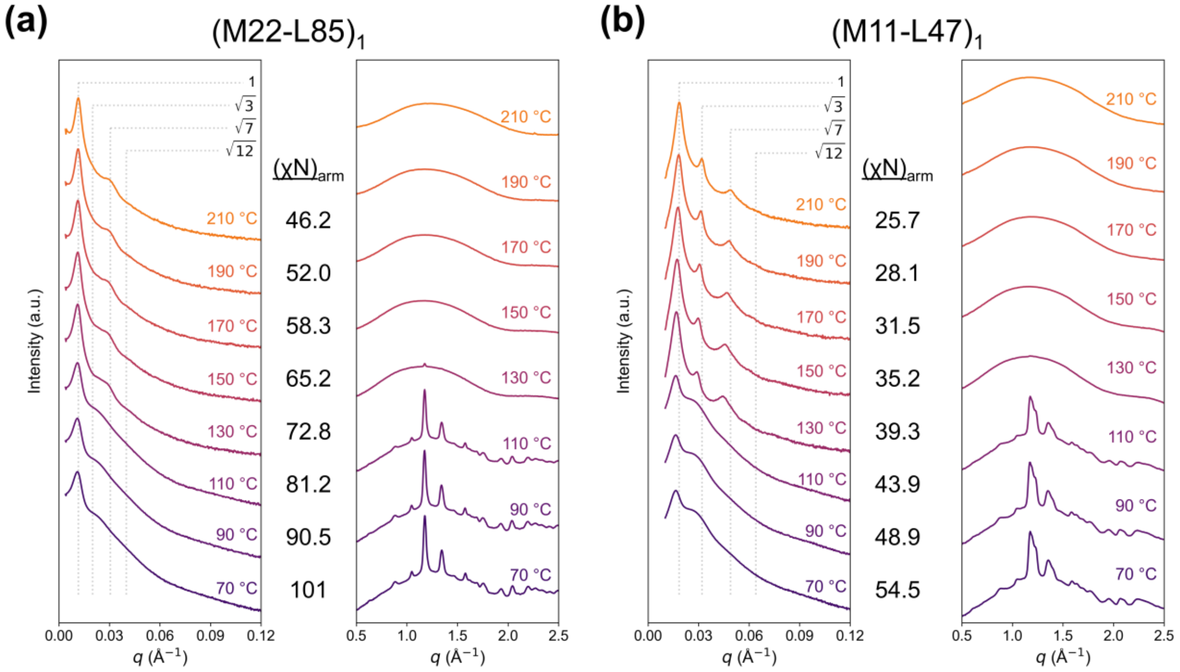

Figure S17. Variable temperature SAXS/WAXS (left/right) patterns obtained for diblocks (a) $(M22\text{-}L85)_1$ and (b) $(M11\text{-}L47)_1$ during staged cooling from 210 to 70 °C. The $(\chi N)_{arm}$ values correspond to the temperatures labeling the SAXS patterns (left).

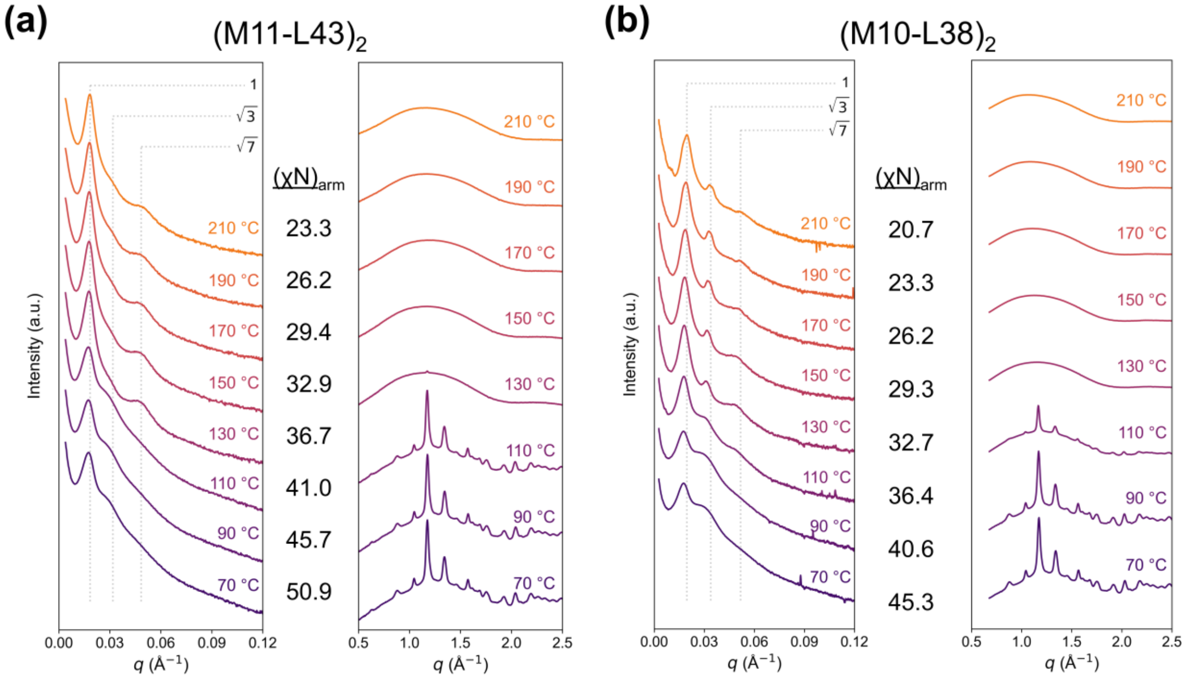

Figure S18. Variable temperature SAXS/WAXS (left/right) patterns obtained for triblocks (a) $(M11\text{-}L43)_2^*$ and (b) $(M10\text{-}L38)_2$ during staged cooling from 210 to 70 °C. The $(\chi N)_{arm}$ values correspond to the temperatures labeling the SAXS patterns (left).

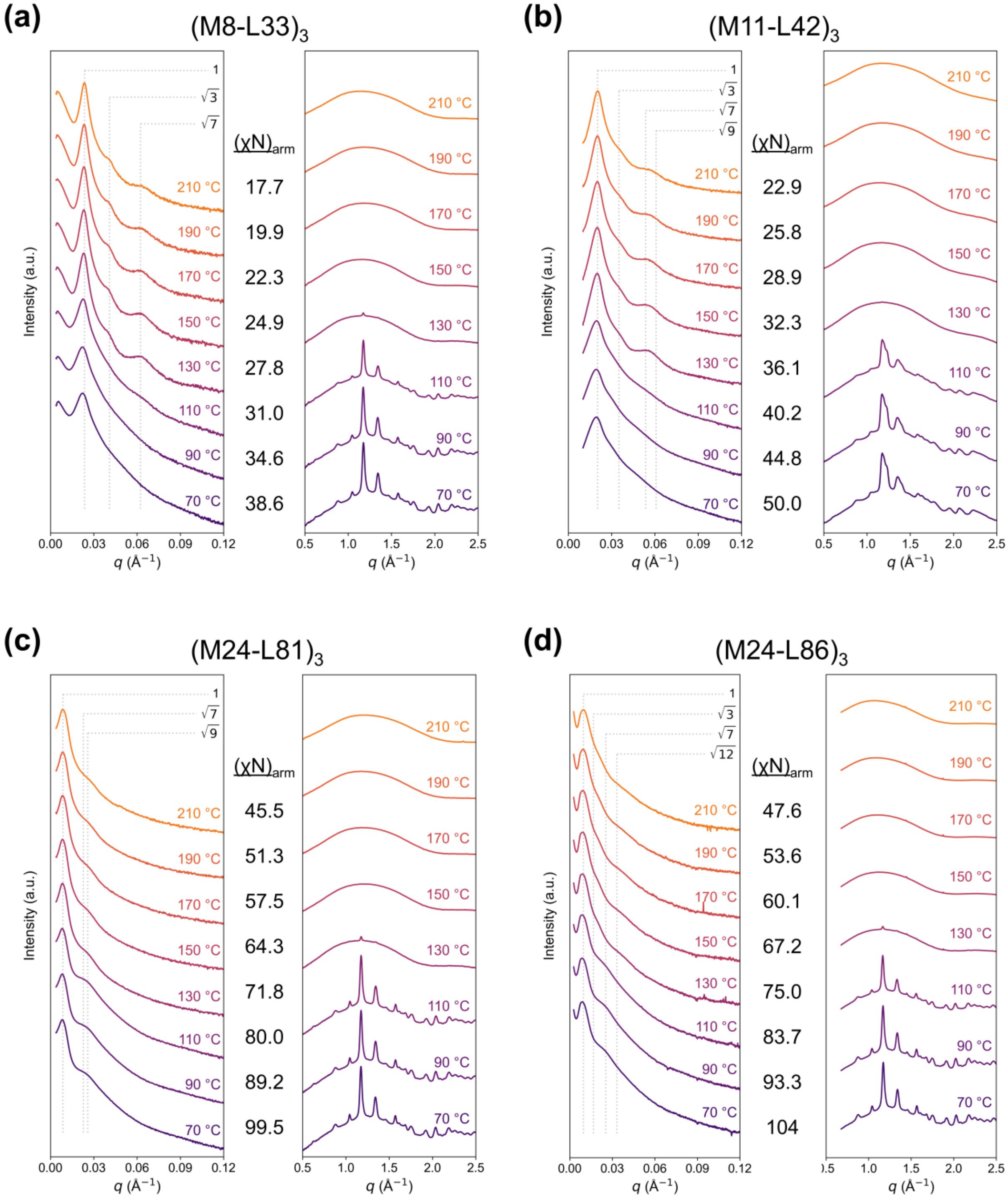

Figure S19. Variable temperature SAXS/WAXS (left/right) patterns obtained for three-arm star-blocks (a) $(M8\text{-}L33)_3$, (b) $(M11\text{-}L42)_3$ (c) $(M24\text{-}L81)_3{}^*$, and (d) $(M24\text{-}L86)_3$ during staged cooling from 210 to 70 °C. The $(\chi N)_{arm}$ values correspond to the temperatures labeling the SAXS patterns (left).

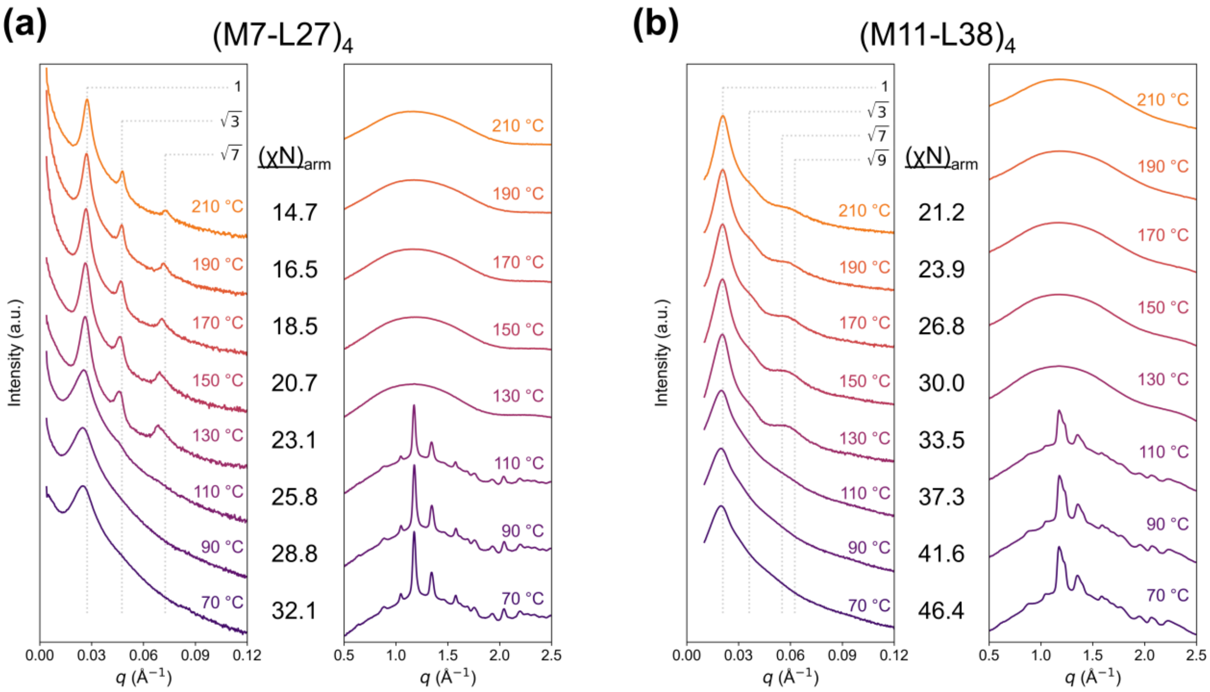

Figure S20. Variable temperature SAXS/WAXS (left/right) patterns obtained for four-arm star-blocks (a) $(M7\text{-}L27)_4$ and (b) $(M11\text{-}L38)_4$ during staged cooling from 210 to 70 °C. The $(\chi N)_{arm}$ values correspond to the temperatures labeling the SAXS patterns (left).

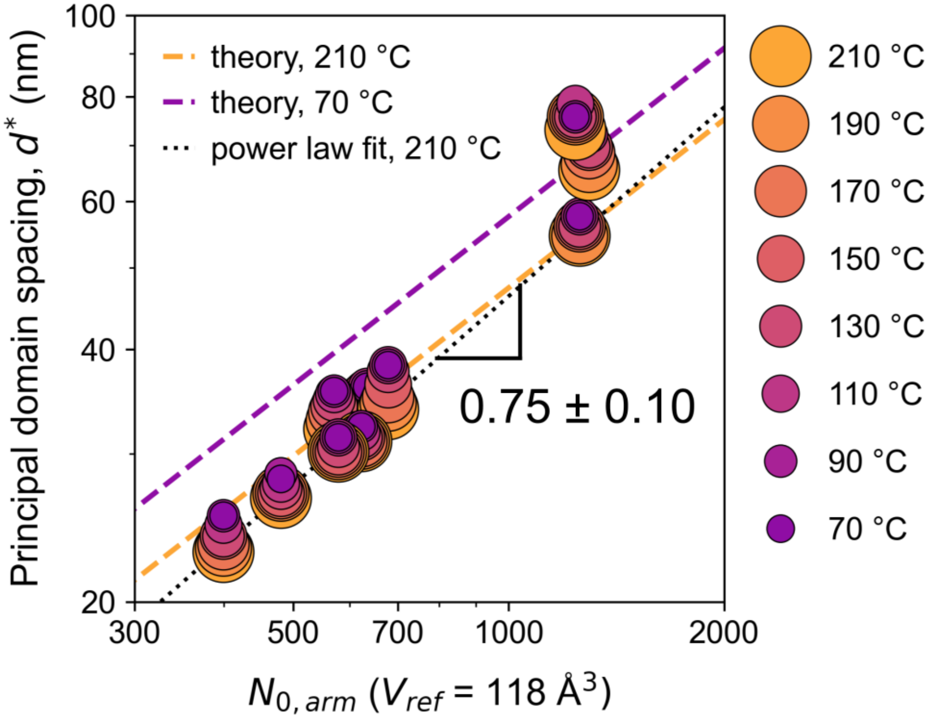

Figure S21. Principal domain spacings ($d^* = 2\pi / q^*$) plotted as a function of volume-referenced arm degree of polymerization ($N_{0,\text{ arm}}$) during staged cooling of $(ML)_n$ star-blocks. The power-law fit of spacings at 210 °C has a slope of 0.75 ± 0.10 (95% confidence interval), which is within error of the 2/3 scaling predicted for strong segregation. Based on Matsen's self-consistent field theory results for diblock-arm star-block polymers, the estimated order-disorder transition temperatures (T_{ODT}) for these materials are all at or above 230 °C.[13] We excluded the two largest three-arm star-blocks $(M24\text{-}L81)_3$ and $(M24\text{-}L86)_3$ because of their large positive deviations and the likelihood that ^{1}H-NMR spectroscopy underestimated their $M_{\text{arm, PLLA}}$, which would cause an erroneous leftward shift on the above plot. The theoretical domain spacings were obtained using Matsen and Bates's expression for the hexagonally packed cylinder morphology—HEX is clearly the equilibrium morphology for this system at fixed $w_{PLLA} = 0.8$, see **Figures S17-S20** above)—of a diblock polymer with compositionally asymmetric blocks.[14] This expression requires the two blocks' statistical segment lengths. The statistical segment length of PLLA was estimated using Anderson and Hillmyer's values[15] in combination with Witzke's expression for the temperature-dependent density of PLLA[16] to properly adjust the reference volume with temperature and convert to the result for a 118 Å^3 reference volume. The statistical segment length of PγMCL was estimated as described by Karavolias (Appendix 3, page 141)[17]:

$$b_{P\gamma MCL} = \frac{m_0^{\frac{1}{2}}}{M_e^{\frac{1}{6}}(A\rho N_{av})^{\frac{1}{3}}}$$

$$= \frac{\left(128.17\,\frac{g}{mol}\right)^{\frac{1}{2}}}{\left(2.9\times10^{3}\,\frac{g}{mol}\right)^{\frac{1}{6}}\left((0.057835)\left(1.037\,\frac{g}{cm^{3}}\right)(6.022\times10^{23}\,mol^{-1})\right)^{\frac{1}{3}}}$$

$$= \mathbf{9.0696}\ \text{Å}$$

where $M_{\text{e, P}\gamma\text{MCL}}$ = 2.9 kg mol^{-1} and $\rho_{\text{P}\gamma\text{MCL}}$ = 1.037 g cm^{-3} as determined by Watts *et al.*[18] Then, this value was adapted to a 118 Å^3 reference volume:

$$\frac{b_0^2}{v_0} = \frac{b_1^2}{v_1};\ v_0 = \frac{m_0}{\rho N_{av}} = 205\ \text{Å}^3 \quad\Rightarrow\quad b_{118} = (9.0696\ \text{Å})\left(\frac{118\ \text{Å}^3}{205\ \text{Å}^3}\right)^{\frac{1}{2}} = \mathbf{6.88}\ \text{Å}$$

No temperature dependence was assumed for this value, which may add error to the calculation. As seen in **Figure S21**, most of the star-blocks show negative deviations from the predictions, with the most severe deviations appearing at low arm length. This may be due to the fact that decreasing the length of the PγMCL arm while increasing arm number reduces the excluded volume relative to a linear PγMCL of identical molar mass, reducing the domain size and, in turn, the domain spacing.

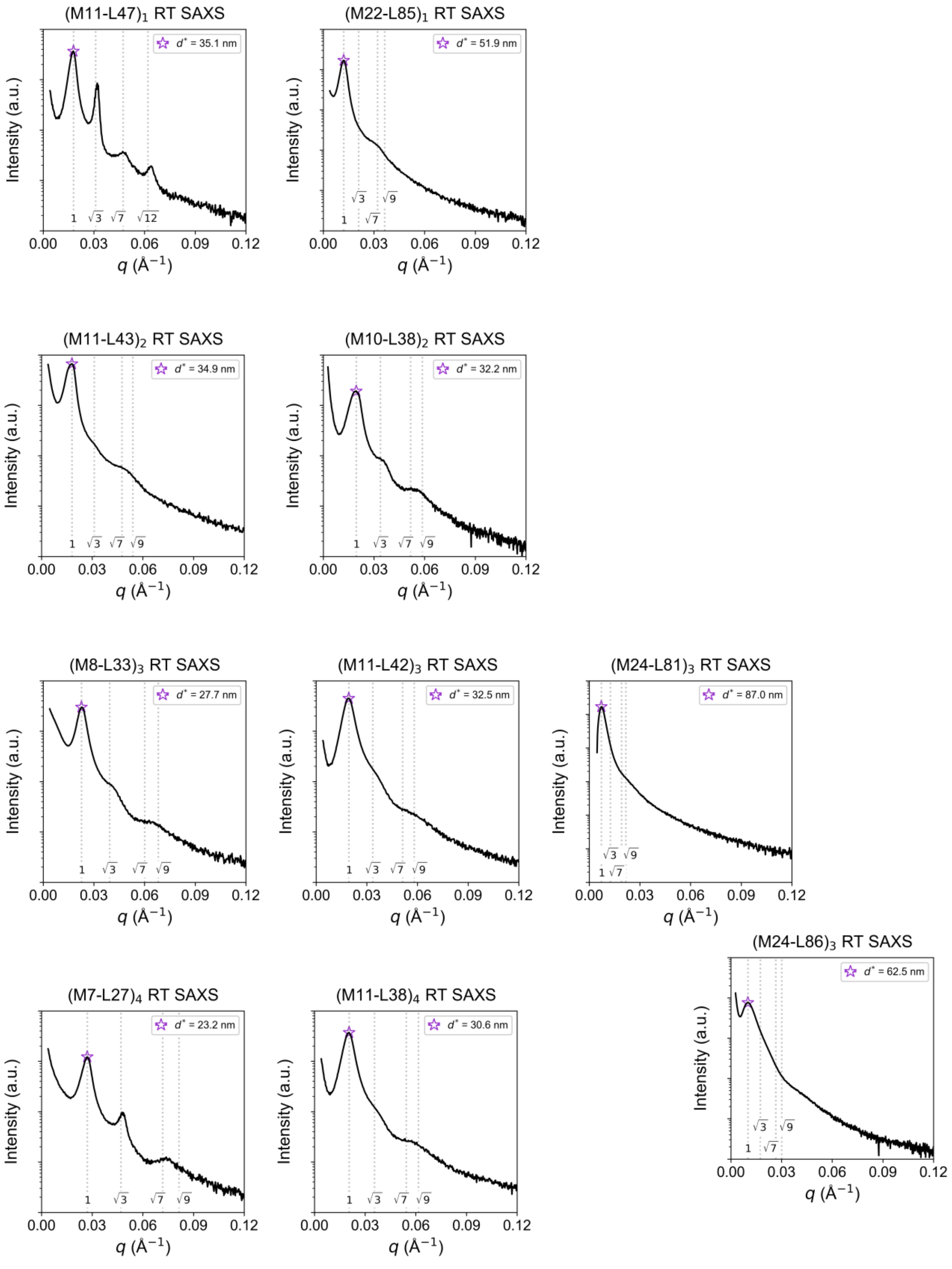

Figure S22. Room temperature SAXS patterns of all $(ML)_n$ star-blocks.

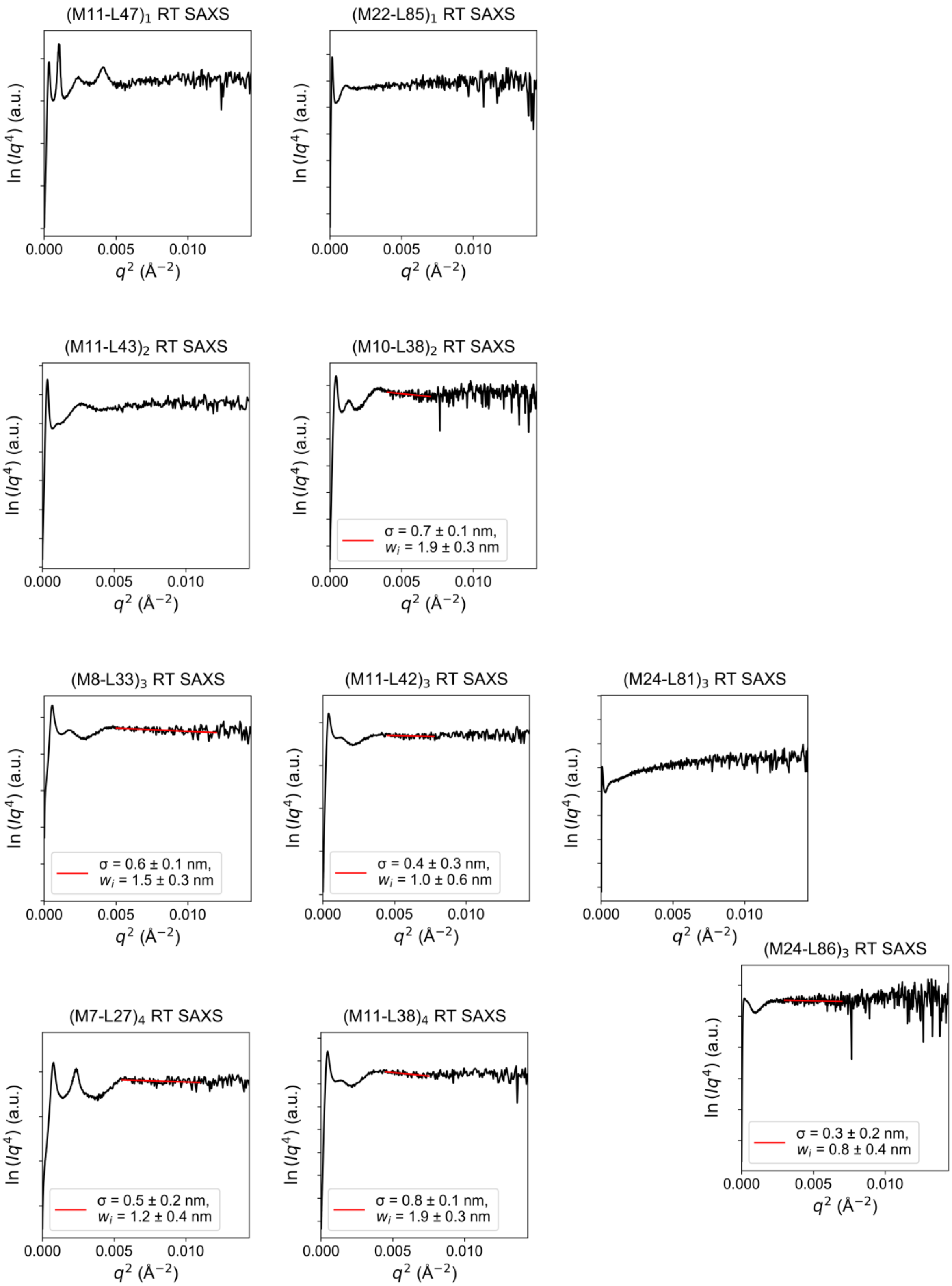

Figure S23. Room temperature SAXS patterns linearized according to the model $I(q) \sim q^{-4}\exp(-\sigma^2 q^2)$. Only the specimens with labeled fits (red lines) showed a region with a slope whose 95% confidence interval excluded zero.

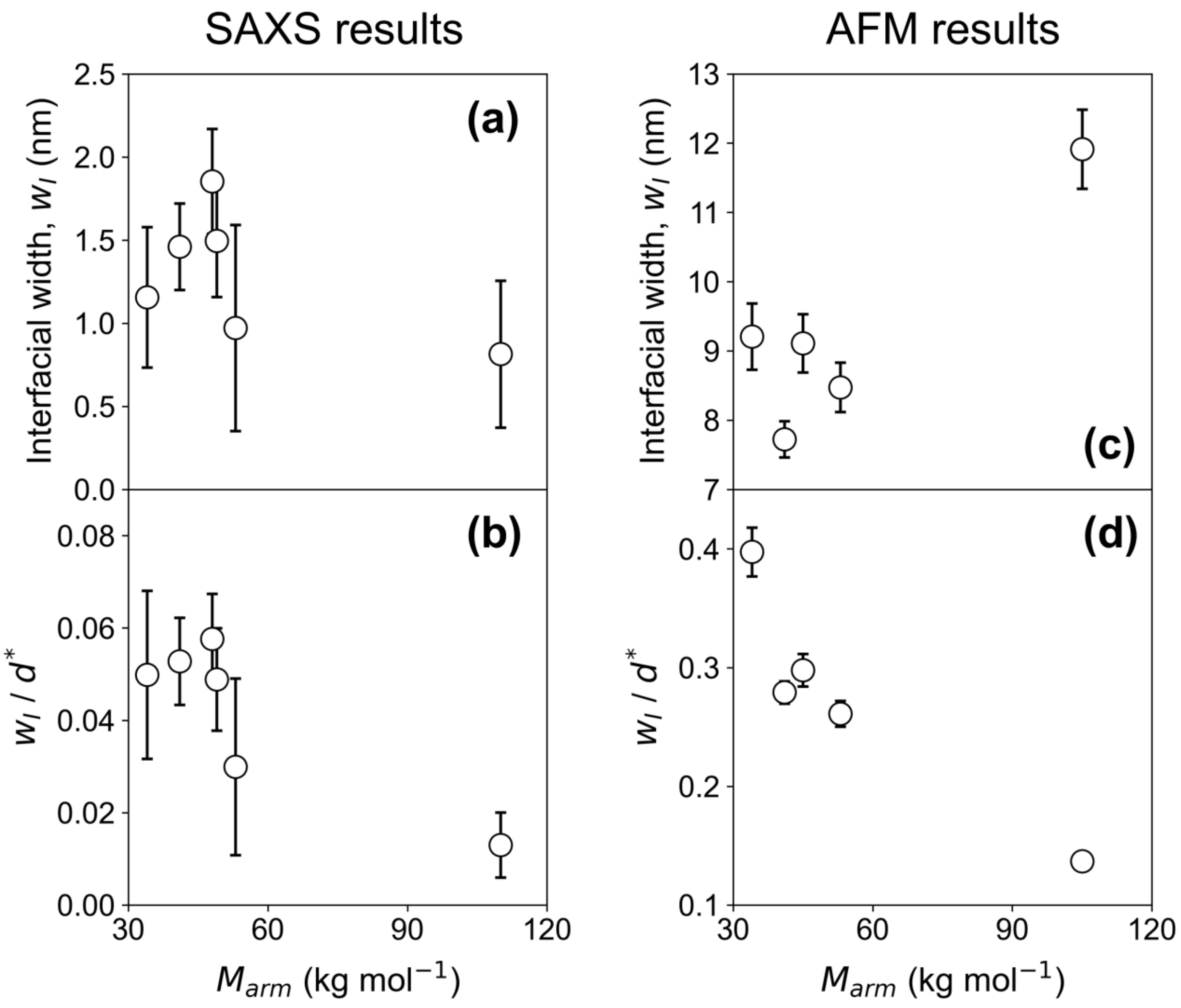

Figure S24. Interfacial widths (top) and interfacial widths normalized by principal PγMCL domain spacing (bottom) obtained from SAXS (left) and AFM (right). Note the vertical scale differences; here, qualitative trends were most useful. Error bars denote 95% confidence intervals calculated using a Z-statistic.

As part of our mechanistic rationale for the aging and mechanical behavior of the $(ML)_n$ star-blocks, we invoke Priestley *et al.*'s observation that the width of the T_g gradient region in a glassy-rubbery block polymer system is independent of the block polymer's degree of polymerization (N).[19] In other words, the *dynamic* interfacial width—that is, the width of the region with intermediate T_g and chain dynamics between the bulk behavior of the two blocks—does not vary with overall chain size. Because the principal domain spacing (d^*) scales with $N^{2/3}$ in the strong segregation limit,[20] where our $(ML)_n$ materials exist ($\chi N > 30$ at room temperature), we would therefore expect such an interfacial width, when scaled by d^*, to decrease with increasing

N. To test this hypothesis for our system, we used SAXS and AFM to estimate an interfacial width (denoted w_I) (**Figures S24a** and **S24c**) and scaled it by the observed d^* to produce w_I / d^* (**Figures S24b** and **S24d**). From SAXS, we estimated w_I from the high-q Porod scattering regime of rapidly quenched, low-crystallinity samples as described in the Methods section. From AFM, we took w_I to be the width of the linear inflectional range of phase change between the PγMCL domains and the PLLA matrix. (See the AFM data below in **Figures S58-S63**). It is important to note that neither of these techniques directly probes for chain dynamics, so w_I is not the T_g gradient region. Porod scattering in SAXS reports on electron density fluctuations (*i.e.*, compositional in nature) through the interface, while AFM phase reports on dissipation of the oscillating cantilever tip's mechanical energy.[21] But if either of these techniques showed that the opposite trend were true, that w_I / d^* increased with M_{arm}, then this would cast doubt on the validity of applying Priestley *et al.*'s findings to our system. Both techniques show the trend expected under Priestley *et al.*'s framework: w_I / d^* decreases with increasing M_{arm}. $(M24\text{-}L81)_3{}^*$, when analyzed through AFM, shows a far larger w_I than the other samples (**Figure S24c**). We suspect that its high molar mass slowed melt diffusion and kinetically limited the assembly of a sharp interface. Nonetheless, after normalization by d^*, $(M24\text{-}L81)_3{}^*$'s w_I conforms to the expected trend.

Table S5. Tensile properties of materials rapidly quenched from the melt (low crystallinity). "±" indicates 95% confidence intervals calculated with a student's *t*-statistic.

Material, age (days)	n_{sample}	$n_{brittle}$	E (GPa)	σ_y (MPa)	ε_b (%)	σ_b (MPa)	U_T (MJ m^{-3})	G_R (MPa)
$(M11\text{-}L47)_1$, 3	6	4	1.77 ± 0.09	41.8 ± 1.9	32.8 ± 22.3	23.6 ± 4.9	9.52 ± 5.09	
$(M11\text{-}L47)_1$, 80	5	5	2.00 ± 0.20	41.9 ± 2.0	7.05 ± 6.96	29.3 ± 4.5	3.47 ± 2.46	
$(M22\text{-}L85)_1$, 3	7	7	1.94 ± 0.08	36.6 ± 2.7	5.18 ± 1.93	24.5 ± 6.0	2.06 ± 0.76	
$(M22\text{-}L85)_1$, 80	5	5	1.96 ± 0.05	40.6 ± 0.7	4.85 ± 0.63	29.8 ± 7.6	2.37 ± 0.93	
$(M11\text{-}L43)_2$, 3	5	0	1.87 ± 0.12	36.0 ± 3.7	311 ± 24	30.6 ± 1.4	76.4 ± 5.7	
$(M11\text{-}L43)_2$, 80	5	5	1.88 ± 0.07	43.8 ± 1.3	12.8 ± 6.4	31.9 ± 4.0	4.82 ± 1.4	
$(M10\text{-}L38)_2$, 3	5	0	1.89 ± 0.06	40.5 ± 1.8	287 ± 59	29.1 ± 4.5	75.2 ± 16.9	
$(M10\text{-}L38)_2$, 80	5	2	1.96 ± 0.06	47.8 ± 0.8	129 ± 83	30.4 ± 3.5	39.8 ± 26.6	
$(M10\text{-}L38)_2$, 267	6	2	1.90 ± 0.08	47.4 ± 2.1	157 ± 113	30.2 ± 1.5	48.9 ± 35.5	
$(M8\text{-}L33)_3$, 3	5	0	1.71 ± 0.06	35.7 ± 2.1	339 ± 22	35.1 ± 1.6	85.7 ± 6.4	8.98 ± 0.75
$(M8\text{-}L33)_3$, 80	4	0	1.65 ± 0.18	43.1 ± 2.3	314 ± 84	35.3 ± 6.5	89.6 ± 27.7	8.66 ± 1.66
$(M8\text{-}L33)_3$, 302	4	0	1.68 ± 0.08	41.6 ± 0.4	72.9 ± 43.3	25.2 ± 0.5	19.2 ± 10.9	
$(M11\text{-}L42)_3$, 3	5	0	1.62 ± 0.12	39.5 ± 0.5	285 ± 46	30.9 ± 2.4	73.4 ± 13.1	7.22 ± 0.70
$(M11\text{-}L42)_3$, 80	3	0	1.76 ± 0.14	42.7 ± 2.6	162 ± 209	28.0 ± 6.2	43.1 ± 57.2	
$(M24\text{-}L81)_3$, 3	8	0	1.88 ± 0.10	38.1 ± 1.1	180 ± 72	25.2 ± 1.5	43.0 ± 17.2	
$(M24\text{-}L81)_3$, 80	5	4	2.06 ± 0.12	40.8 ± 0.8	8.72 ± 10.55	31.7 ± 4.8	3.22 ± 2.93	
$(M24\text{-}L86)_3$, 3	5	0	1.67 ± 0.10	33.6 ± 0.9	281 ± 44	29.2 ± 3.4	66.9 ± 12.7	7.71 ± 0.94
$(M24\text{-}L86)_3$, 80	7	0	1.99 ± 0.12	43.2 ± 0.6	181 ± 84	32.2 ± 4.0	54.8 ± 26.1	
$(M7\text{-}L27)_4$, 3	5	0	1.78 ± 0.14	37.3 ± 3.5	297 ± 46	32.3 ± 4.3	74.3 ± 15.6	8.28 ± 0.98
$(M7\text{-}L27)_4$, 80	4	0	1.80 ± 0.07	43.7 ± 3.2	234 ± 129	32.0 ± 8.7	66.0 ± 39.1	
$(M7\text{-}L27)_4$, 218	2	0	2.04	46.7	168	30.6	50.6	
$(M11\text{-}L38)_4$, 3	5	0	1.64 ± 0.04	40.0 ± 1.3	285 ± 29	30.9 ± 2.3	74.7 ± 9.2	8.96 ± 3.47
$(M11\text{-}L38)_4$, 80	3	0	1.89 ± 0.11	44.5 ± 3.6	152 ± 220	27.9 ± 2.2	41.7 ± 59.7	
$(M11\text{-}L38)_4$, 218	2	0	1.90	42.7	80.9	27.4	22.3	

Table S6. Tensile properties of materials melt crystallized for 5 minutes at 100 °C (high crystallinity). "±" indicates 95% confidence intervals calculated with a student's *t*-statistic.

Material, age (days)	n_{sample}	$n_{brittle}$	E (GPa)	σ_y (MPa)	ε_b (%)	σ_b (MPa)	U_T (MJ m^{-3})	G_R (MPa)
(M11-L47)$_1$, 3	5	4	1.70 ± 0.03	31.3 ± 1.2	15.8 ± 18.2	27.8 ± 4.4	5.52 ± 4.23	
(M11-L47)$_1$, 80	5	5	2.00 ± 0.07	38.5 ± 1.1	9.60 ± 7.47	28.3 ± 3.6	3.64 ± 2.33	
(M22-L85)$_1$, 3	8	6	1.93 ± 0.08	35.5 ± 1.3	13.5 ± 16.7	27.4 ± 3.9	5.01 ± 4.15	
(M22-L85)$_1$, 80	5	5	1.81 ± 0.07	37.1 ± 1.2	7.30 ± 6.51	23.2 ± 10.2	2.38 ± 2.11	
(M11-L43)$_2$, 3	7	0	1.72 ± 0.04	31.8 ± 0.5	266 ± 50	34.1 ± 4.0	74.0 ± 16.0	
(M11-L43)$_2$, 80	1	0	1.68	35.8	261	35.2	76.8	
(M10-L38)$_2$, 3	5	0	1.92 ± 0.10	38.5 ± 1.6	306 ± 59	35.8 ± 5.9	88.7 ± 22.9	
(M10-L38)$_2$, 80	5	0	1.73 ± 0.07	38.4 ± 1.4	268 ± 89	33.4 ± 5.6	75.9 ± 27.9	
(M10-L38)$_2$, 267	6	0	1.77 ± 0.04	41.5 ± 1.9	241 ± 102	35.8 ± 5.3	74.2 ± 33.4	
(M8-L33)$_3$, 3	6	0	1.82 ± 0.04	35.3 ± 1.2	298 ± 48	40.9 ± 4.1	89.0 ± 19.2	9.31 ± 0.81
(M8-L33)$_3$, 80	4	0	1.57 ± 0.21	38.2 ± 1.1	279 ± 57	38.6 ± 6.9	85.4 ± 21.1	8.59 ± 2.52
(M11-L42)$_3$, 3	6	0	1.80 ± 0.12	32.7 ± 1.2	117 ± 30	25.9 ± 1.3	30.4 ± 7.8	
(M11-L42)$_3$, 80	4	0	1.81 ± 0.05	34.6 ± 1.2	196 ± 105	31.2 ± 9.5	53.8 ± 34.7	
(M24-L81)$_3$, 3	8	2	1.97 ± 0.09	35.7 ± 0.5	88.6 ± 54.3	29.7 ± 1.6	25.3 ± 15.3	
(M24-L81)$_3$, 80	5	5	1.99 ± 0.08	40.6 ± 1.6	4.99 ± 2.71	35.1 ± 4.2	1.78 ± 1.23	
(M24-L86)$_3$, 3	5	0	1.57 ± 0.04	30.2 ± 0.6	251 ± 34	38.6 ± 4.0	74.5 ± 13.9	9.37 ± 1.06
(M24-L86)$_3$, 80	6	0	1.77 ± 0.18	41.0 ± 1.4	184 ± 84	40.0 ± 7.1	64.6 ± 31.0	11.0 ± 0.5
(M24-L86)$_3$, 167	2	0	1.68	37.1	172	34.2	53.3	
(M7-L27)$_4$, 3	5	0	1.90 ± 0.08	34.2 ± 2.5	236 ± 44	37.3 ± 5.3	69.7 ± 17.9	8.69 ± 1.25
(M7-L27)$_4$, 80	4	0	1.80 ± 0.16	36.5 ± 2.2	238 ± 49	39.3 ± 4.2	73.4 ± 16.5	9.67 ± 0.72
(M11-L38)$_4$, 3	5	0	1.60 ± 0.04	35.3 ± 2.1	256 ± 31	36.9 ± 2.7	74.2 ± 10.9	9.14 ± 1.01
(M11-L38)$_4$, 80	5	0	1.78 ± 0.07	37.1 ± 1.2	196 ± 69	33.0 ± 5.6	56.3 ± 22.1	9.01 ± 1.94

Table S7. Tensile properties of materials melt crystallized for 10 minutes at 100 °C (high crystallinity). "±" indicates 95% confidence intervals calculated with a student's *t*-statistic.

Material, age (days)	n_{sample}	n_{brittle}	E (GPa)	σ_y (MPa)	ε_b (%)	σ_b (MPa)	U_{T} (MJ m^{-3})	G_{R} (MPa)
$(M8\text{-}L33)_3$, 3	6	0	1.64 ± 0.04	31.6 ± 0.7	291 ± 23	43.7 ± 1.6	93.0 ± 7.6	10.3 ± 1.3
$(M8\text{-}L33)_3$, 80	6	0	1.67 ± 0.13	33.1 ± 1.2	225 ± 26	39.2 ± 2.4	68.9 ± 9.2	10.5 ± 0.8
$(M8\text{-}L33)_3$, 302	1	0	1.88	34.8	209	39.1	65.7	9.92
$(M7\text{-}L27)_4$, 3	8	0	1.61 ± 0.08	29.6 ± 1.0	247 ± 36	39.2 ± 4.0	76.3 ± 13.6	9.81 ± 0.60
$(M7\text{-}L27)_4$, 80	5	0	1.62 ± 0.10	32.5 ± 1.53	246 ± 28	42.1 ± 2.1	79.6 ± 10.7	10.3 ± 0.7
$(M7\text{-}L27)_4$, 292	3	0	1.65 ± 0.07	32.8 ± 2.5	238 ± 84	43.2 ± 9.2	78.8 ± 35.3	11.1 ± 0.2

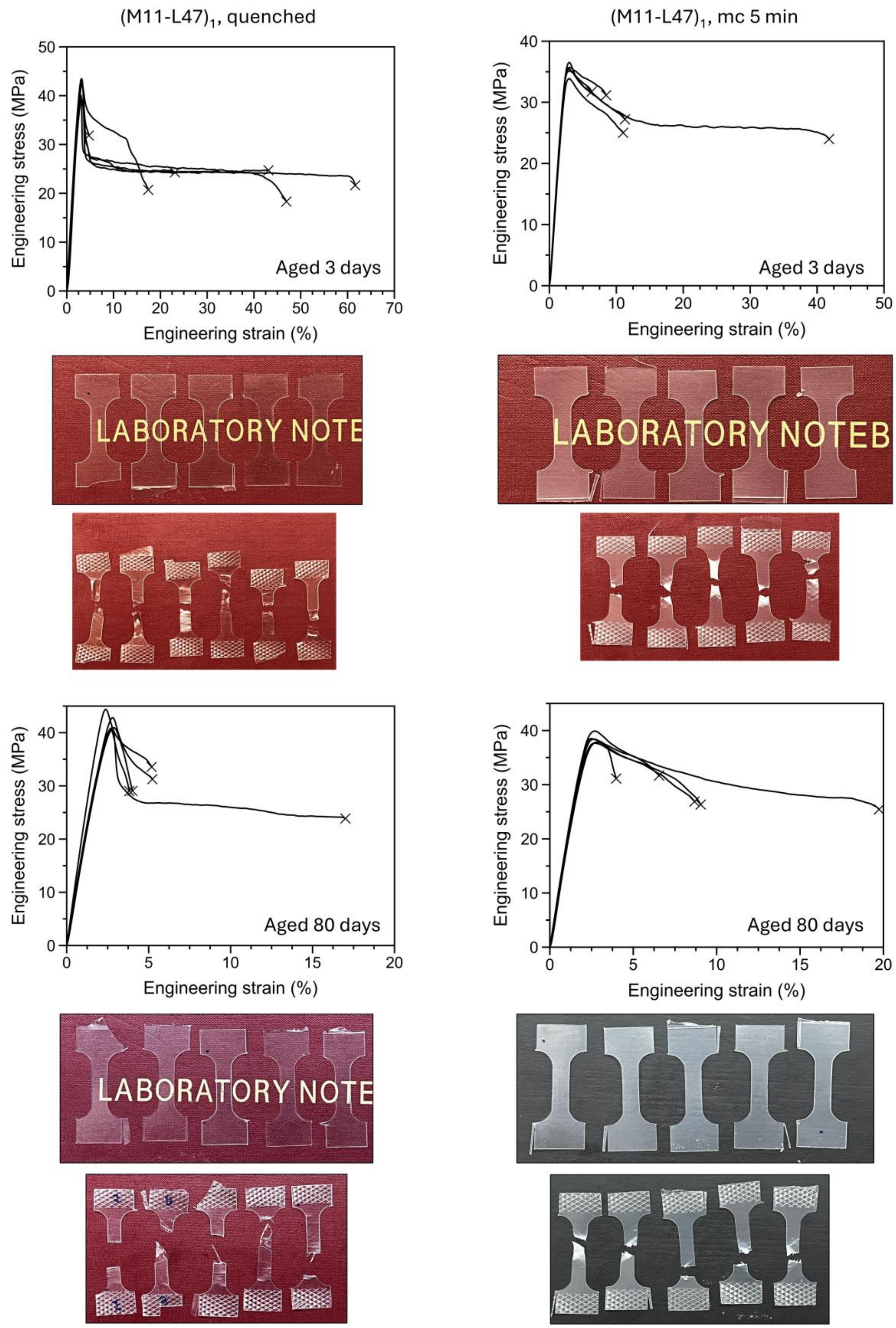

Figure S25. (M11-L47)$_1$ stress strain plots and sample images.

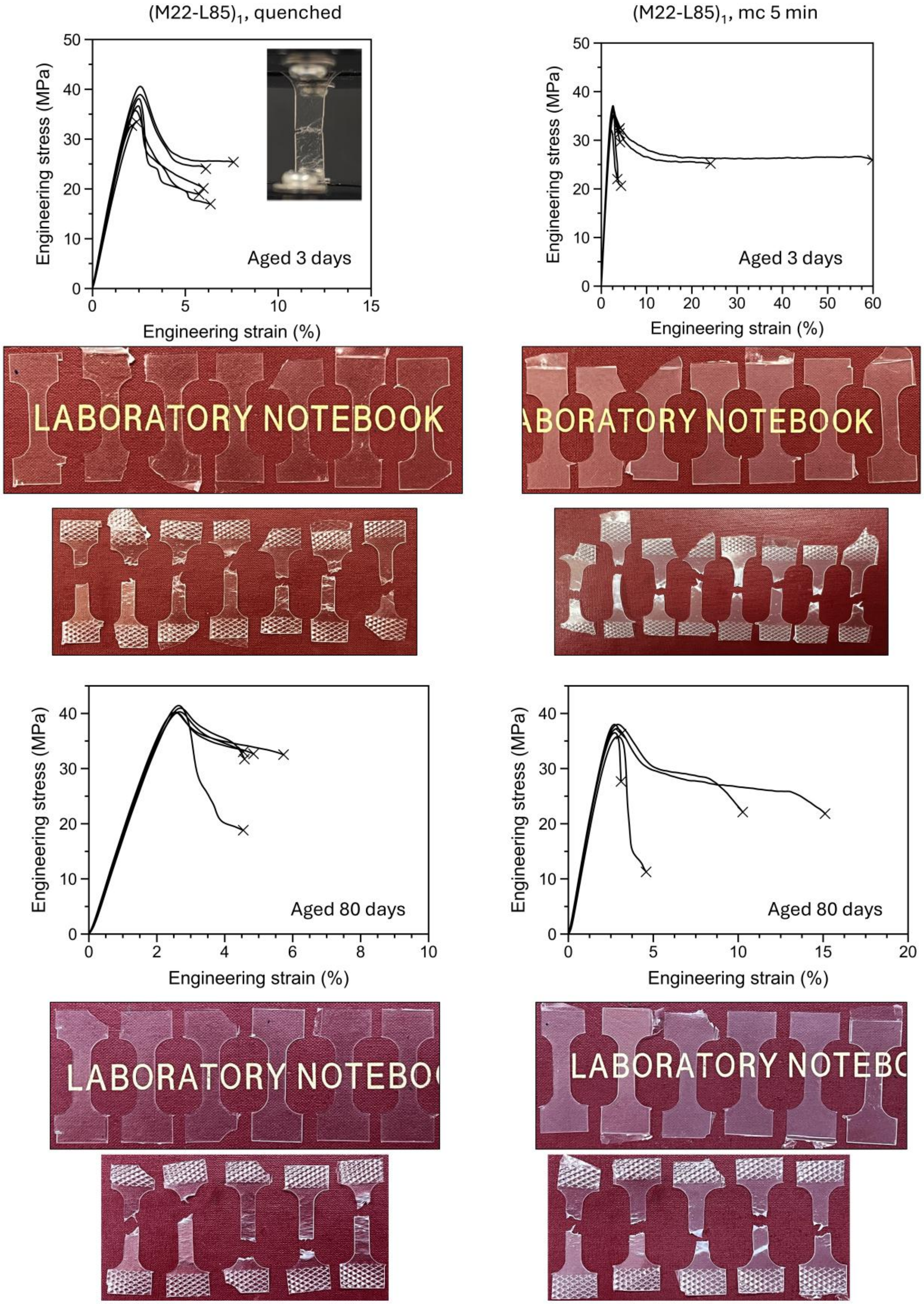

Figure S26. $(M22\text{-}L85)_1$ stress strain plots and sample images.

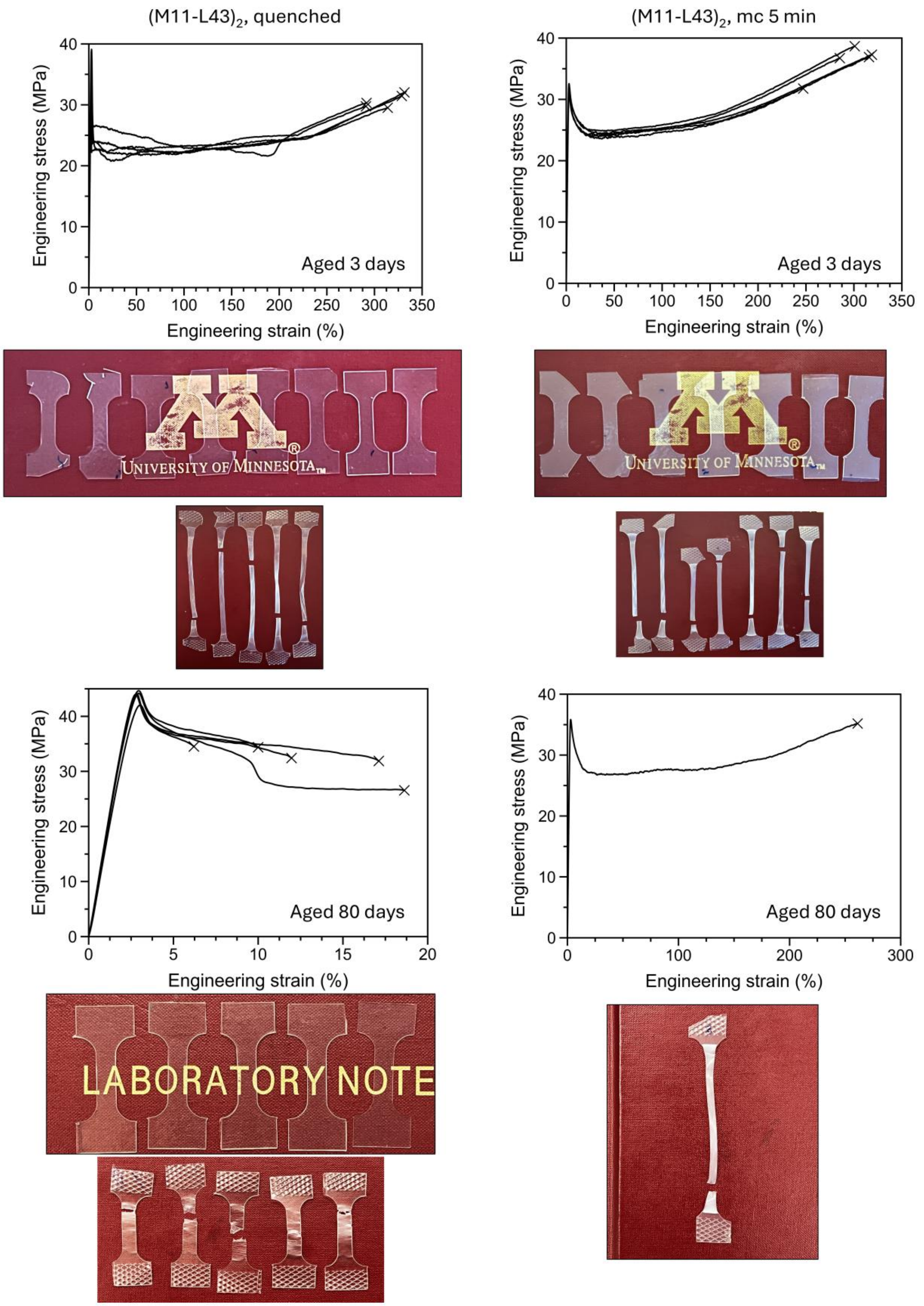

Figure S27. (M11-L43)$_2$ stress strain plots and sample images.

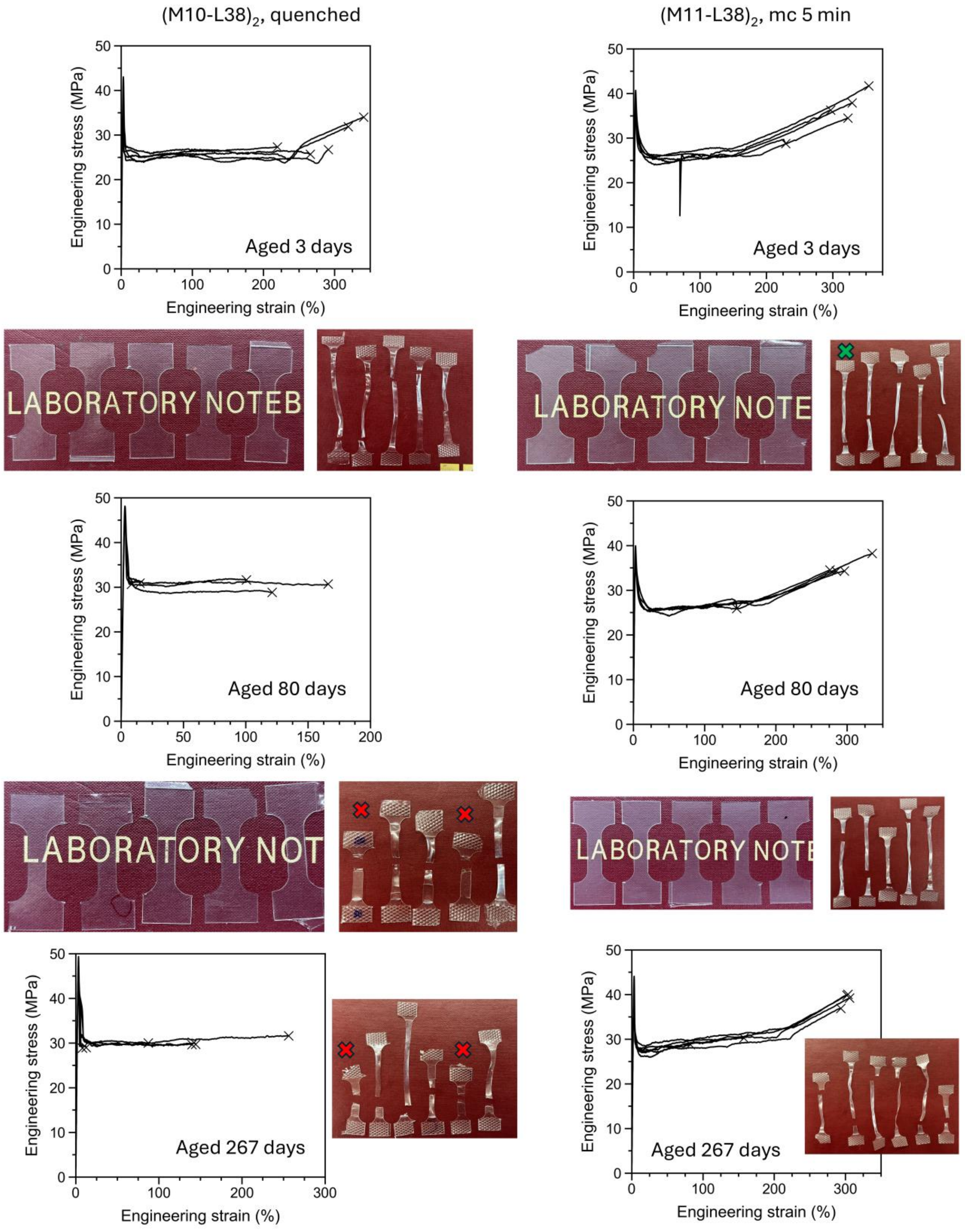

Figure S28. $(M10\text{-}L38)_2$ stress strain plots and sample images.

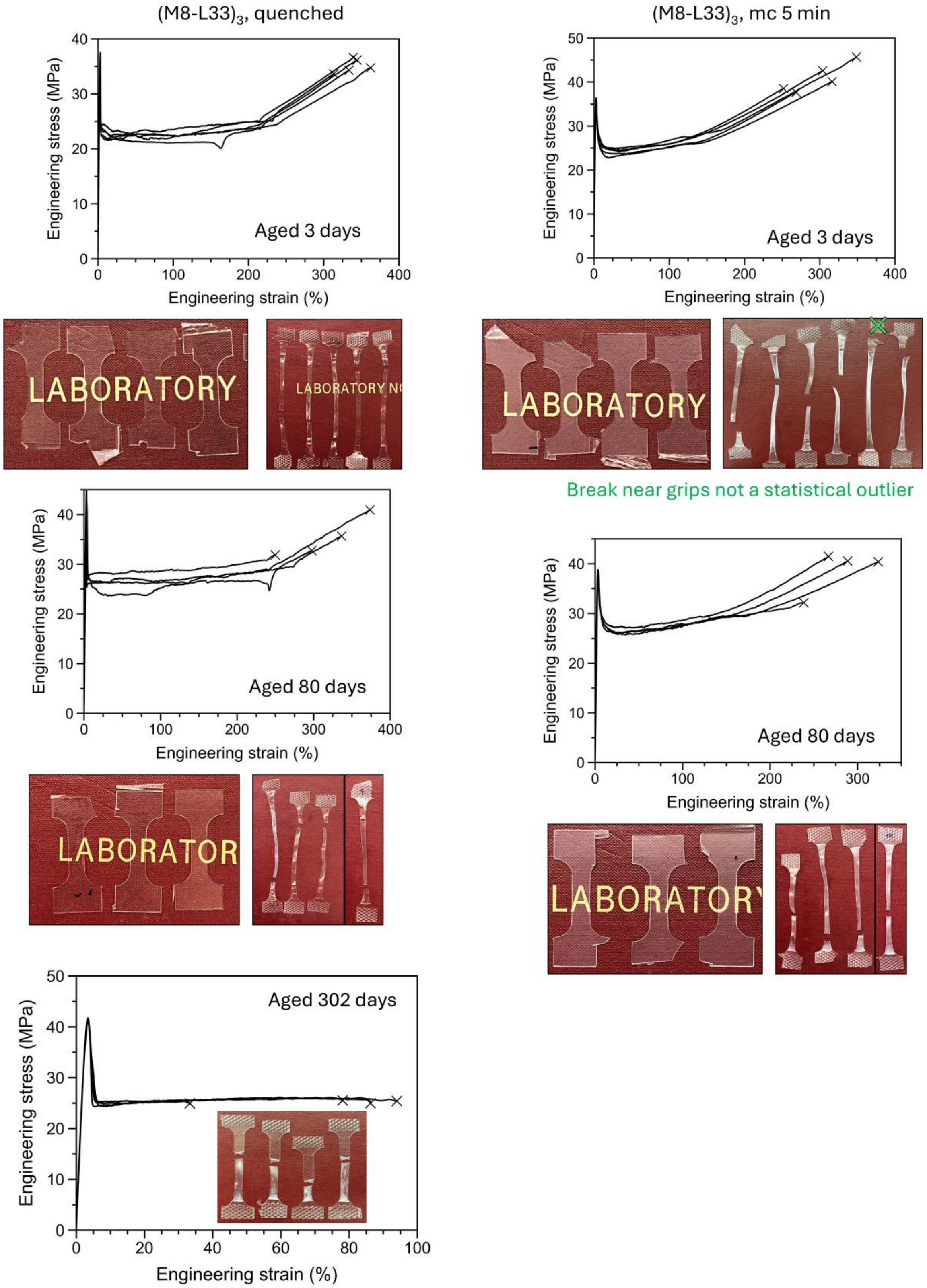

Figure S29. (M8-L33)$_3$ stress strain plots (quenched and melt crystallized 5 min) and sample images.

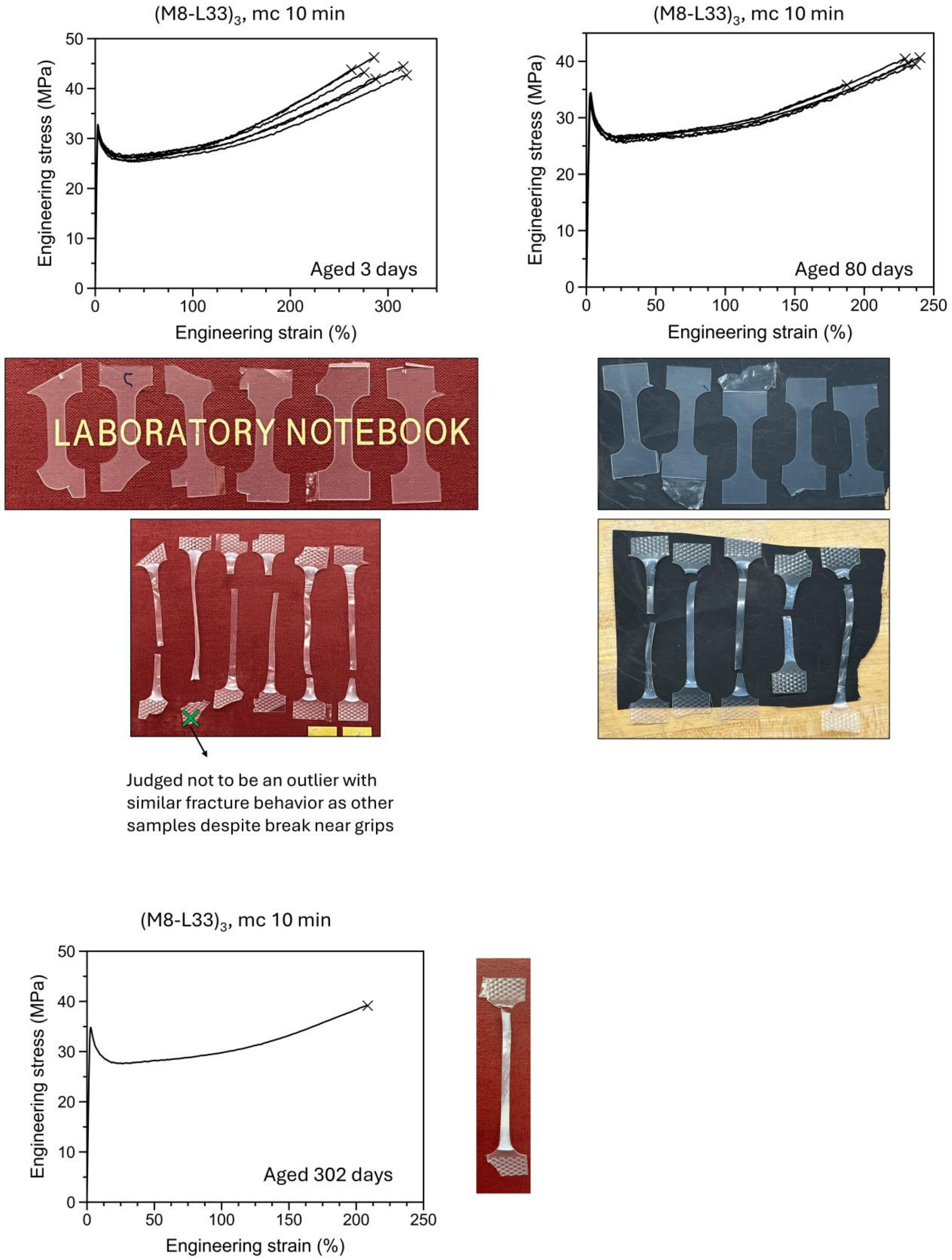

Figure S30. $(M8\text{-}L33)_3$ stress strain plots (melt crystallized 10 min) and sample images.

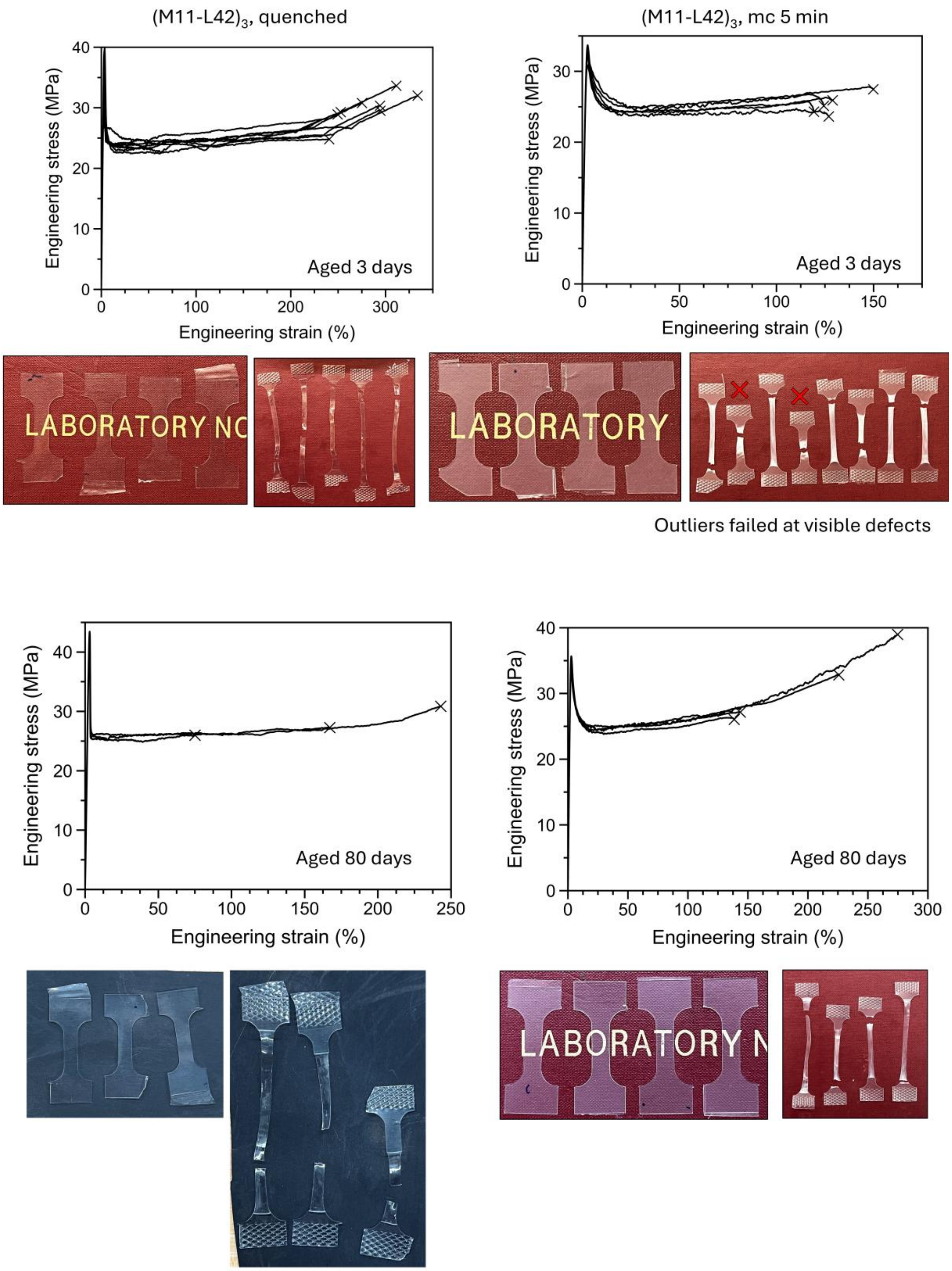

Figure S31. $(M11\text{-}L42)_3$ stress strain plots and sample images.

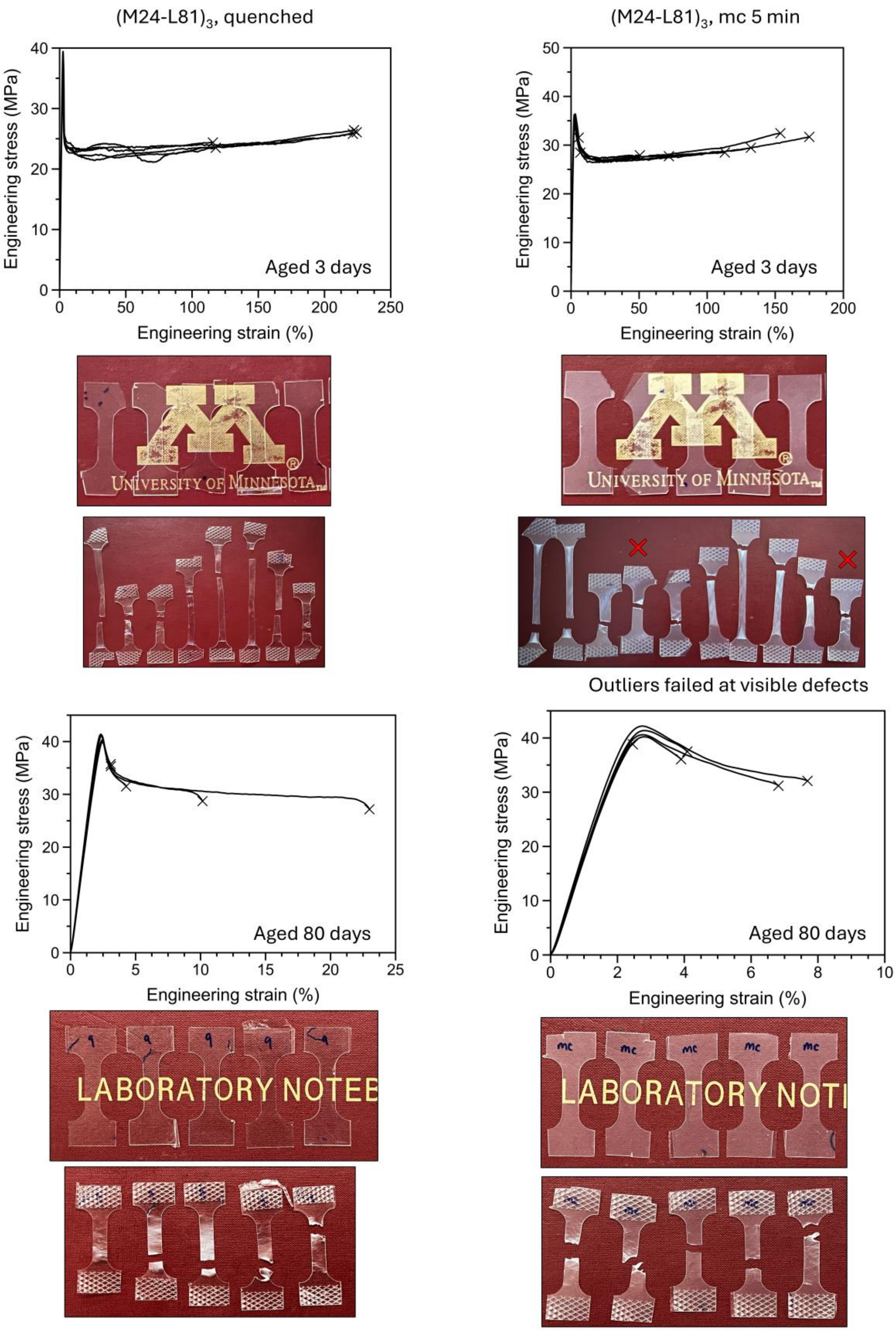

Figure S32. $(M24\text{-}L81)_3$ stress strain plots and sample images.

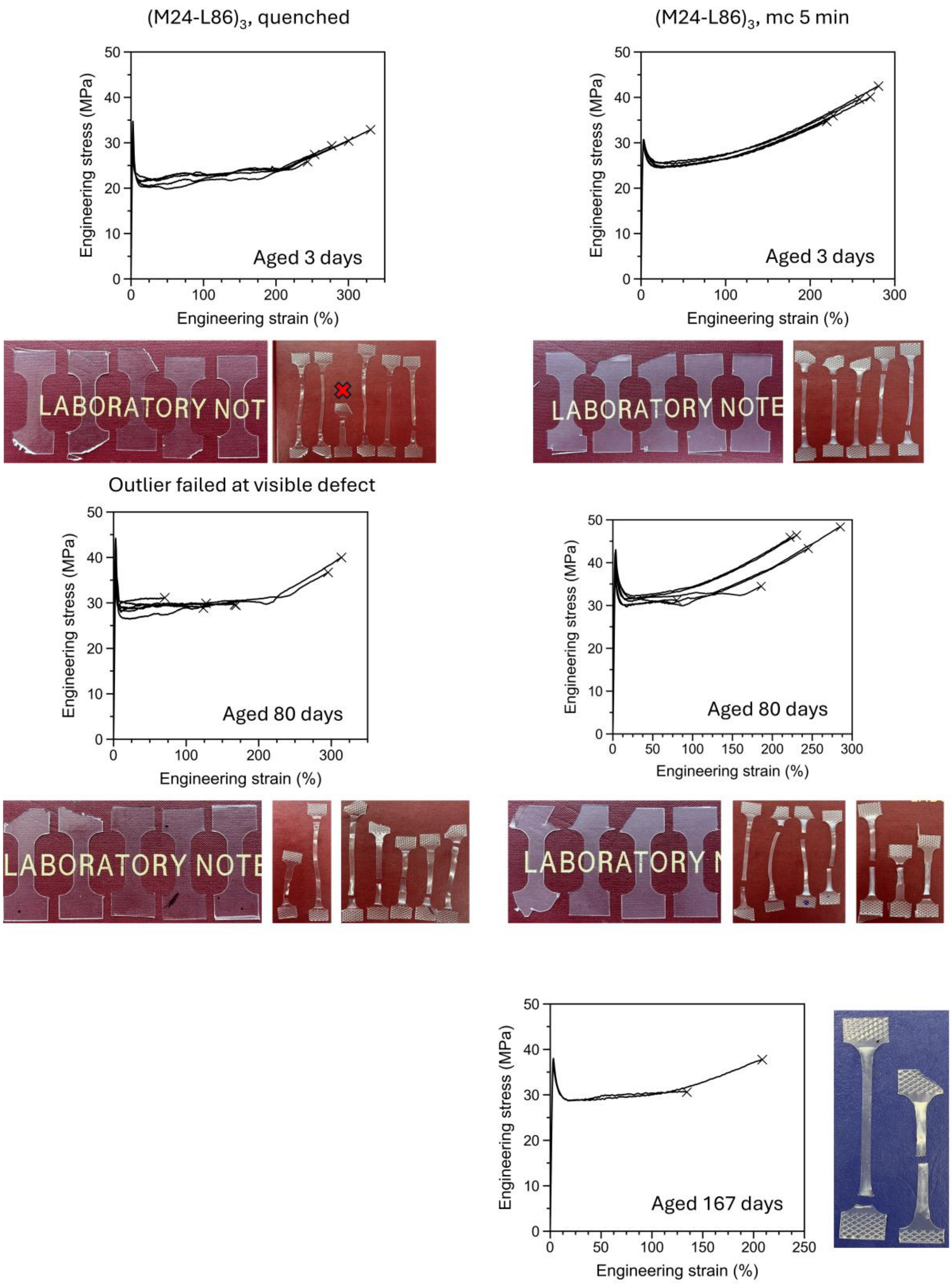

Figure S33. (M24-L86)$_3$ stress strain plots and sample images.

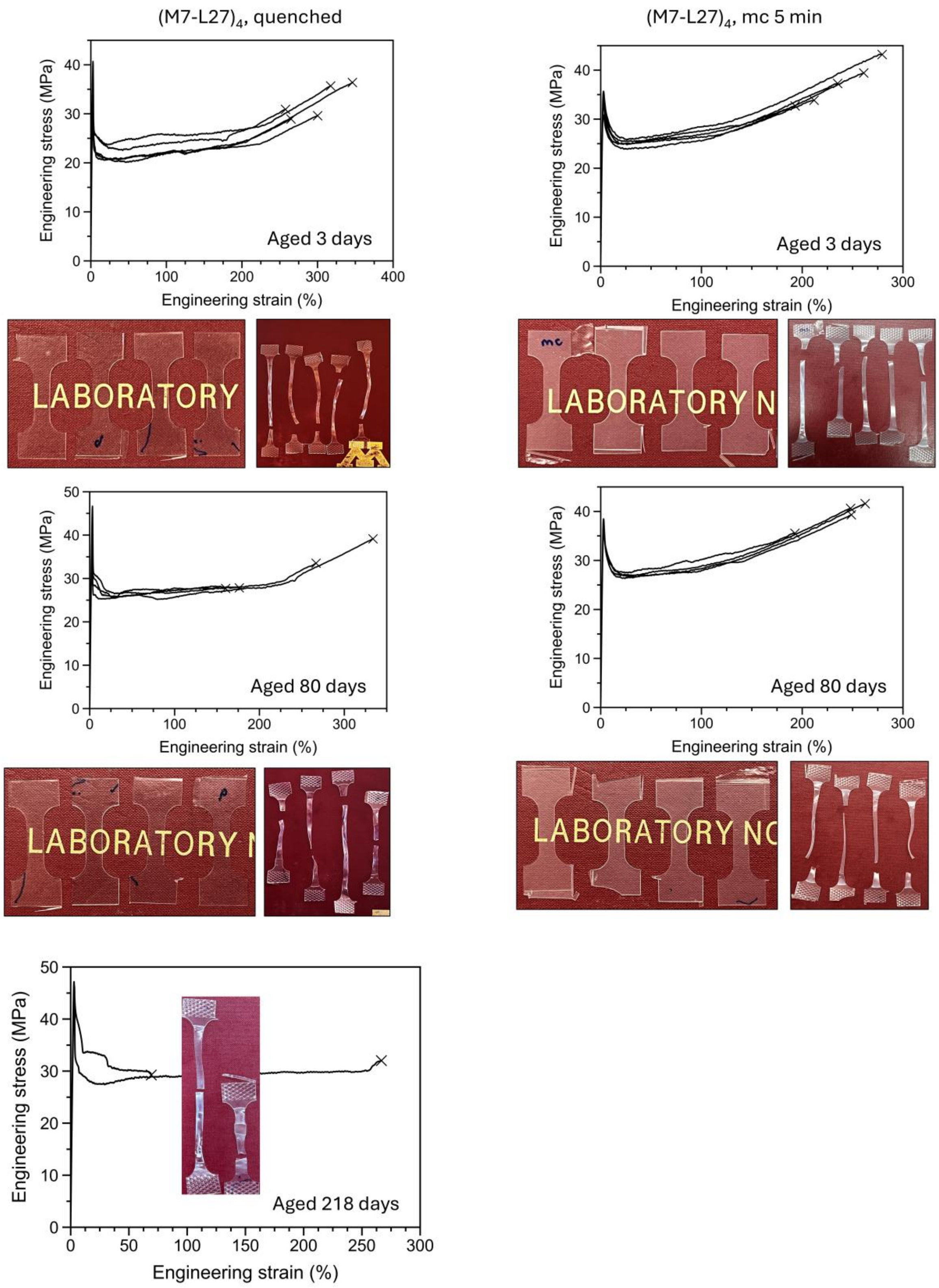

Figure S34. (M7-L27)$_4$ stress strain plots (quenched and melt crystallized 5 min) and sample images.

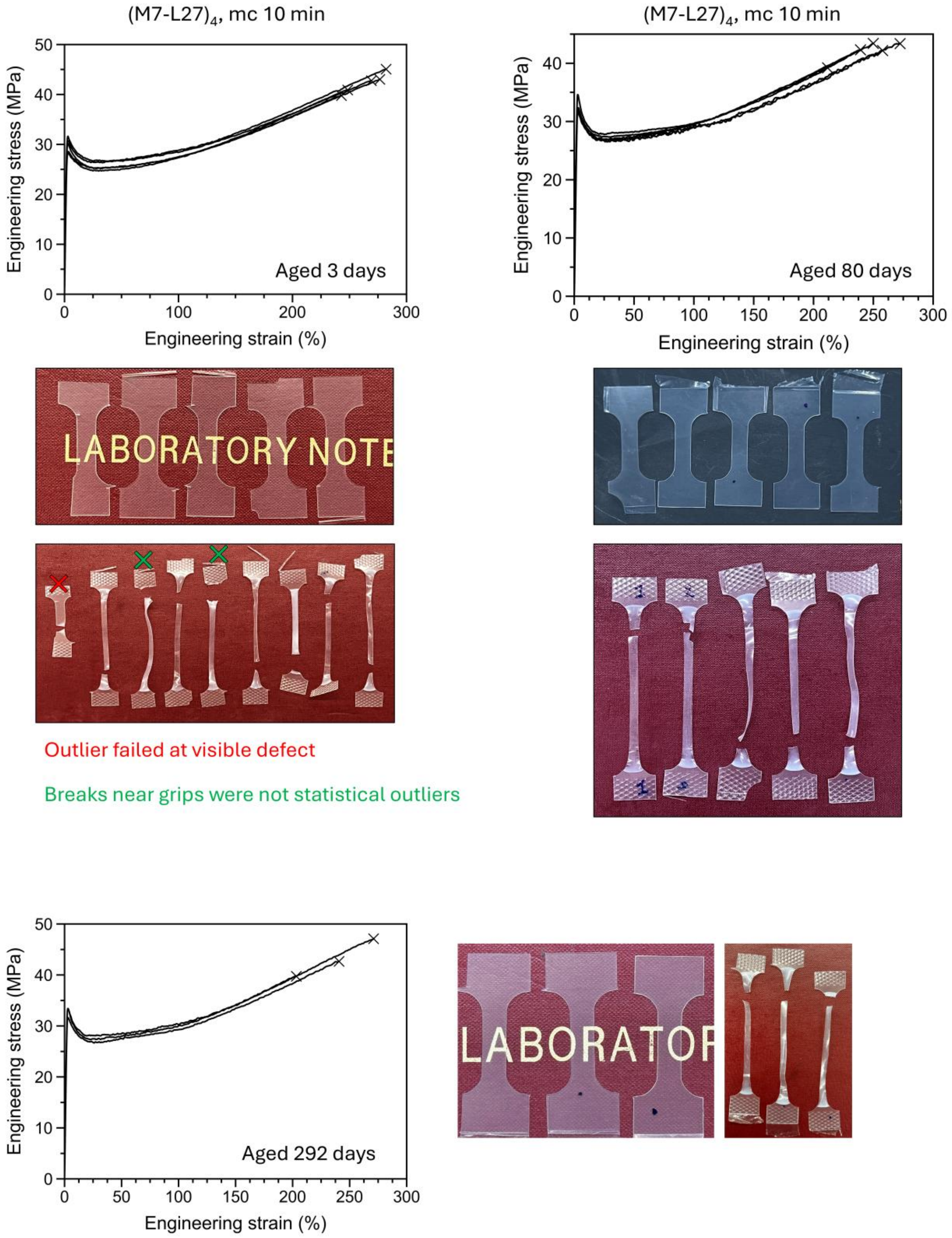

Figure S35. (M7-L27)$_4$ stress strain plots (melt crystallized 10 min) and sample images.

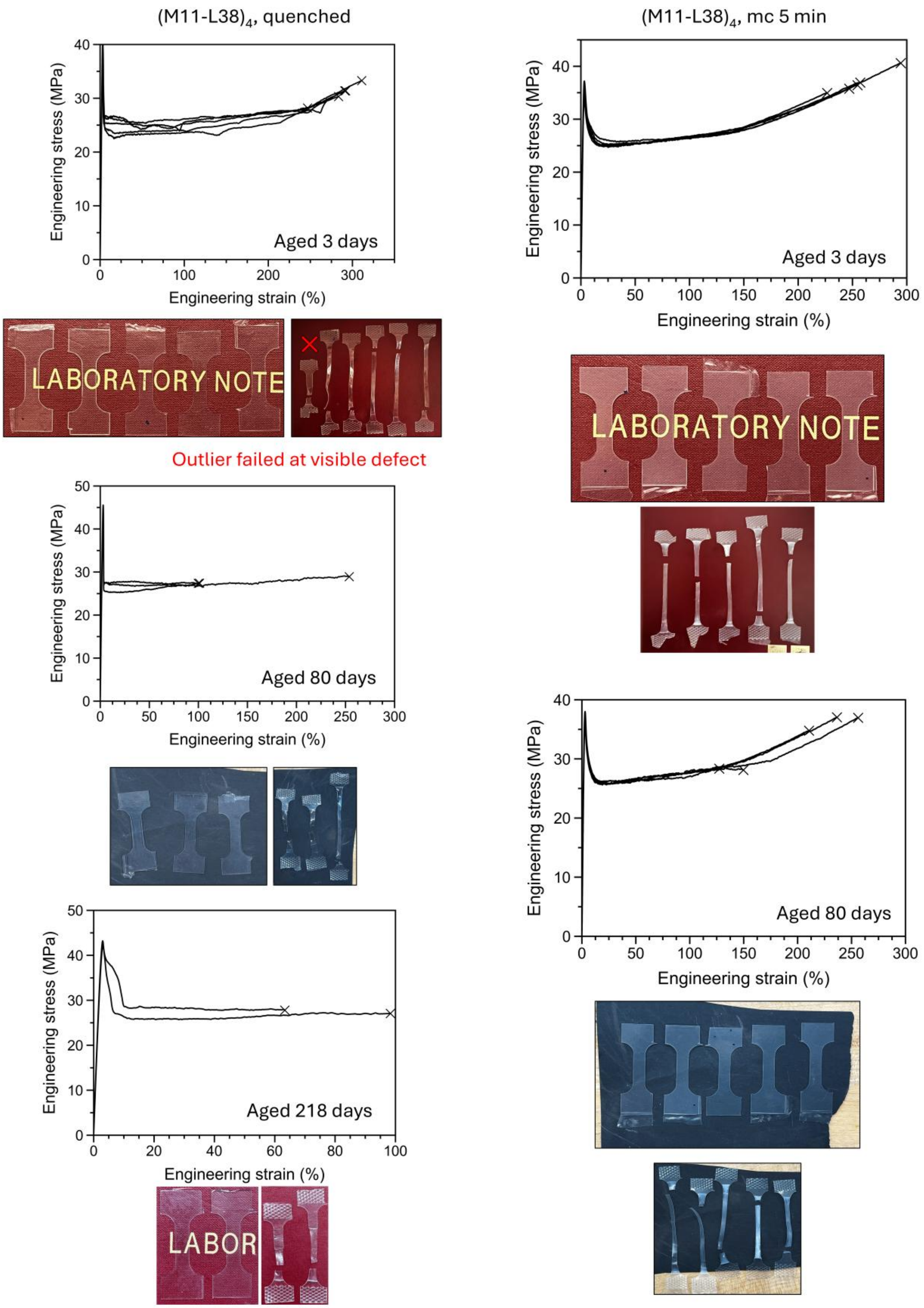

Figure S36. (M11-L38)$_4$ stress strain plots and sample images.

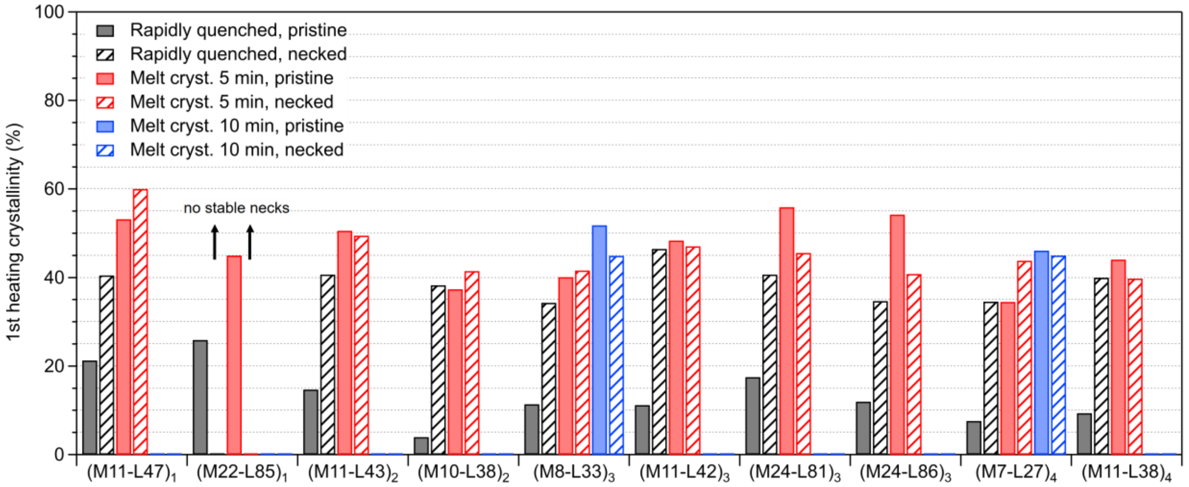

Figure S37. *Ex situ* DSC analysis of polymer films' 1st heating crystallinities. The large crystallinity increase after necking rapidly quenched specimens indicates significant strain-induced crystallization, while the retention or slight reduction in crystallinity of melt crystallized specimens indicates crystallite reconfiguration, both of which are consistent with our previous report on LML triblocks.[11]

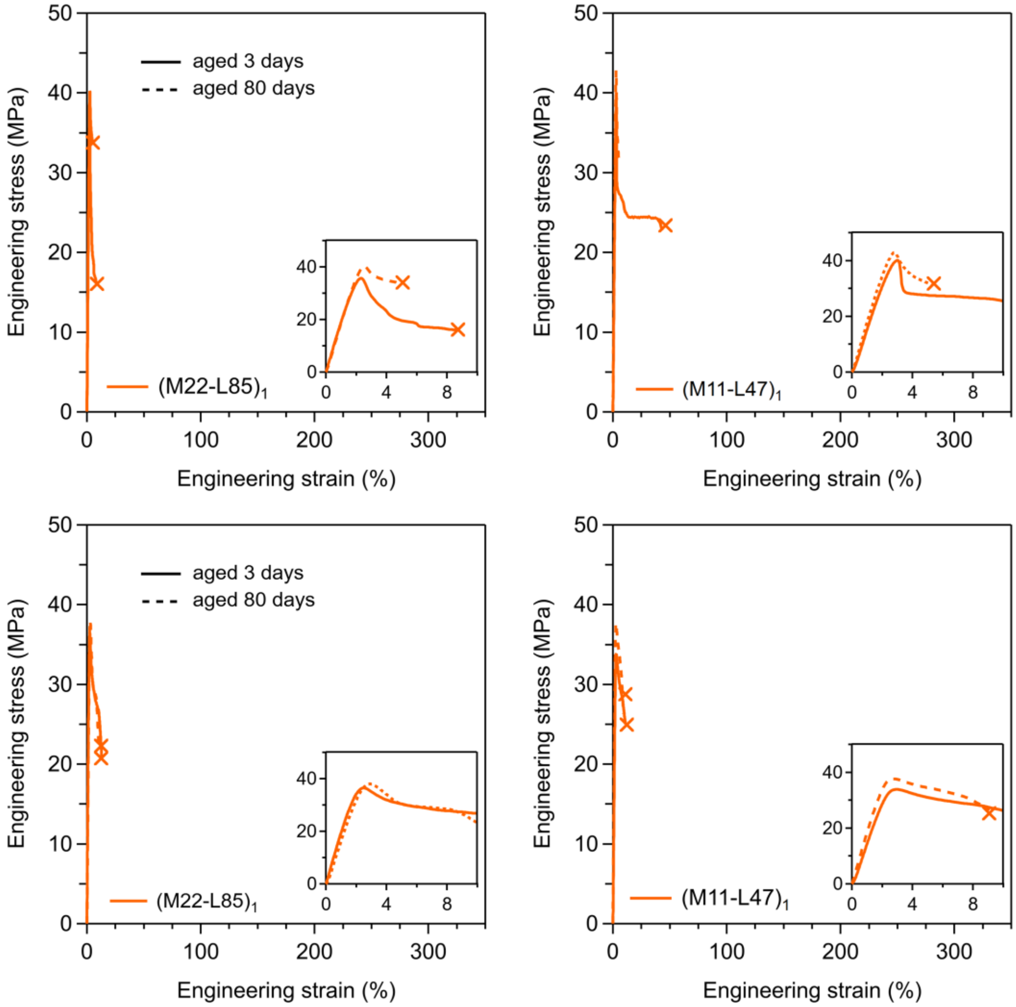

Figure S38. Representative tress-strain curves collected for diblocks (M22-L85)$_1$ (left) and (M11-L47)$_1$ (right). Curves from rapidly quenched results are in the top row, and those for melt crystallized specimens are in the bottom row.

The (ML)$_1$ diblock polymers exhibited much poorer toughness than the other (ML)$_n$ star-blocks with $n \geq 2$ and showed basically no meaningful mechanical longevity (**Figure S37**). This result is reminiscent of behavior in PS-rich PS-*b*-poly(*n*-butyl methacrylate) diblocks.[22,23] We speculate that in addition to the rubbery domain cavitation that can initiate crazing,[24] the dangling PγMCL blocks weakened the cohesion of the domains to the point they could not sustain the domain stretching that we observed supporting shear yielding in our previous study.[11]

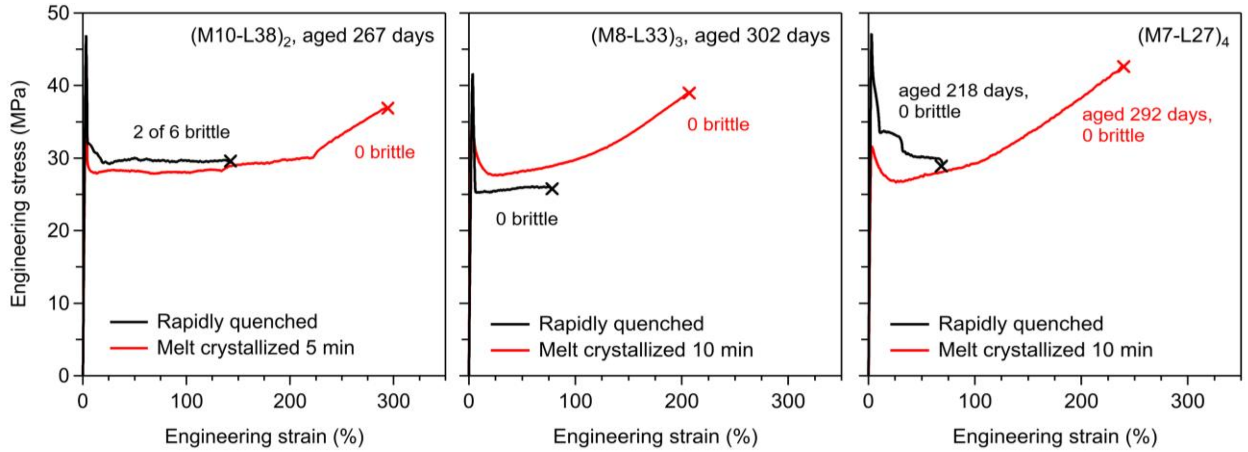

Figure S39. Stress-strain curves for star-blocks collected at aging times longer than 80 days.

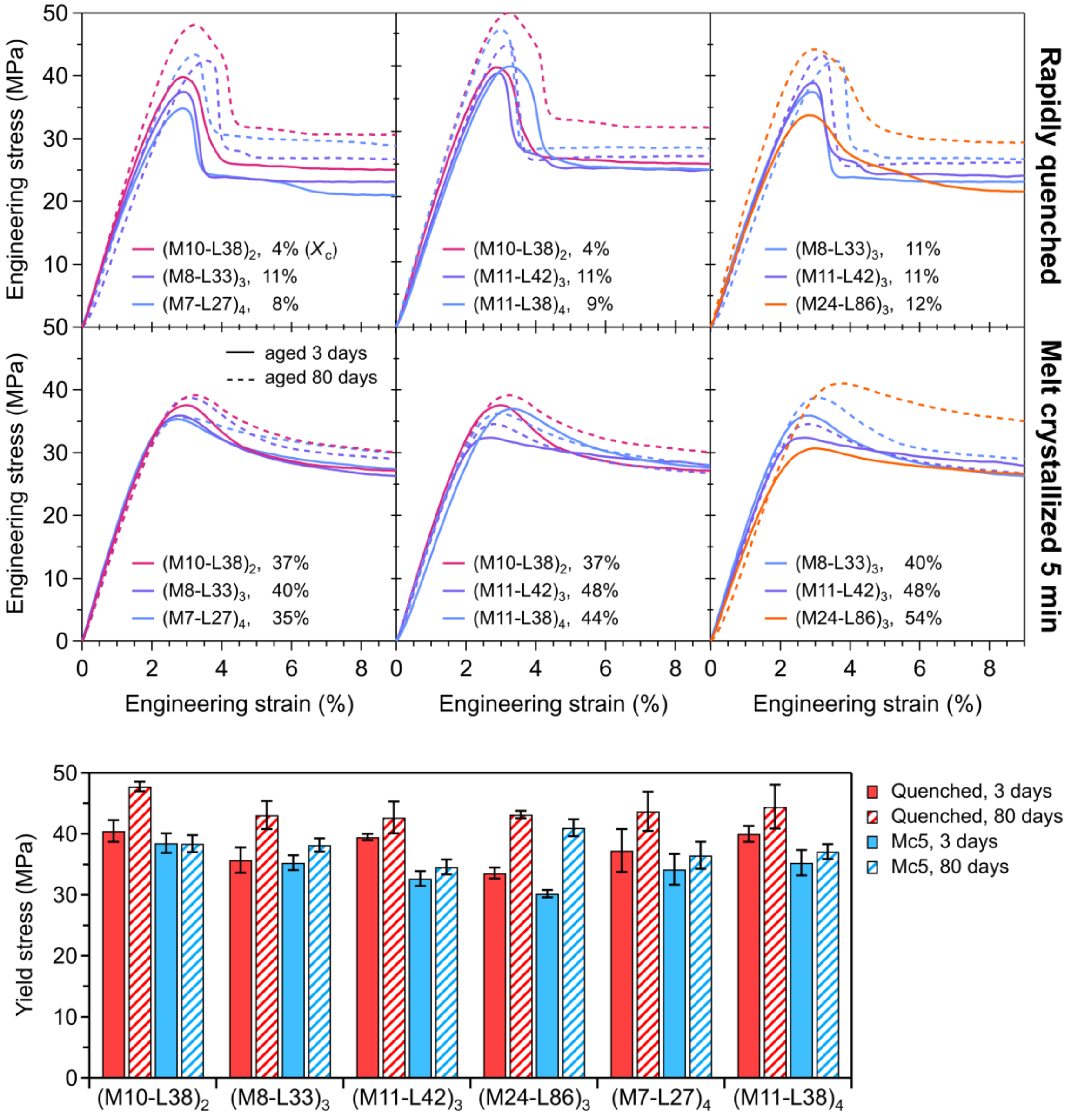

Figure S40. (top) Magnified yield regions of the stress strain curves and (bottom) bar chart of yield stresses for selected $(ML)_n$ star-blocks with $n > 1$ compared at different aging times and with different thermal histories. Error bars indicate 95% confidence interval widths.

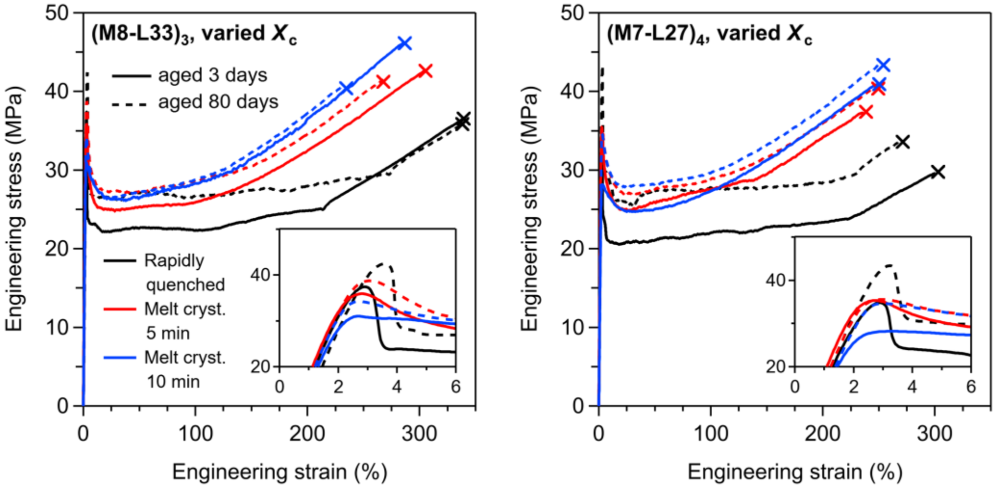

Figure S41. Stress-strain curves for (M8-L33)$_3$ (left) and (M7-L27)$_4$ (right) with different states of initial crystallinity.
(M8-L33)$_3$: quenched – X_c = 11%, melt cryst. 5 min – X_c = 40%, melt cryst. 10 min – X_c = 52%.
(M7-L27)$_4$: quenched – X_c = 7%, melt cryst. 5 min – X_c = 35%, melt cryst. 10 min – X_c = 46%.

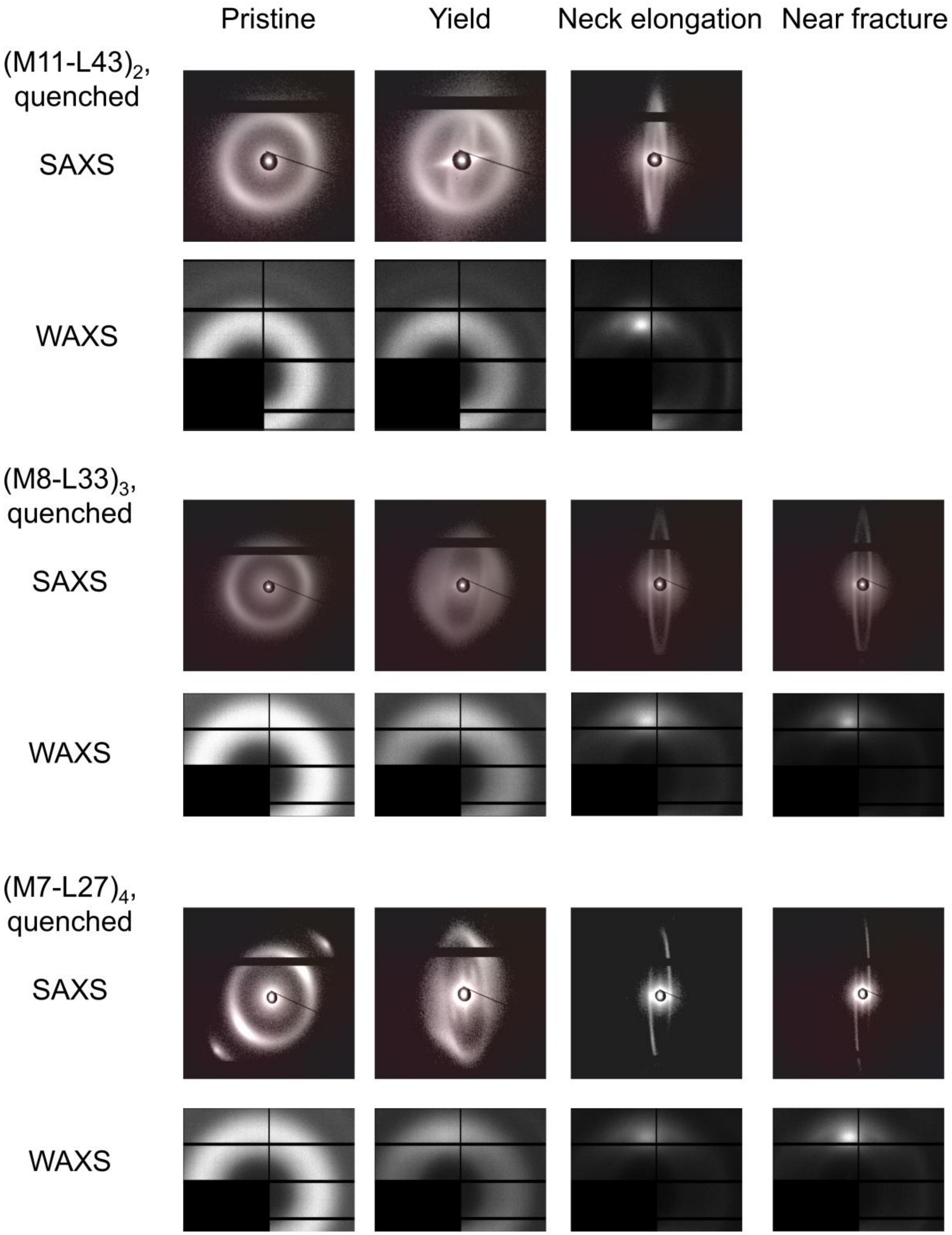

Figure S42. 2D X-ray scattering images captured during *in situ* tensile elongation of the fixed M_{tot} series in a rapidly quenched, low-crystallinity state. The features corresponding to sparse matrix voiding during yielding, PγMCL domain stretching during necking, strain-induced crystallization, and fibrillation at high strains are consistent with our previous report.[11] The strain direction is horizontal for all images.

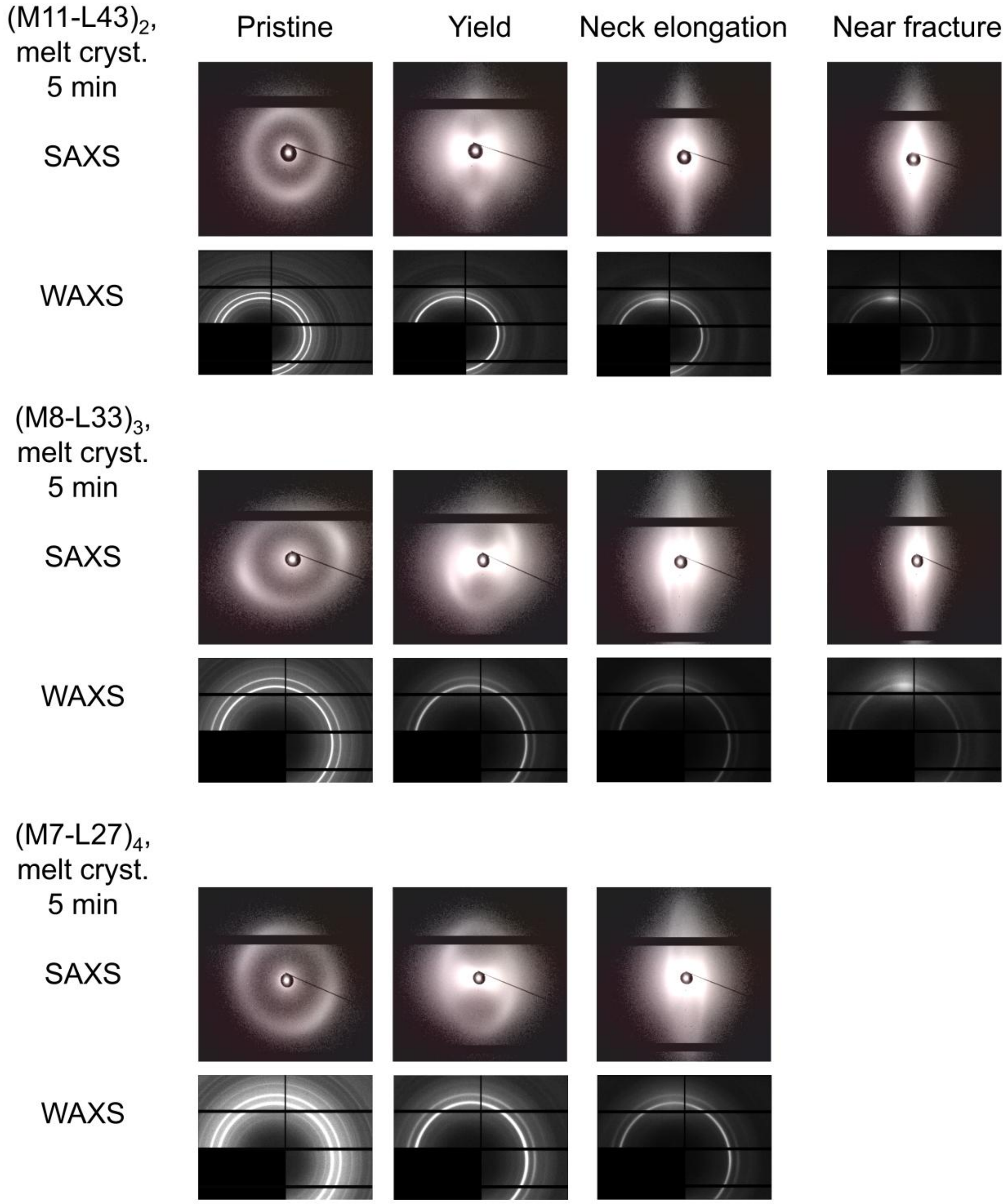

Figure S43. 2D X-ray scattering images captured during *in situ* tensile elongation of the fixed M_{tot} series after 5 min of melt crystallization at 100 °C. The features corresponding to coarse lamellar slip at yielding, PγMCL domain stretching during necking, and lamellar-to-fibrillar PLLA crystallite transition are consistent with our previous report.[11] The strain direction is horizontal for all images.

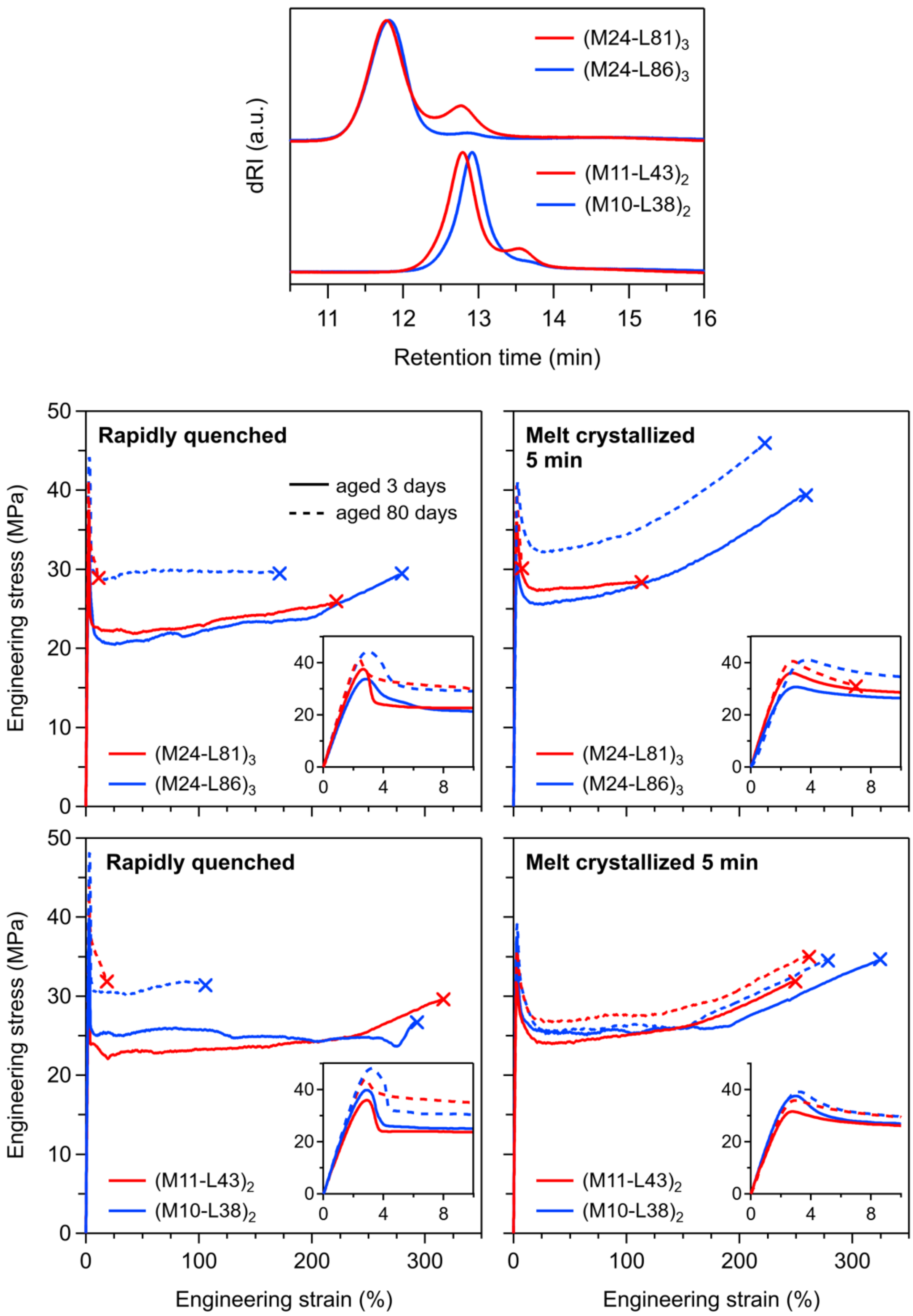

Figure S44. Comparison of (top) SEC traces and (bottom panels) stress-strain curves for less pure molecules $(M11\text{-}L43)_2$ and $(M24\text{-}L81)_3$ and their purer second iterations $(M10\text{-}L38)_2$ and $(M24\text{-}L86)_3$.

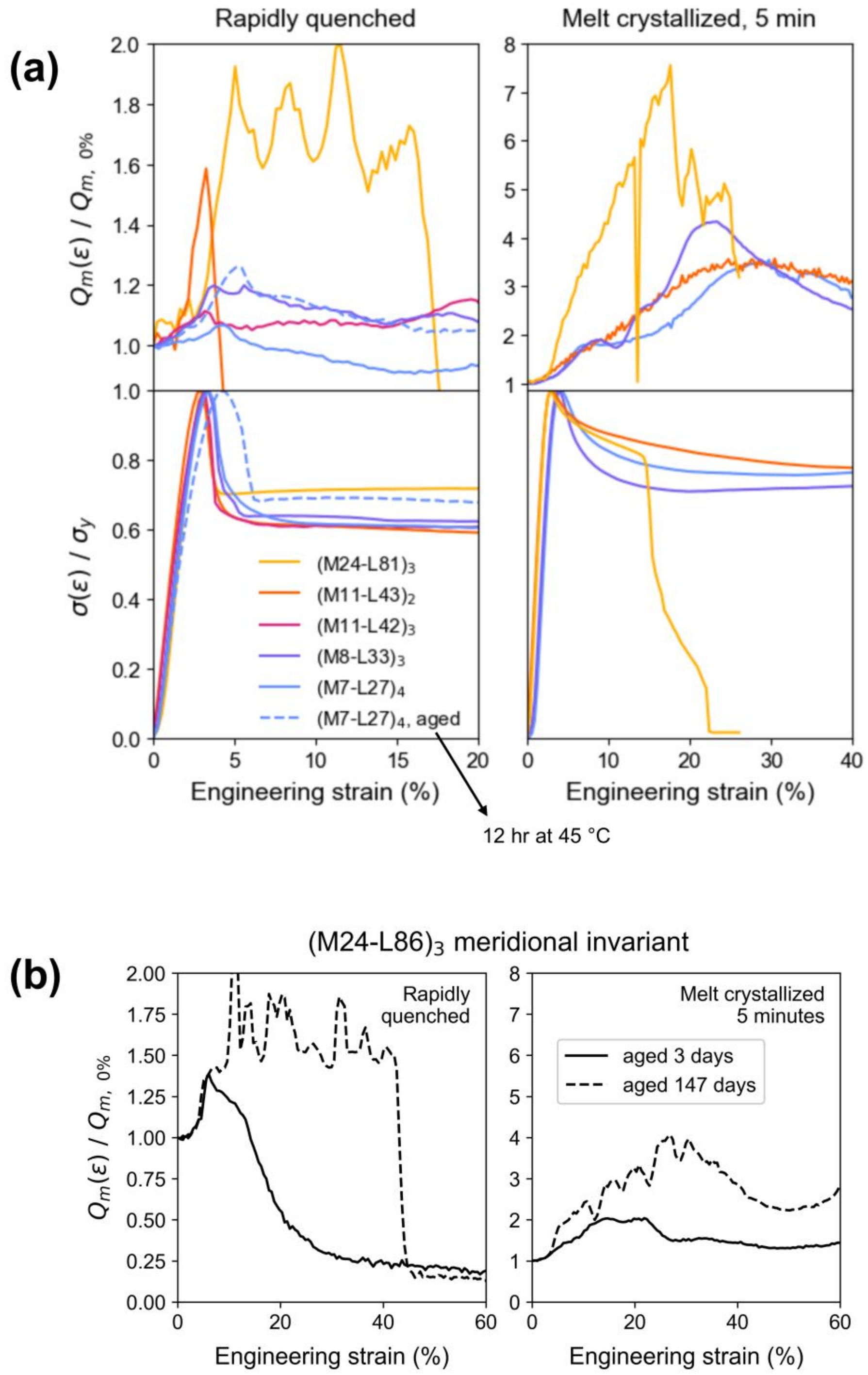

Figure S45. Meridional scattering invariant normalized to the value at 0% strain plotted as a function of engineering strain. Abrupt decreases in the invariant indicate the neck traversing the beam, as the thinner neck scatters less than the thicker pristine film. While the less pure macromolecules $(M11\text{-}L43)_2$ and $(M24\text{-}L81)_3$ show values ranging from 1.6–2.0 ahead of the neck front at three days of aging, $(M24\text{-}L86)_3$ has less voiding (~1.4) at three days and shows a similar amount of voiding only after 147 days.

Macromolecular purity strongly influenced both toughness and longevity. **Figure S44** superposes the SEC traces of $(M11\text{-}L43)_2^*$ and $(M24\text{-}L81)_3^*$ and their purer second iterations $(M10\text{-}L38)_2$ and $(M24\text{-}L86)_3$ and compares their tensile behavior. $(M11\text{-}L43)_2^*$ fully transitioned to brittle crazing after 80 days in the low-crystallinity state, while $(M24\text{-}L81)_3^*$ embrittled irrespectively of crystallinity. With lower contents of low-molar mass diblock and PLLA homopolymer impurities,[11] $(M10\text{-}L38)_2$ and $(M24\text{-}L86)_3$ had dramatically higher toughness retention. *In situ* tensile SAXS analysis revealed that the poorer toughness of $(M11\text{-}L43)_2^*$ and $(M24\text{-}L81)_3^*$ was associated with more prevalent matrix voiding, signaling a higher incidence of crazing (**Figure S45**). These findings showcase the parasitic effect of untethered (PLLA) or unanchored (diblocks) chains that cannot effectively transmit stress to the PγMCL domains and instead promote the strain localization necessary for craze formation, mimicking an advanced physical age.[25]

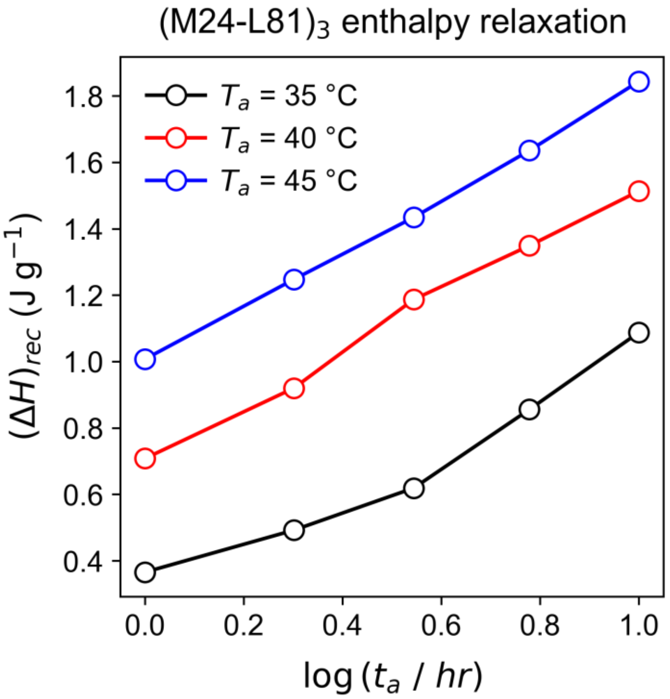

Figure S46. Enthalpy recovery as a function of aging time for $(M24\text{-}L81)_3$ annealed for up to 10 hours at various temperatures (T_a) below $T_{g,\ PLLA}$. Solid lines are guides for the eye, not fits. We used these data to select T_a = 45 °C for the isothermal annealing studies, as this maximized the enthalpy recovery and provided the greatest "signal-to-noise" ratio of heat flow through the glass transition.

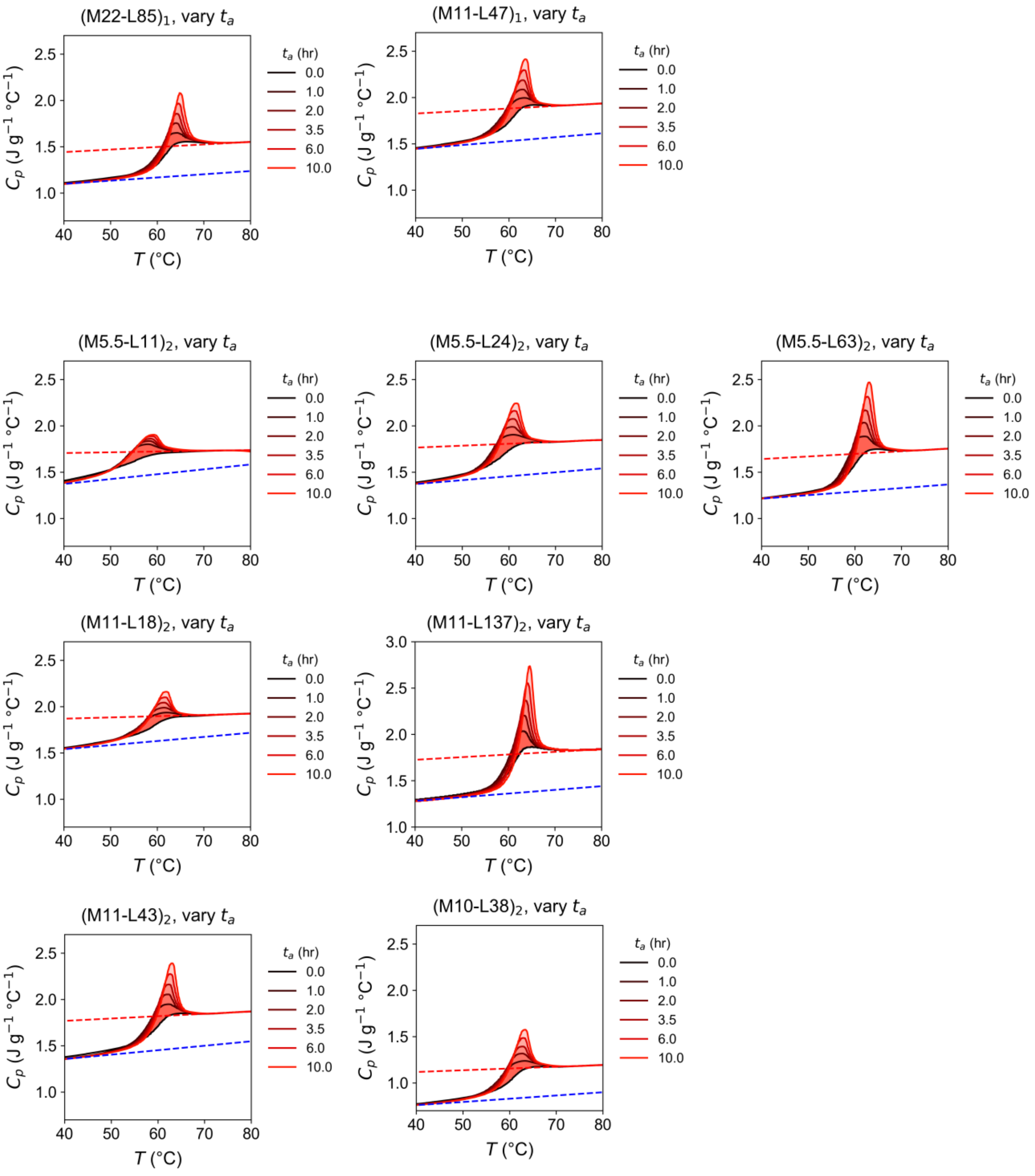

Figure S47. Heat capacity recorded on reheating during varied t_a aging studies with T_a = 45 °C for diblocks and triblocks. All triblocks have been assimilated with the nomenclature of the current study. Note the scale adjustment necessary for $(M11\text{-}L137)_2$.

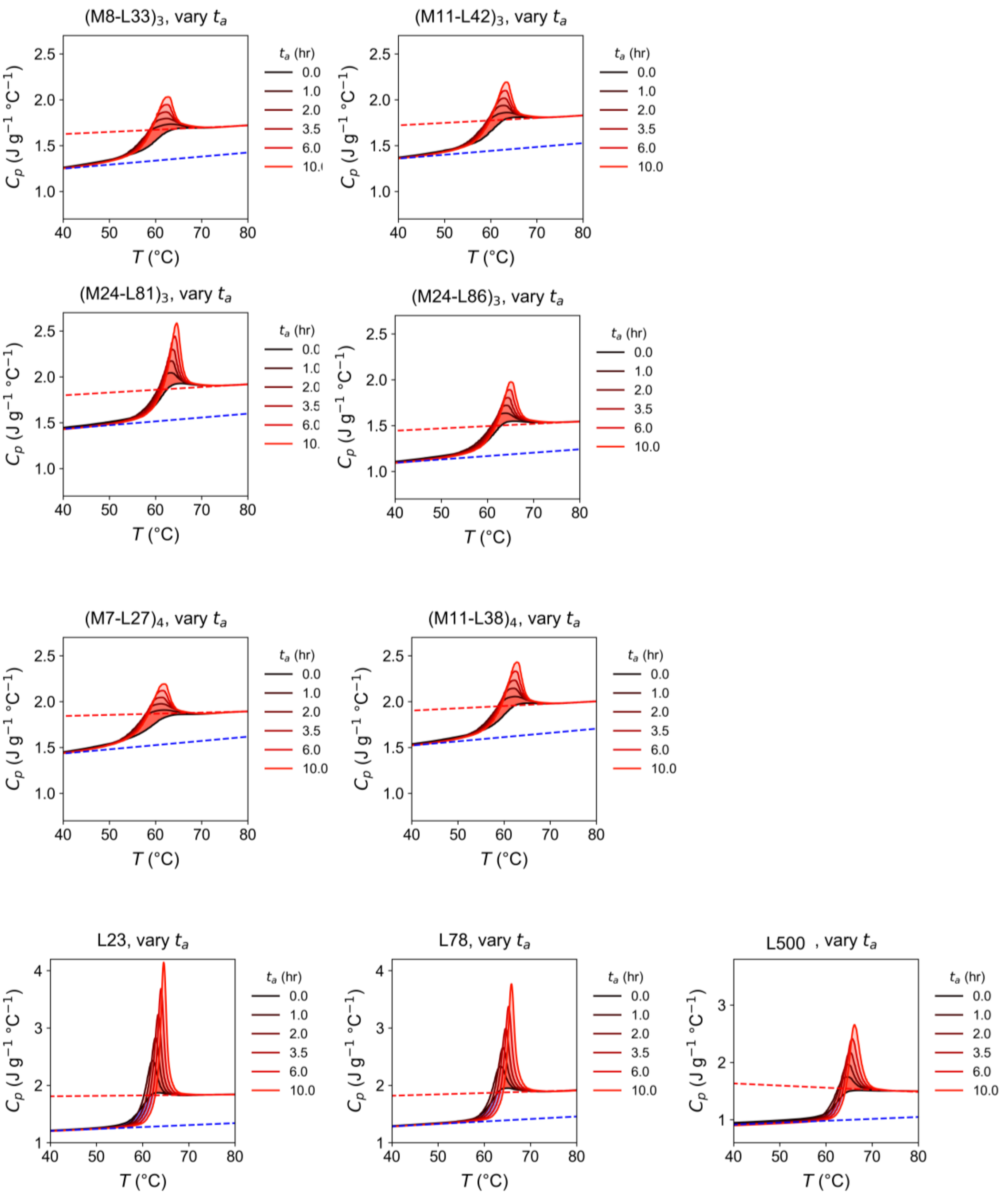

Figure S47, continued. Heat capacity recorded on reheating during varied t_a aging studies with T_a = 45 °C for three-arm star-blocks, four-arm star-blocks, and PLLA homopolymers. Note the scale adjustments necessary for the homopolymers.

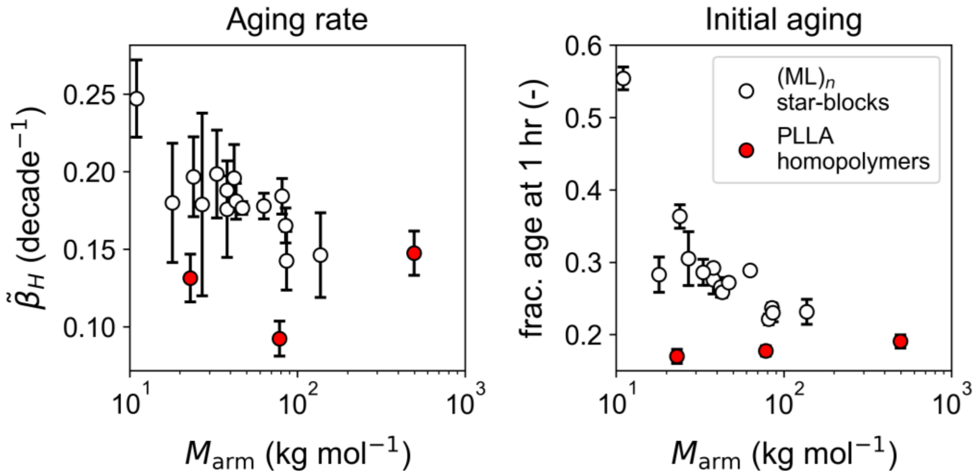

Figure S48. Fractional aging rates (fit slopes) (left) and initial fractional age (fit intercepts) (right) obtained from isothermal aging studies at 45 °C plotted against M_{arm}. These data include LML triblocks from our previous study: LML 11-11-11, LML 24-11-24, LML 63-11-63, LML 18-22-18, and LML 137-22-137. Error bars denote 95% confidence intervals.

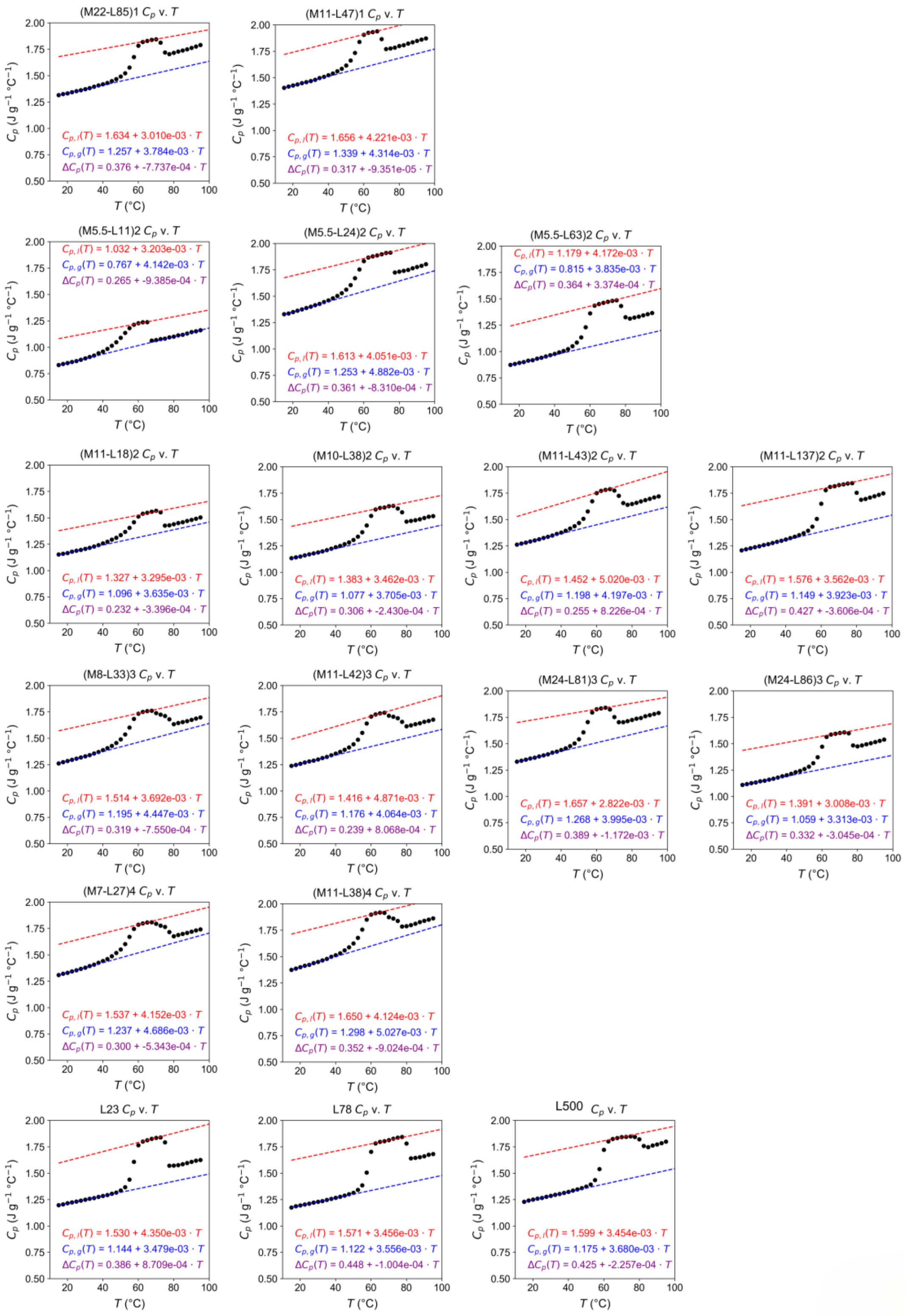

Figure S49. $C_p(T)$ obtained for all materials using the quasi-isothermal modulated DSC protocol. The fits to glassy and liquid regimes are shown with blue and red dashed lines, respectively. The abrupt decrease in $C_p(T)$ above $T_{\mathrm{g,\,PLLA}}$ is due to cold crystallization, and we took care to avoid this region in the liquid regime fit.

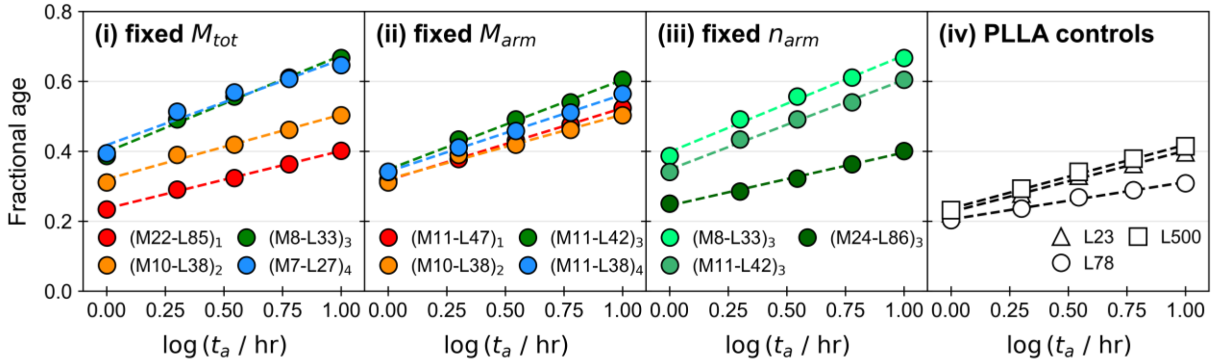

Figure S50. Fractional physical age plotted against logarithm of aging time with $(\Delta H)_{rec,\infty}$ calculated using $\Delta C_p(T)$ determined from quasi-isothermal modulated DSC experiments (**Figure S49** above) rather than direct fitting of the glass and liquid C_p traces above and below $T_{g,\text{ PLLA}}$ as in the main text. The trends are in qualitative agreement, though the values of fractional age are systematically higher with this method than those shown in the main text.

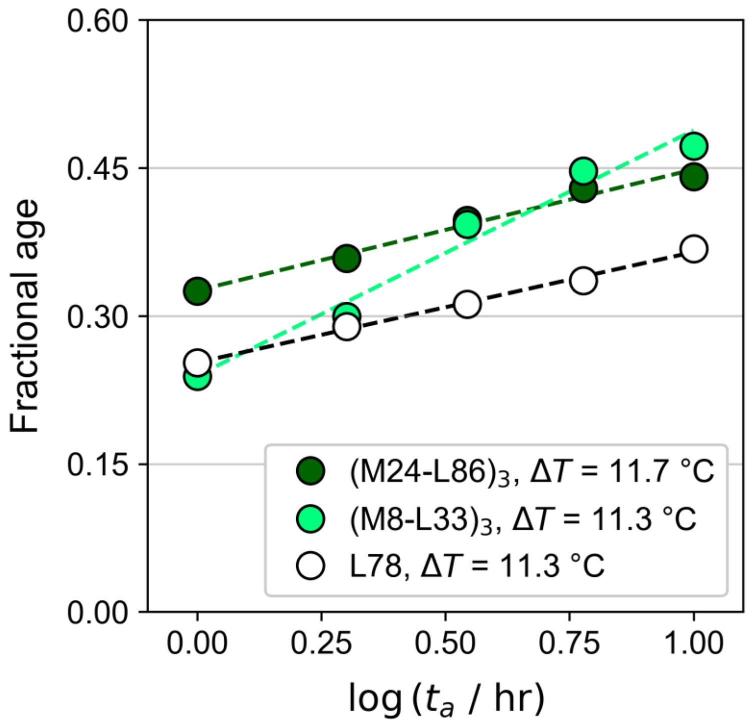

Figure S51. Enthalpy relaxation study with fixed subcooling $\Delta T = T_{g,\text{ PLLA}} - T_a$, based on the $T_{g,\text{ PLLA}}$ values determined from quasi-isothermal DSC. (M8-L33)$_3$: $T_{g,\text{ PLLA}}$ = 54.04 °C, T_a = 42.75 °C. (M24-L86)$_3$: $T_{g,\text{ PLLA}}$ = 58.50 °C, T_a = 46.79 °C. L78: $T_{g,\text{ PLLA}}$ = 58.12 °C, T_a = 46.79 °C.

To examine the effect of the minor differences in subcooling $T_{g, PLLA} - T_a$ caused by the slight scatter of $T_{g, PLLA}$ values (**Figure S15b**), we studied the aging of (M8-L33)$_3$, (M24-L86)$_3$, and L78 at a fixed subcooling of ~11.5 °C (**Figure S51**). Here, (M24-L86)$_3$ showed the highest initial age and aged at the same rate as L78, while (M8-L33)$_3$ had a similar initial age as L78 but aged most rapidly, and so the star-blocks still attain higher physical ages than PLLA at fixed subcooling.

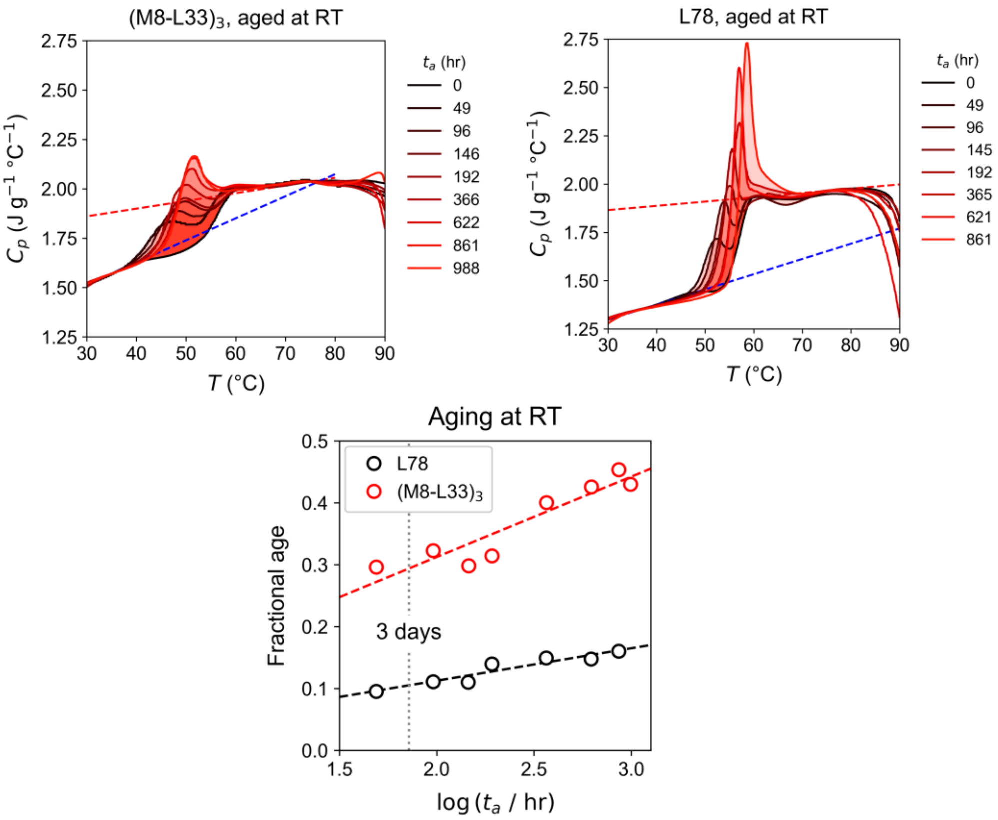

Figure S52. (top) Reheating curves for (M8-L33)$_3$ and L78 throughout room temperature aging following rapid quenching. (bottom) Resultant fractional ages against aging time. The three-day timepoint is marked to show the first mechanical evaluation, at which point L78 is brittle[11] while (M8-L33)$_3$ is highly tough. Note: to minimize the error associated with uncertainty in sample masses, each C_p trace was scaled and shifted to align the values at 37.5 and 75 °C with those of the t_a = 0 hr trace. This is appropriate considering that the w_{PLLA} of each material is consistent across the aging times, and the area normalization in the fractional age calculation removes any dependence on heat capacity scaling.

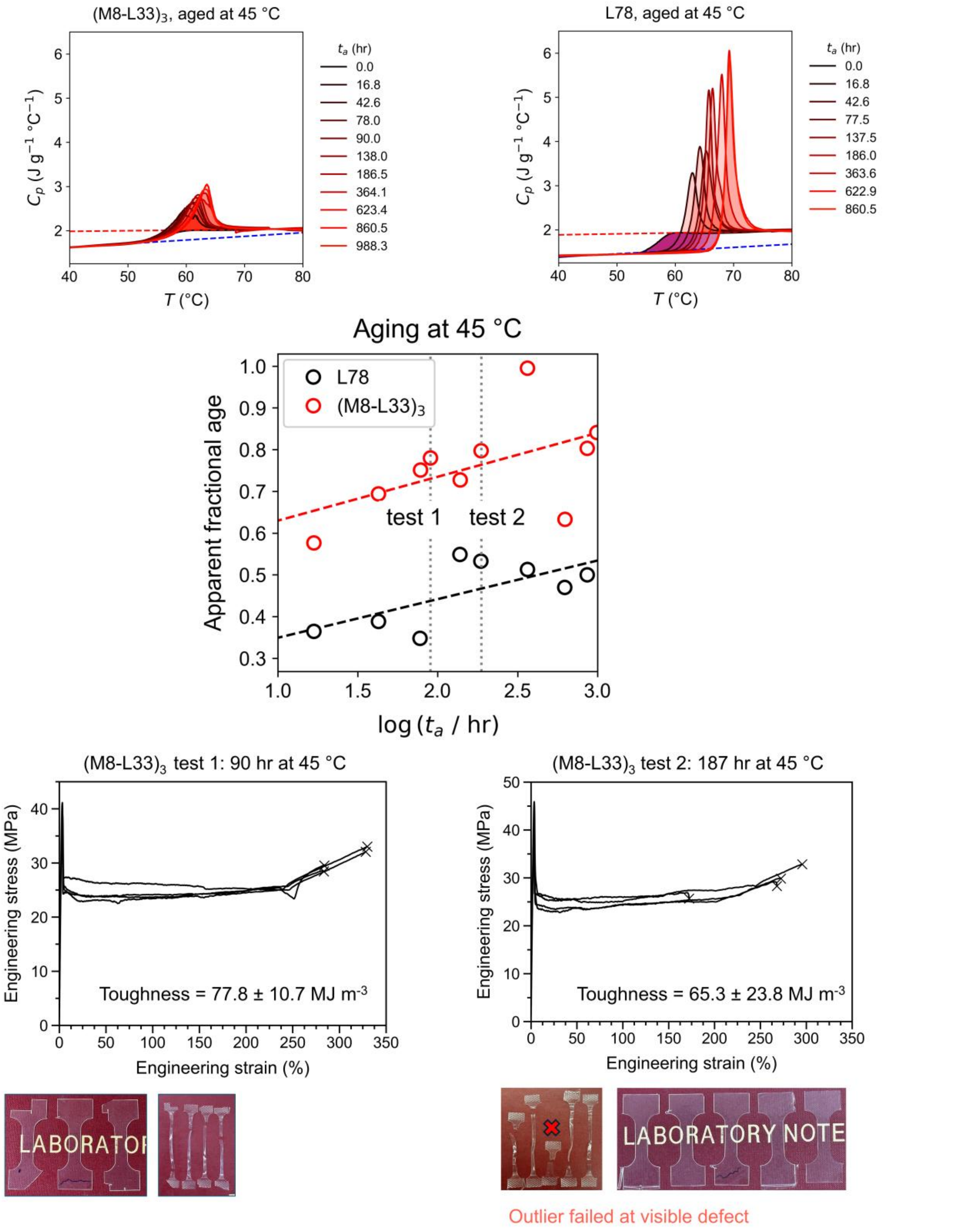

Figure S53. (top) Reheating curves for (M8-L33)$_3$ and L78 throughout room temperature aging following rapid quenching. (middle) Resultant fractional ages against aging time. Trace shifts were performed as described in the caption of **Figure S52** above.

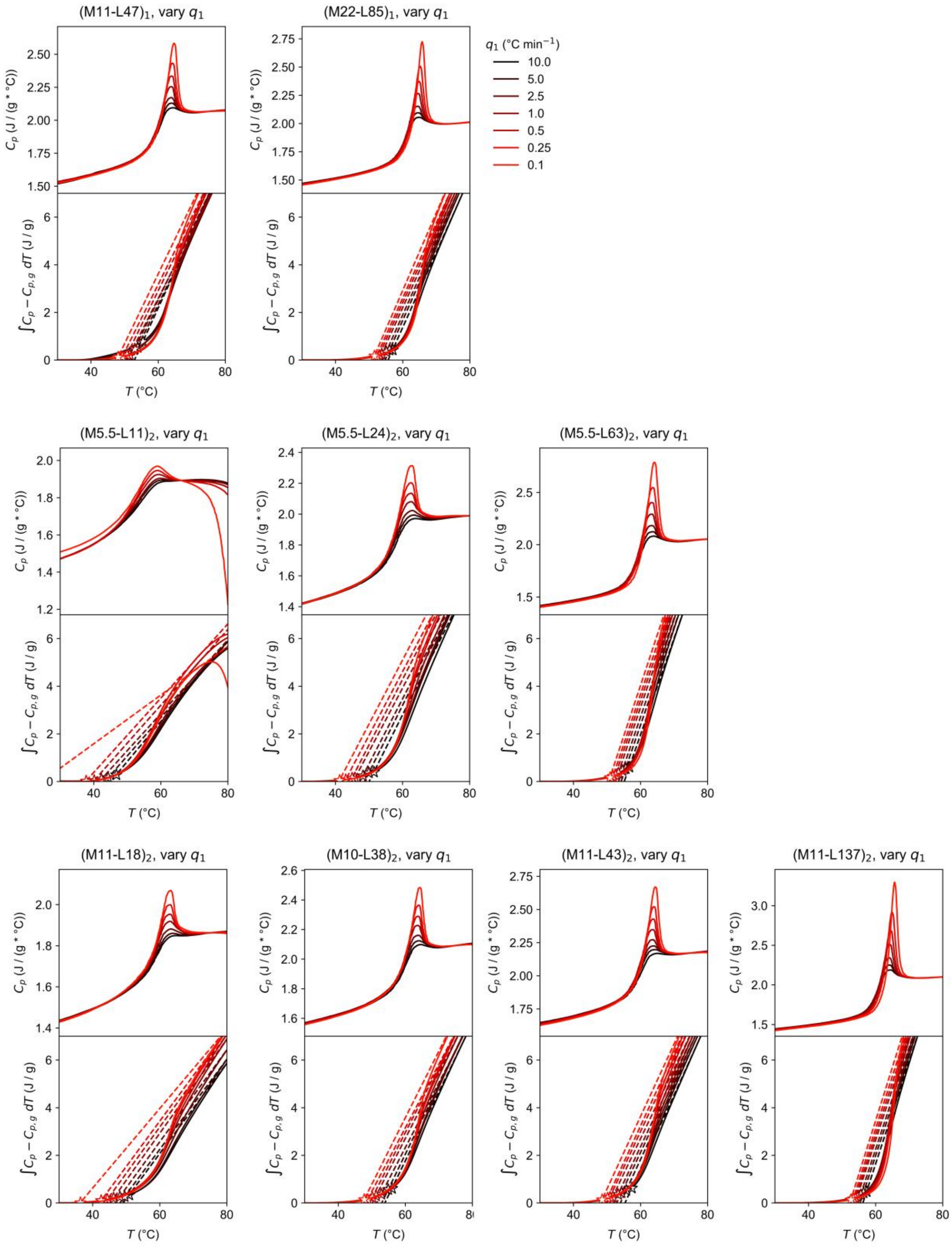

Figure S54. Varied cooling rate results for diblocks and triblocks. (top panel) Reheating curves after cooling through $T_{g,\,\mathrm{PLLA}}$ at the indicated rates. (Bottom panel) Excess enthalpy, or the integral of $C_p(T) - C_{p,g}(T)$ with respect to temperature, showing the extrapolations backward from liquid regime fits and self-intersections at $T_{f,\,\mathrm{lim}}$ marked by stars.

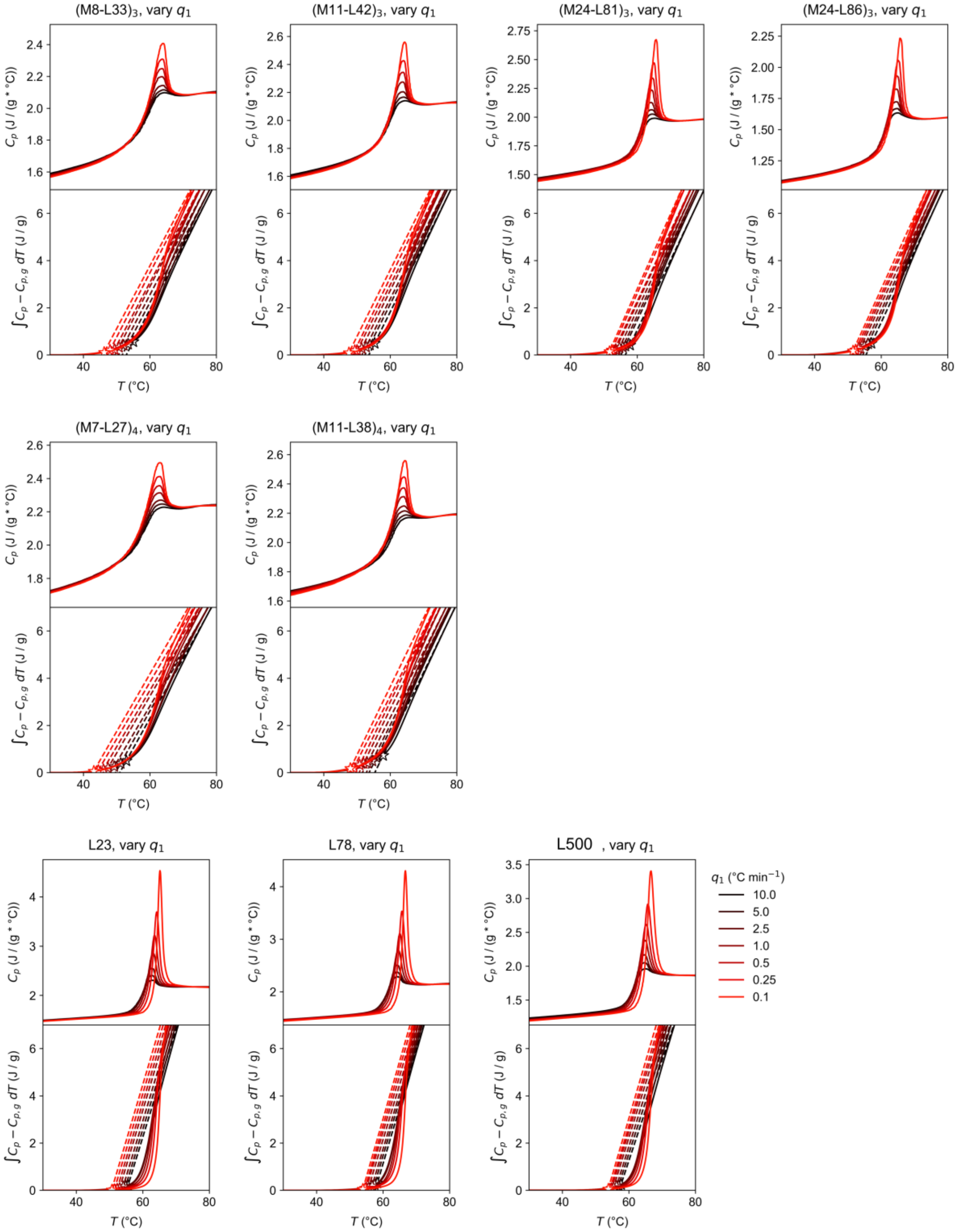

Figure S54, continued. Varied cooling rate results for three-arm star-blocks, four-arm star-blocks, and PLLA homopolymers. (top panel) Reheating curves after cooling through $T_{g,\ \mathrm{PLLA}}$ at the indicated rates. (Bottom panel) Excess enthalpy, or the integral of $C_p(T) - C_{p,g}(T)$ with respect to temperature, showing the extrapolations backward from liquid regime fits and self-intersections at $T_{f,\ \mathrm{lim}}$ marked by stars.

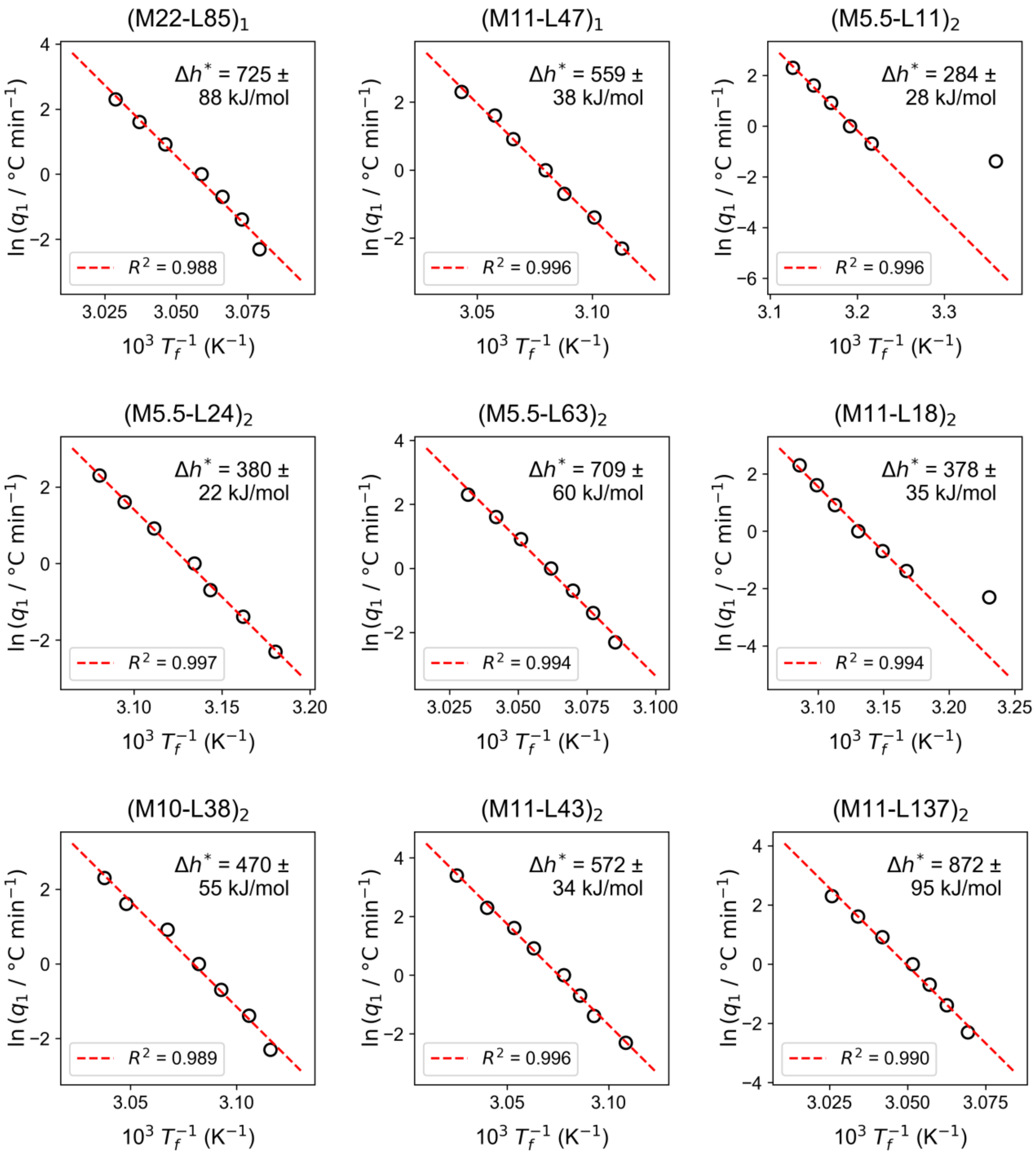

Figure S55. Plots of ln (q_1) v. $T_{f,\mathrm{lim}}^{-1}$ and linear fits, showing the resultant activation energies derived from the fit slopes, for diblocks and triblocks. "±" denotes a 95% confidence interval calculated with a student's *t*-statistic.

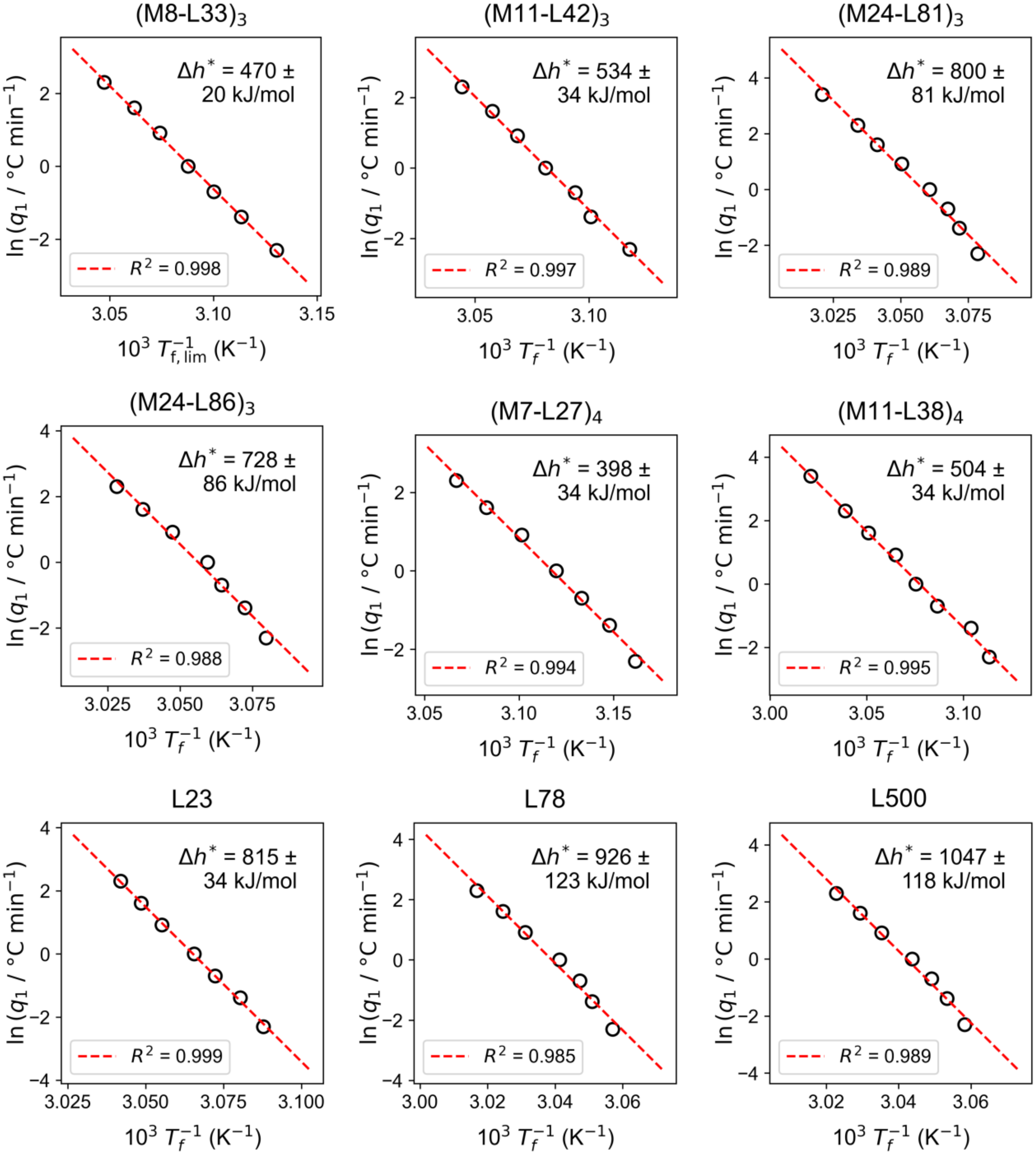

Figure S55, continued. Plots of ln (q_1) v. $T_{f,\lim}^{-1}$ and linear fits, showing the resultant activation energies derived from the fit slopes, for three-arm star-blocks, four-arm star-blocks, and PLLA homopolymers. "±" denotes a 95% confidence interval calculated with a student's *t*-statistic.

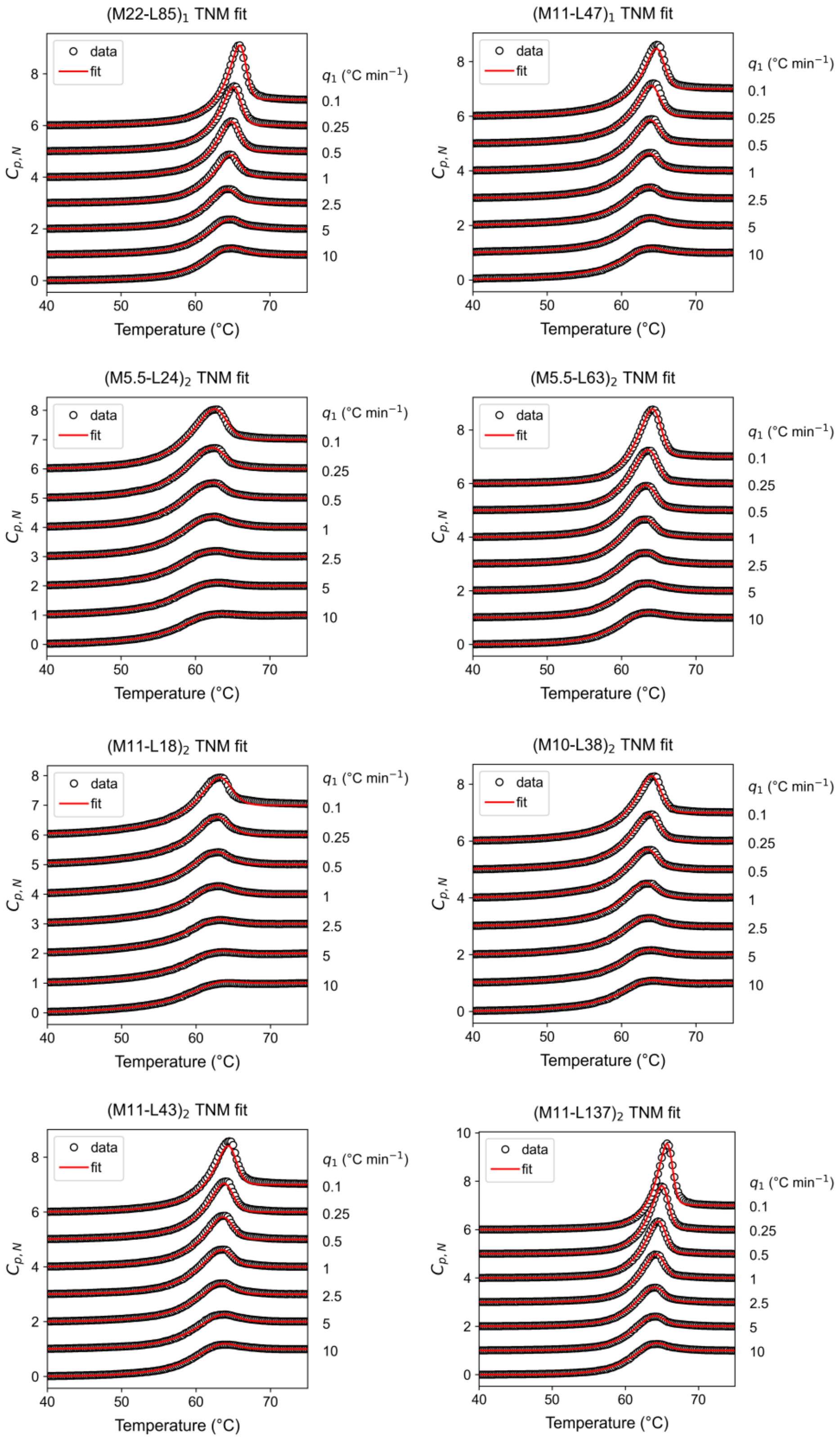

Figure S56. Varied q_1 data overlaid with fits to the TNM model for diblocks and triblocks. The data are presented as normalized heat capacity: $C_{p,N} = (C_p(T) - C_{p,g}(T)) / (C_{p,l}(T) - C_{p,g}(T))$. The traces are offset by one $C_{p,N}$ unit for visual clarity.

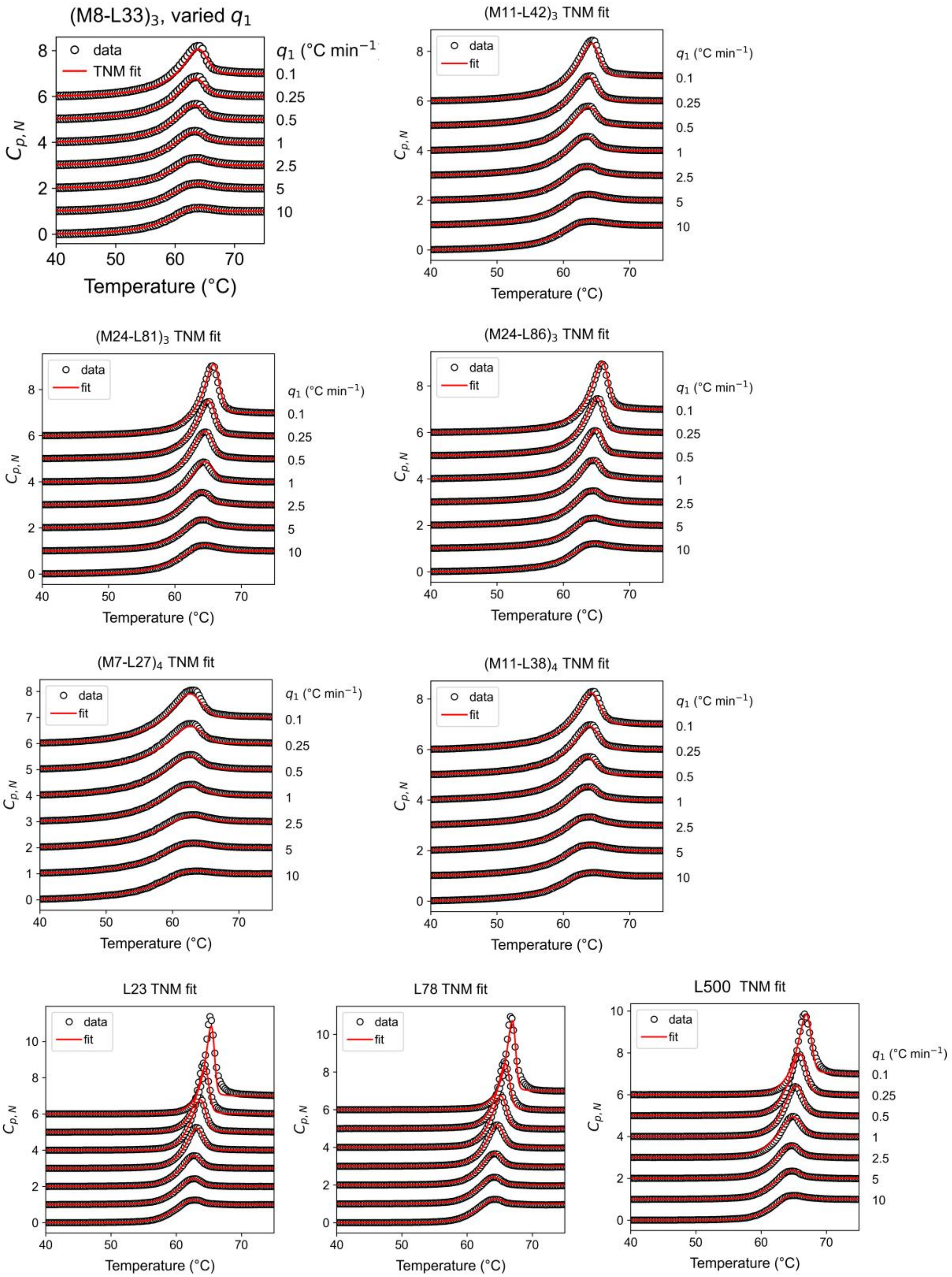

Figure S61, continued. Varied q_1 data overlaid with fits to the TNM model for three-arm star-blocks, four-arm star-blocks, and PLLA homopolymers. The data are presented as normalized heat capacity: $C_{p,N} = (C_p(T) - C_{p,g}(T)) / (C_{p,l}(T) - C_{p,g}(T))$. The traces are offset by one $C_{p,N}$ unit for visual clarity.

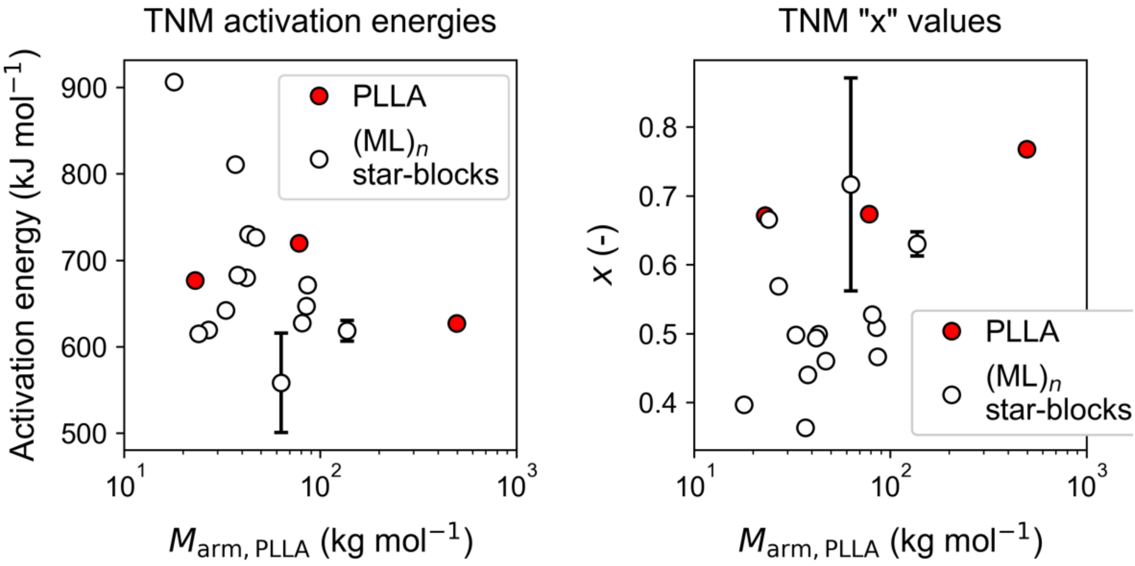

Figure S57. Activation energies and x parameters obtained by fitting fixed-q_1 reheating curves to the TNM model plotted as a function of $M_{\text{arm, PLLA}}$. No distinct architectural trends were apparent.

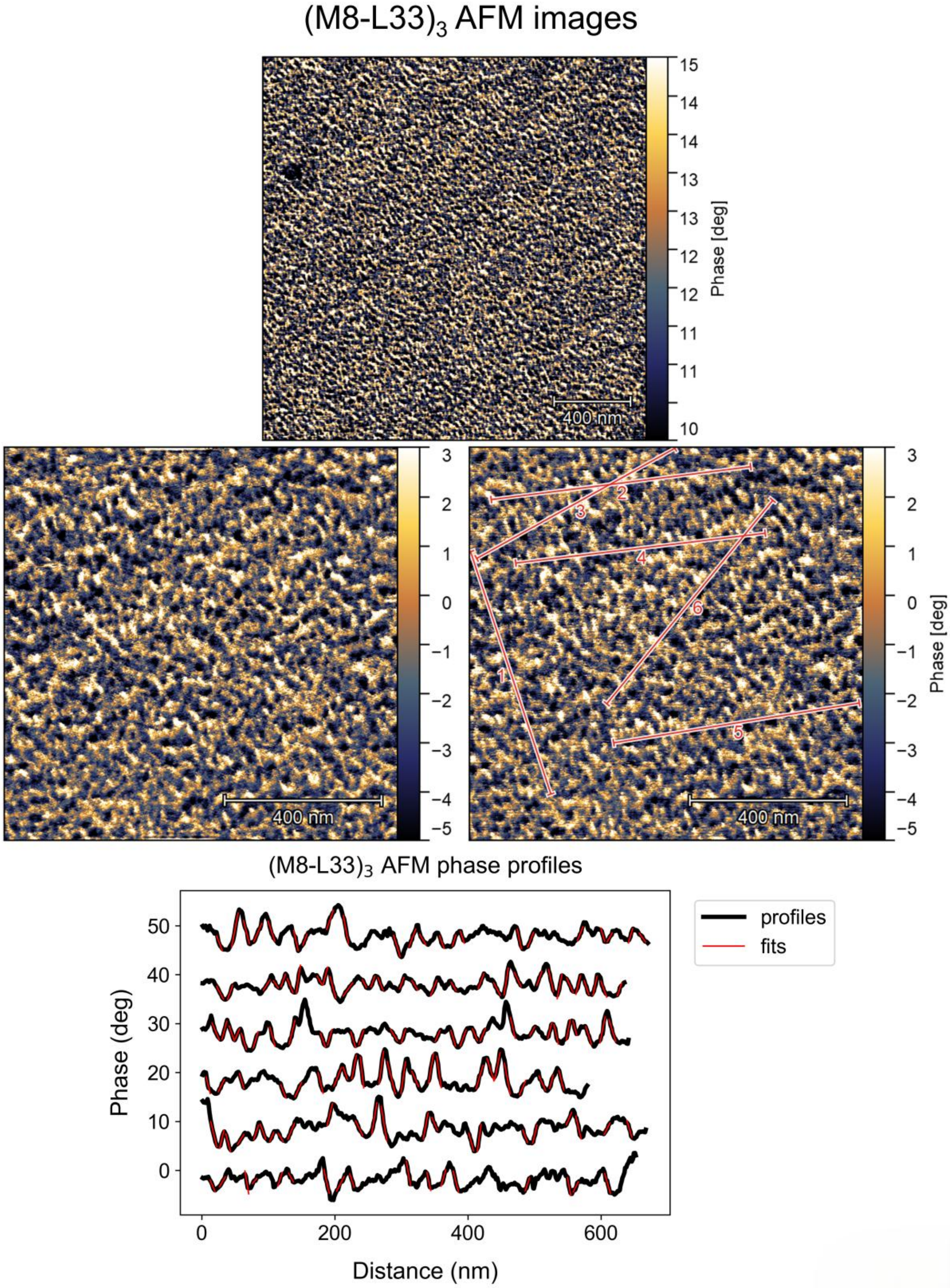

Figure S58. AFM phase images of (M8-L33)$_3$ rapidly quenched from the melt. Top scale = 2 x 2 µm, middle row scale = 1 x 1 µm. Bottom plot shows fitted interfacial regions superposed on phase line profile phase traces.

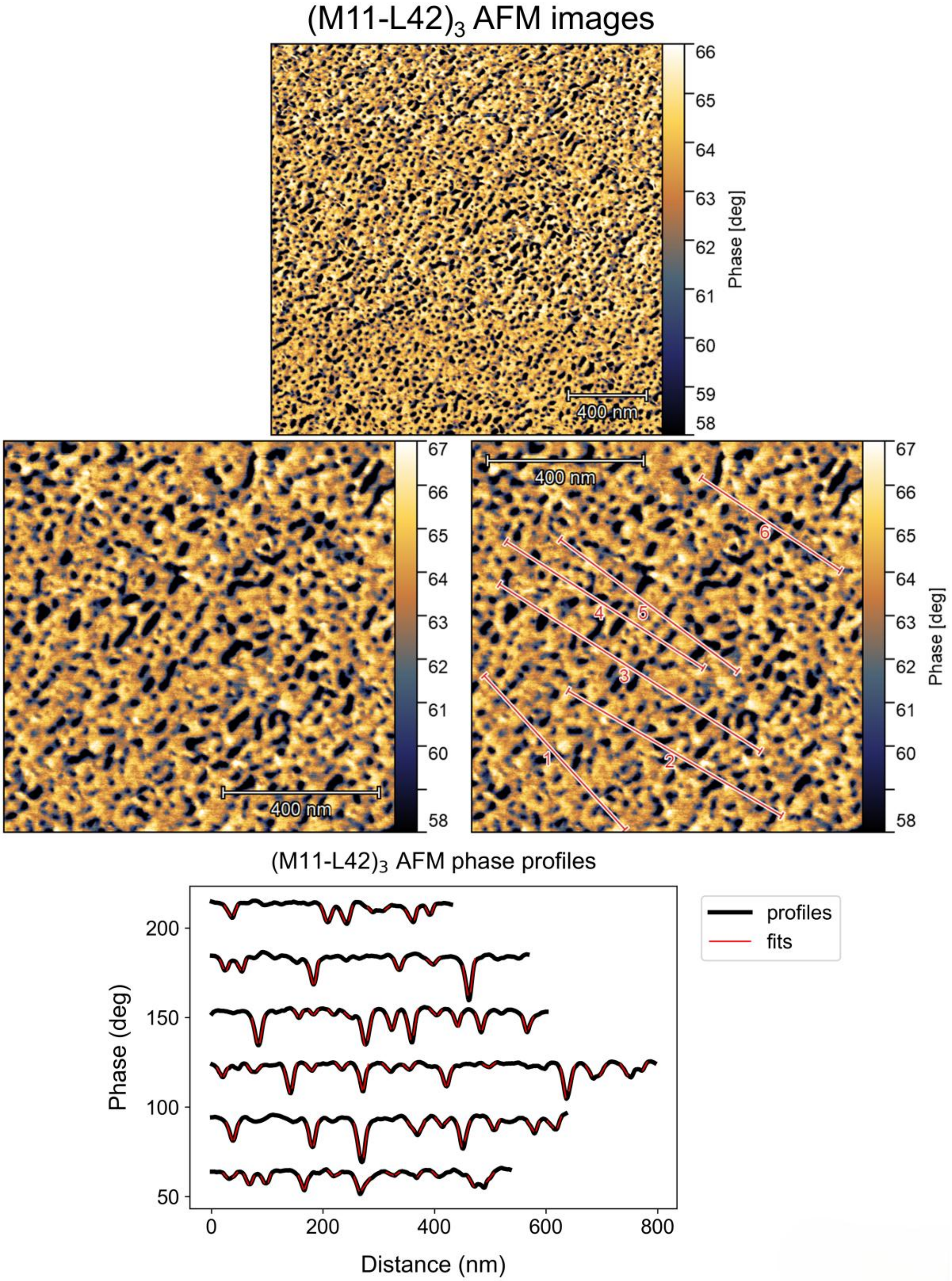

Figure S59. AFM phase images of (M11-L42)$_3$ rapidly quenched from the melt. Top scale = 2 x 2 µm, middle row scale = 1 x 1 µm. Bottom plot shows fitted interfacial regions superposed on phase line profile phase traces.

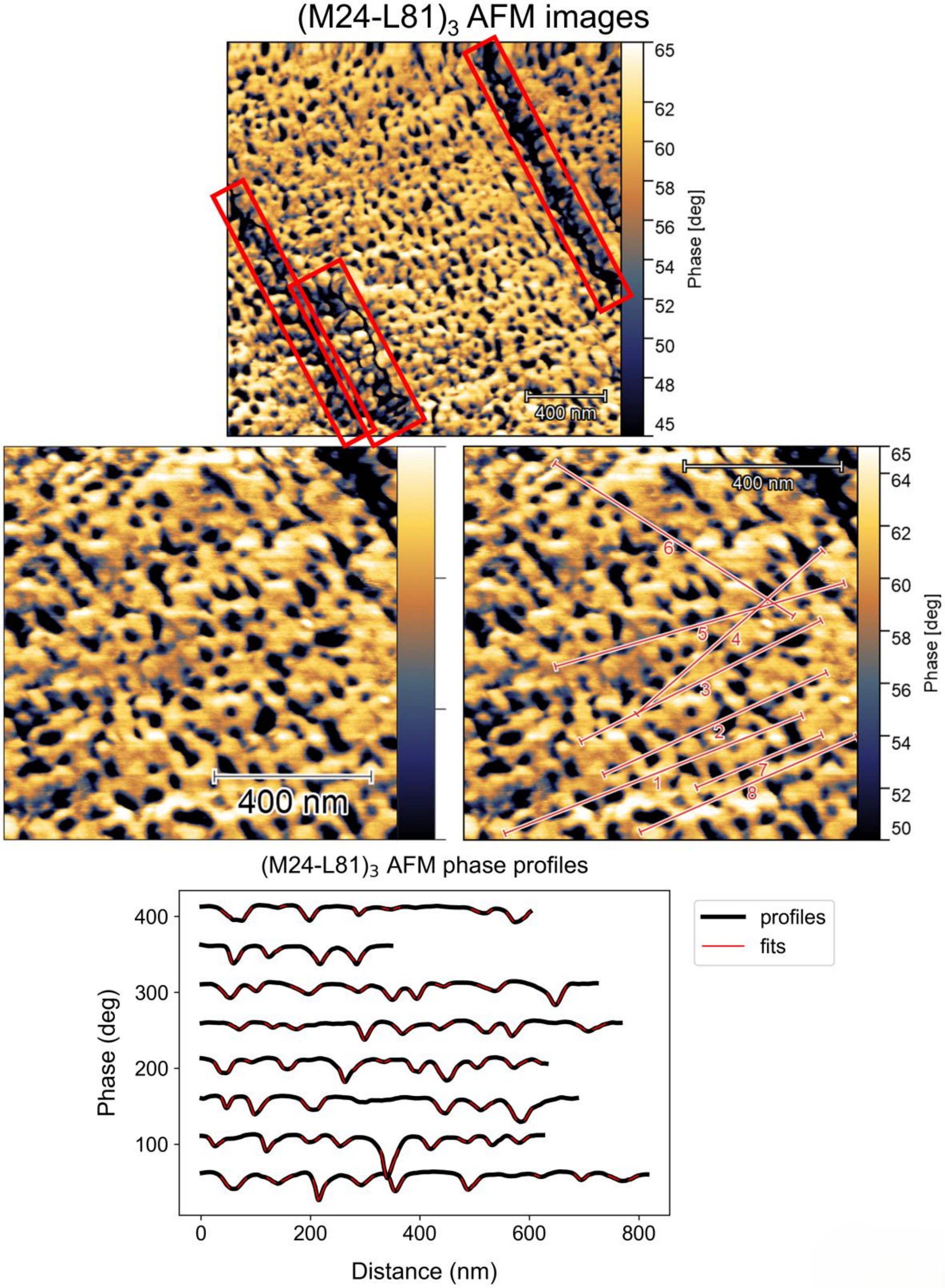

Figure S60. AFM phase images of (M24-L81)$_3$* rapidly quenched from the melt. Top scale = 2 x 2 µm, middle row scale = 1 x 1 µm. Red boxes enclose suspected knife marks. Bottom plot shows fitted interfacial regions superposed on phase line profile phase traces.

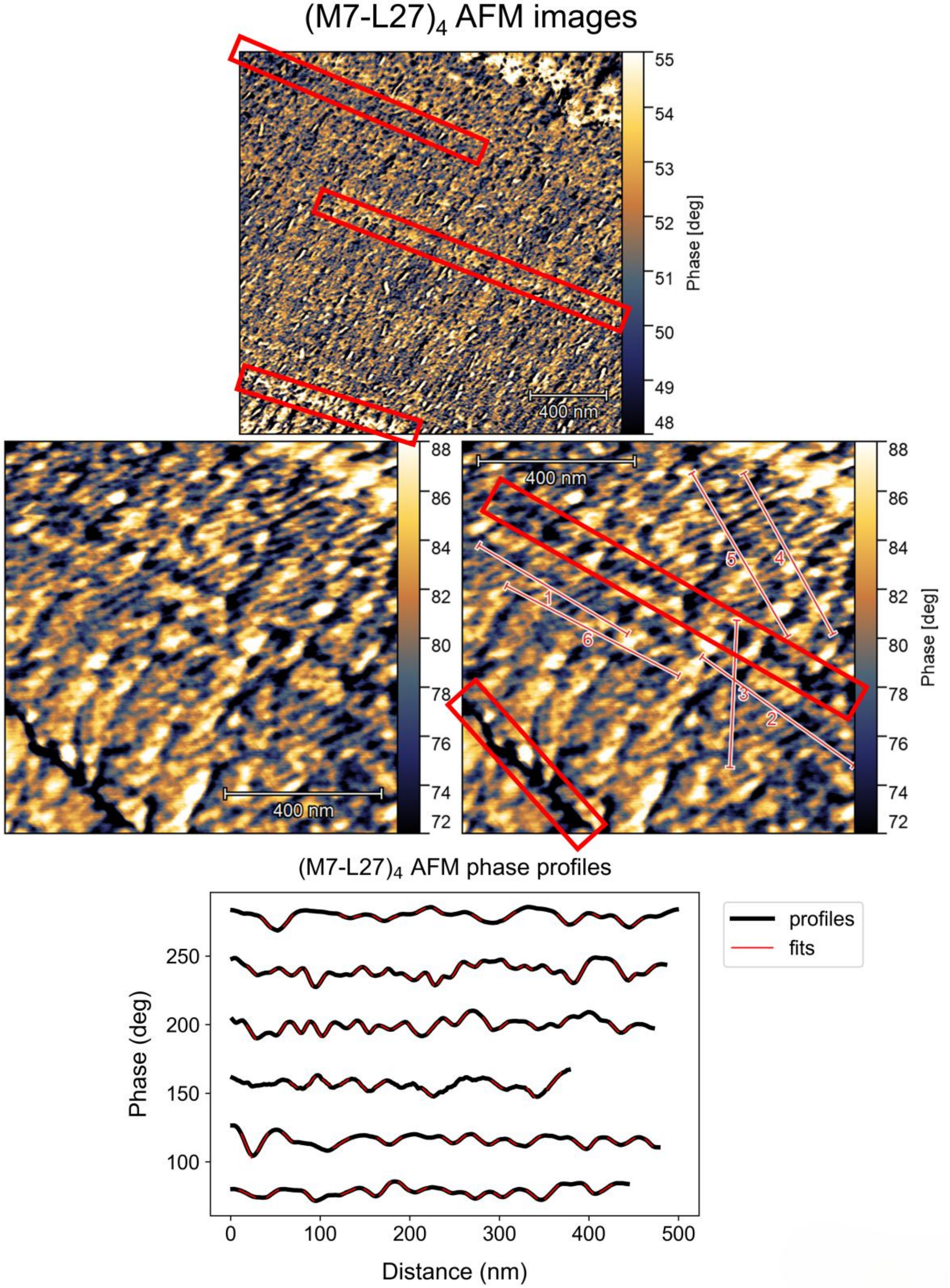

Figure S61. AFM phase images of $(M7\text{-}L27)_4$ rapidly quenched from the melt. Top scale = 2 x 2 µm, middle row scale = 1 x 1 µm. Red boxes enclose suspected knife marks. Bottom plot shows fitted interfacial regions superposed on phase line profile phase traces.

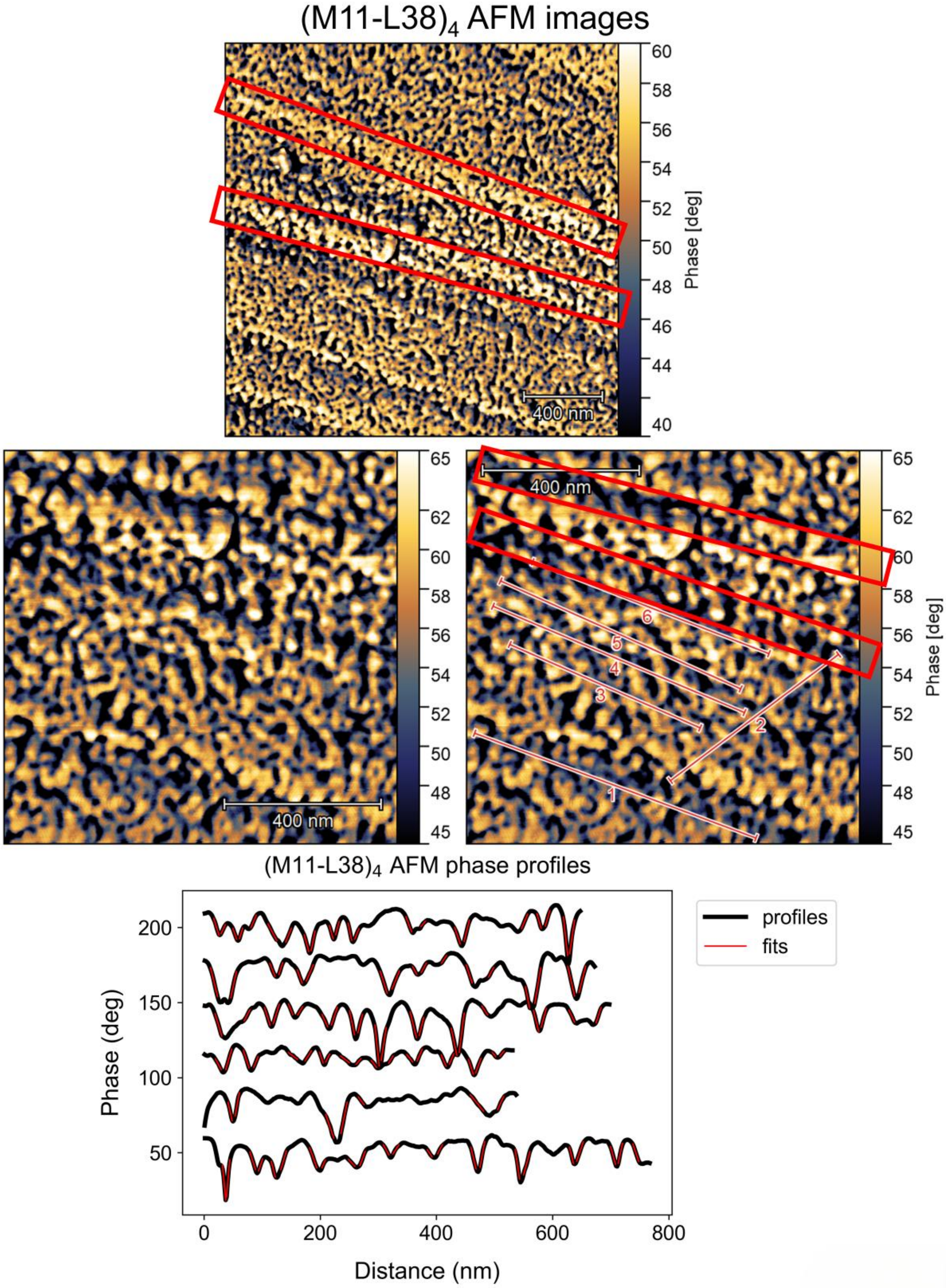

Figure S62. AFM phase images of (M11-L38)$_4$ rapidly quenched from the melt. Top scale = 2 x 2 µm, middle row scale = 1 x 1 µm. Red boxes enclose suspected knife marks. Bottom plot shows fitted interfacial regions superposed on phase line profile phase traces.

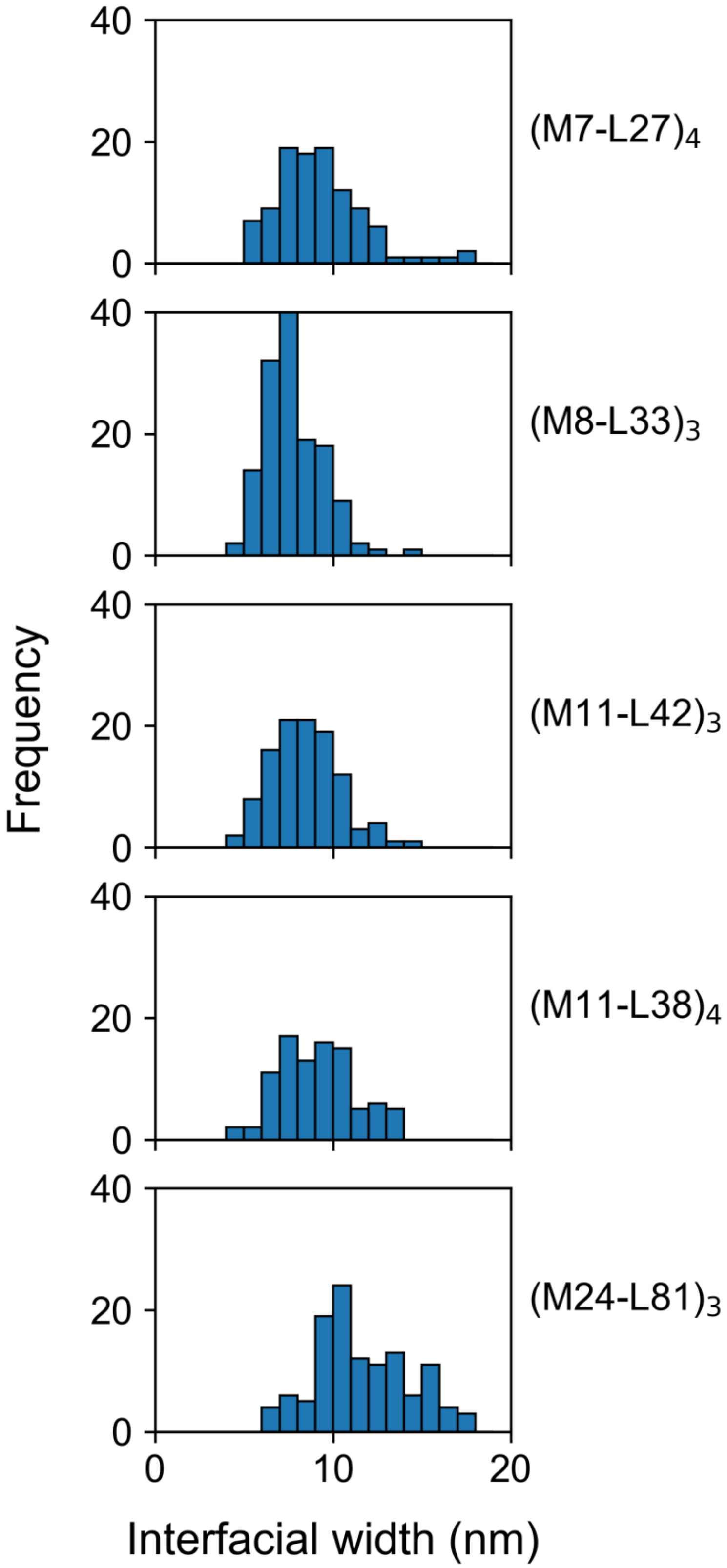

Figure S63. Histograms of interfacial widths obtained from AFM phase image line profiles.

Table S8. Comparison of (ML)$_n$ performance metrics with those of common packaging plastics. From left to right, the property columns are Young's modulus, yield stress, elongation at break, and heat distortion temperature (HDT). Commercial plastics data taken from the following websites:

https://www.matweb.com/reference/tensilestrength.aspx
https://www.matweb.com/reference/deflection-temperature.aspx
https://www.curbellplastics.com/resource-library/material-selection-tools/plastic-properties-table/?srsltid=AfmBOorNGubP5wkJY1hQwxbRF7ZLw9moU-xEl4T54UXwemX5nsb1iRwG

<table>
<tr><th>Material</th><th>E (GPa)</th><th>σ_y (MPa)</th><th>ε_b (%)</th><th>HDT (°C)</th></tr>
<tr><td>Poly(ethylene terephthalate)</td><td>2.7</td><td>79</td><td>125</td><td>70</td></tr>
<tr><td>Poly(carbonate)</td><td>2.6</td><td>66</td><td>100</td><td>140</td></tr>
<tr><td>High-density poly(ethylene)</td><td>0.8</td><td>27</td><td>500</td><td>85</td></tr>
<tr><td>Poly(propylene)</td><td>1.9</td><td>37</td><td>100</td><td>100</td></tr>
<tr><td>(M8-L33)$_3$, melt crystallized 5 min, aged 80 days</td><td>1.6</td><td>38</td><td>279</td><td rowspan="2">>140 (based on previous results in (ML)$_2$ triblocks of similar crystallinity[11]</td></tr>
<tr><td>(M7-L27)$_4$, melt crystallized 5 minutes, aged 80 8 days</td><td>1.8</td><td>36</td><td>238</td></tr>
</table>

References

(1) Chen, J.; Deng, C.; Hong, R.; Fu, Q.; Zhang, J. Effect of Thermal Annealing on Crystal Structure and Properties of PLLA/PCL Blend. *J. Polym. Res.* **2020**, *27* (8), 221. https://doi.org/10.1007/s10965-020-02206-1.

(2) Lorenzo, A. T.; Arnal, M. L.; Albuerne, J.; Muller, A. J. DSC Isothermal Polymer Crystallization Kinetics Measurements and the Use of the Avrami Equation to Fit the Data: Guidelines to Avoid Common Problems. *Polym. Test.* **2007**, *26* (2), 222–231. https://doi.org/10.1016/j.polymertesting.2006.10.005.

(3) Petrie, S. E. B. Thermal Behavior of Annealed Organic Glasses. *J. Polym. Sci. Part -2 Polym. Phys.* **1972**, *10* (7), 1255–1272. https://doi.org/10.1002/pol.1972.160100706.

(4) Koh, Y. P.; Simon, S. L. Enthalpy Recovery of Polystyrene: Does a Long-Term Aging Plateau Exist? *Macromolecules* **2013**, *46* (14), 5815–5821. https://doi.org/10.1021/ma4011236.

(5) Ma, M.; Huang, Y.; Guo, Y. Enthalpy Relaxation and Morphology Evolution in Polystyrene-b-Poly(Methyl Methacrylate) Diblock Copolymer. *Macromolecules* **2018**, *51* (18), 7368–7376. https://doi.org/10.1021/acs.macromol.8b01323.

(6) Narayanaswamy, O. S. A Model of Structural Relaxation in Glass. *J. Am. Ceram. Soc.* **1971**, *54* (10), 491–498. https://doi.org/10.1111/j.1151-2916.1971.tb12186.x.

(7) Yu, H.; Natansohn, A.; Singh, M. A.; Plivelíc, T. A Comparative Study Using Small-Angle X-Ray Scattering and Solid-State NMR of Microdomain Structures in

Poly(Styrene−butadiene−styrene) Triblock Copolymers. *Macromolecules* **1999**, *32* (22), 7562–7571. https://doi.org/10.1021/ma9910585.
(8) Kim, S. H.; Han, Y.-K.; Kim, Y. H.; Hong, S. I. Multifunctional Initiation of Lactide Polymerization by Stannous Octoate/Pentaerythritol. *Makromol. Chem.* **1992**, *193* (7), 1623–1631. https://doi.org/10.1002/macp.1992.021930706.
(9) Puchkov, A. A.; Sedush, N. G.; Buzin, A. I.; Bozin, T. N.; Bakirov, A. V.; Borisov, R. S.; Chvalun, S. N. Synthesis and Characterization of Well-Defined Star-Shaped Poly(L-Lactides). *Polymer* **2023**, *264*, 125573. https://doi.org/10.1016/j.polymer.2022.125573.
(10) Karidi, K.; Mantourlias, T.; Seretis, A.; Pladis, P.; Kiparissides, C. Synthesis of High Molecular Weight Linear and Branched Polylactides: A Comprehensive Kinetic Investigation. *Eur. Polym. J.* **2015**, *72*, 114–128. https://doi.org/10.1016/j.eurpolymj.2015.09.011.
(11) Krajovic, D. M.; Haugstad, G.; Hillmyer, M. A. Crystallinity-Independent Toughness in Renewable Poly(l-Lactide) Triblock Plastics. *Macromolecules* **2024**, *57* (6), 2818–2834. https://doi.org/10.1021/acs.macromol.3c02580.
(12) Zhang, Z.; Wan, X.; Fan, B.; Ma, Y.; Yang, B. Nonisothermal Crystallization Behavior and Enhanced Heat Resistance and Impact Toughness of Poly(l-Lactic Acid) with Bimodal Molecular Weight Distribution. *ACS Sustain. Chem. Eng.* **2022**, *10* (49), 16459–16469. https://doi.org/10.1021/acssuschemeng.2c06160.
(13) Matsen, M. W. Effect of Architecture on the Phase Behavior of AB-Type Block Copolymer Melts. *Macromolecules* **2012**, *45* (4), 2161–2165. https://doi.org/10.1021/ma202782s.
(14) Matsen, M. W.; Bates, F. S. Conformationally Asymmetric Block Copolymers. *J. Polym. Sci. Part B Polym. Phys.* **1997**, *35* (6), 945–952. https://doi.org/10.1002/(SICI)1099-0488(19970430)35:6%253C945::AID-POLB9%253E3.0.CO;2-G.
(15) Anderson, K. S.; Hillmyer, M. A. Melt Chain Dimensions of Polylactide. *Macromolecules* **2004**, *37* (5), 1857–1862. https://doi.org/10.1021/ma0357523.
(16) Witzke, David R. Introduction to Properties, Engineering, and Prospects of Polylactide Polymers, Michigan State University. https://doi.org/10.25335/8P45-Y156.
(17) Karavolias, M. Molecular Bottlebrushes: New Routes to Self-Assembled Morphologies with Small Periodicities, 2021. https://hdl.handle.net/11299/269976 (accessed 2025-06-06).
(18) Watts, A.; Kurokawa, N.; Hillmyer, M. A. Strong, Resilient, and Sustainable Aliphatic Polyester Thermoplastic Elastomers. *Biomacromolecules* **2017**, *18* (6), 1845–1854. https://doi.org/10.1021/acs.biomac.7b00283.
(19) Christie, D.; Register, R. A.; Priestley, R. D. Role of Chain Connectivity across an Interface on the Dynamics of a Nanostructured Block Copolymer. *Phys. Rev. Lett.* **2018**, *121* (24), 247801. https://doi.org/10.1103/PhysRevLett.121.247801.
(20) Semenov, A. N. Theory of Block Copolymer Interfaces in the Strong Segregation Limit. *Macromolecules* **1993**, *26* (24), 6617–6621. https://doi.org/10.1021/ma00076a047.
(21) Haugstad, G. *Atomic Force Microscopy: Understanding Basic Modes and Advanced Applications*; Atomic Force Microscopy: Understanding Basic Modes and Advanced Applications; 2012. https://doi.org/10.1002/9781118360668.
(22) Weidisch, R.; Michler, G. H.; Fischer, H.; Arnold, M.; Hofmann, S.; Stamm, M. Mechanical Properties of Weakly Segregated Block Copolymers: 1. Synergism on Tensile Properties of Poly(Styrene-*b*-*n*-Butylmethacrylate) Diblock Copolymers. *Polymer* **1999**, *40* (5), 1191–1199. https://doi.org/10.1016/S0032-3861(98)00196-7.

(23) Weidisch, R.; Stamm, M.; Schubert, D. W.; Arnold, M.; Budde, H.; Höring, S. Correlation between Phase Behavior and Tensile Properties of Diblock Copolymers. *Macromolecules* **1999**, *32* (10), 3405–3411. https://doi.org/10.1021/ma981748t.
(24) Argon, A. S.; Cohen, R. E.; Jang, B. Z.; Sande, J. B. V. Crazing in Two Polystyrene/Polybutadiene Block Copolymers. *J. Polym. Sci. Polym. Phys. Ed.* **1981**, *19* (2), 253–272. https://doi.org/10.1002/pol.1981.180190207.
(25) Deblieck, R. A. C.; van Beek, D. J. M.; Remerie, K.; Ward, I. M. Failure Mechanisms in Polyolefines: The Role of Crazing, Shear Yielding and the Entanglement Network. *Polymer* **2011**, *52* (14), 2979–2990. https://doi.org/10.1016/j.polymer.2011.03.055.
